# Controlled assembly of a bicyclic porphyrinoid and its 3-dimensional boron difluoride arrays†

**DOI:** 10.1039/d2sc01635d

**Published:** 2022-05-26

**Authors:** Weinan Zhou, Tridib Sarma, Liu Yang, Chuanhu Lei, Jonathan L. Sessler

**Affiliations:** School of Materials Science and Engineering, Shanghai University Shanghai 200444 China; Center for Supramolecular Chemistry and Catalysis, Department of Chemistry, College of Science, Shanghai University Shanghai 200444 China chlei@shu.edu.cn; Department of Chemistry, Cotton University Guwahati 781001 Assam India; Department of Chemistry, The University of Texas at Austin 105 East 24th Street, Stop A5300 Austin Texas 78712-1224 USA sessler@cm.utexas.edu

## Abstract

A fully conjugated cryptand-like bicyclic porphyrinoid ligand 4, incorporating three carbazole linkages and four dipyrrin moieties, was prepared *via* the acid-catalysed condensation of an extended 2,2′-bipyrrole analogue containing a central carbazole moiety and 3,4-diethyl-2,5-diformylpyrrole in 79% isolated yield. This new cryptand-like system acts as an effective ligand and allows for complexation of BF_2_ (boron difluoride) subunits. Three BODIPY arrays, containing two, three, and four BF_2_ subunits, namely 4·2BF_2_, 4·3BF_2_ and 4·4BF_2_, could thus be isolated from the reaction of 4 with BF_3_·Et_2_O in the presence of triethylamine at 110 °C, albeit in relatively low yield. As prepared, bicycle 4 is characterized by a rigid *C*_2_ symmetric structure as inferred from VT NMR spectroscopic analyses. In contrast, the three BODIPY-like arrays produced as the result of BF_2_ complexation are conformationally flexible and unsymmetric in nature as deduced from similar analyses. All four products, namely 4, 4·2BF_2_, 4·3BF_2_ and 4·4BF_2_, were characterized by means of single crystal X-ray diffraction analyses. Tetramer 4·4BF_2_ gives rise to a higher extinction coefficient (by 2.5 times) relative to the bis- and tris-BODIPY arrays 4·2BF_2_ and 4·3BF_2_. This was taken as evidence for stronger excitonic coupling in the case of 4·4BF_2_. All three BODIPY-like arrays proved nearly non-fluorescent, as expected given their conformationally mobile nature. The efficiency of reactive oxygen species (ROS) generation was also determined for the new BODIPY arrays of this study.

## Abstract 2

A cryptand-like bicyclic porphyrinoid was obtained in preference over the monocyclic porphyrinoid by controlling the reaction stoichiometry and condensation conditions. The cryptand-like species supports formation of multiple 3D BODIPY-like arrays.

## Graphical abstract

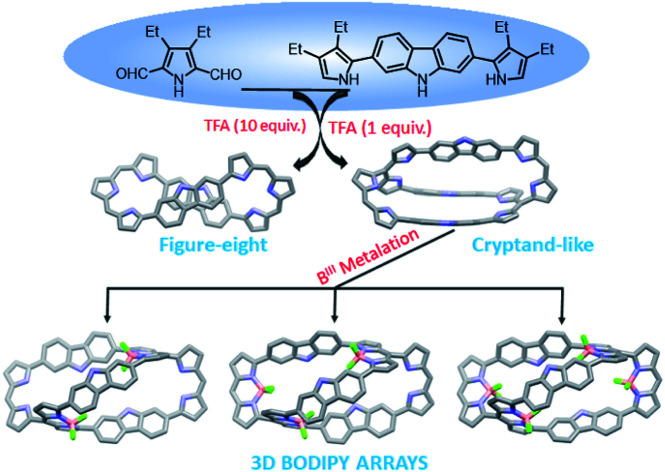

## Introduction

**Scheme 1:**
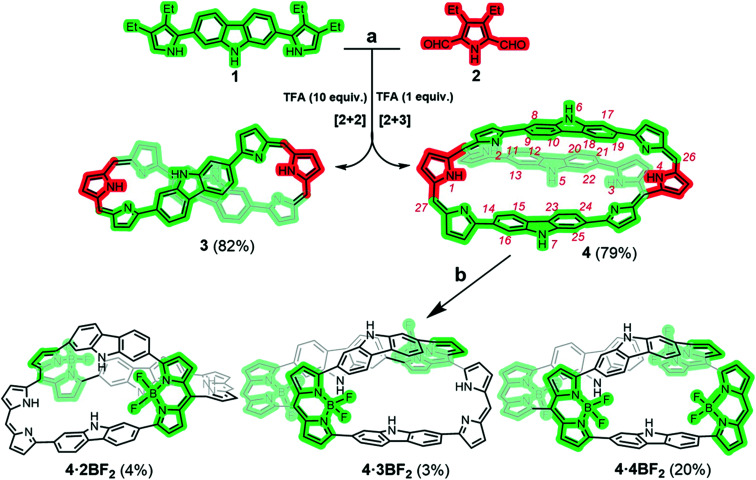
Synthesis of the figure-of-eight monocyclic porphyrinoid 3 and the bicyclic porphyrinoid 4 and its corresponding BODIPY arrays (4·2BF_2_, 4·3BF_2_, and 4·4BF_2_). Reaction conditions: a) TFA, DDQ, DCM, RT; b) BF_3_·Et_2_O, TEA, toluene, 110 °C. β-Pyrrolic ethyl groups are omitted in 3, 4, and the BF_2_ derivatives for clarity.

Porphyrinoid scaffolds^1–3^ continue to attract tremendous attention on the part of the synthetic community, as befits their study in applications running the gamut from clinical medicine to materials chemistry.^4–12^ Over the past few decades, a large number of increasingly elegant porphyrinoid-based macrocycles have been reported.^13–15^ However, fully conjugated topographically nonplanar oligopyrroles, so-called three dimensional (3D) porphyrinoids, remain rare.^16–19^ For example, in 2008 Setsune and co-workers described a pyridine-containing cryptand-like bicyclic hexapyrrole with three dipyrrylpyridine linkages that demonstrated positive cooperativity in binding carboxylic acids.^20^ Our own group also reported a fully conjugated 3D carbaporphyrinoid cage encompassing a dibenzo[*g*,*p*]chrysene moiety.^17^ More recently, fully conjugated macrobicyclic porphyrinoids have also provided experimental support for Baird aromaticity^16^ and have allowed stabilization of organic radical frameworks.^18^ However, the synthesis of three dimensional porphyrinoids skeleton remains challenging and can be hampered by poor yields resulting from metal-mediated reactions^18^ or the concomitant generation of monocyclic or polymeric by-products.^17,19,20^ Here we report that by condensing a carbazole-containing 2,2′-bipyrrole analogue 1 with a 2,5-diformylpyrrole one can obtain either a monocyclic figure-eight porphyrinoid 3 in 82% yield or a bicyclic cryptand-like porphyrinoid 4 in 79% yield depending on the choice of conditions (Scheme 1). The 3D system 4, which is the focus of the present report, contains four co-planar dipyrrin units. Accordingly, it may be converted to the corresponding bis-, tris-, and tetrakis-BF_2_ complexes. These latter BODIPY-like arrays were characterized by single crystal X-ray diffraction analyses, various spectroscopies, and electrochemical methods.

Recently, covalent arrangements of BODIPYs (BF_2_-dipyrromethenes), including oligomers and arrays,^21–26^ have attracted attention as complements to simple monomeric BODIPYs. The strong exciton coupling^22,24^ expected for systems containing more than one BODIPY-like subunit has made such systems of interest in the context of specific applications, including light-harvesting, fluorescence-based sensor development, and photodynamic therapy (PDT).^27–33^ Recently, Werz and co-workers prepared a series of benzene-fused oligo-BODIPYs containing up to 31 rings that exhibited intriguing NIR-absorbing and redox properties.^34^ Early on several macrocyclic BODIPY arrays incorporating 1,4- and 1,3-phenylene moieties were reported by Nabeshima and co-workers, which were shown to act as hosts for cationic guests.^35,36^ Our own group reported two giant calix[8]- and calix[16]phyrin-derived BF_2_ arrays with strong NIR absorption features.^22^ However, to the best of our knowledge, there are no reports concerning covalently linked three-dimensional conjugated BODIPY arrays. Accordingly, their photophysical properties remain unexplored.

## Results and discussion

We postulated that carbazole-containing pyrrolic precursors, building blocks we have recently exploited to produce a carbadecaphyrin^37^ and a cobalt(ii) metallocage structure,^38^ might allow construction of a 3D porphyrinoid containing co-planar dipyrrin units suitable for BF_2_ complexation. The present study was undertaken as a test of this hypothesis. In fact, both a figure-of-eight monocyclic porphyrinoid 3 and a fully conjugated cryptand-like bicyclic porphyrinoid 4 could be formed *via* a controllable MacDonald-type condensation using such a precursor (Scheme 1).

In an initial study, equal molar quantities of 1 and 2 were condensed in the presence of 2.5 equiv. of trifluoroacetic acid (TFA) followed by oxidation with DDQ. The crude reaction mixture was subjected to alumina column chromatography using 50% CH_2_Cl_2_/*n*-hexane as the eluent; this gave the monocyclic product 3 as a green-coloured fraction in 45% yield along with bicycle 4 as a dark-coloured fraction in considerably lower yield. Evidence for the formation of 4, our desired synthetic target, came from a MALDI-TOF mass spectrometric analysis, which revealed a mass peak at 1511.5491 amu consistent with the molecular formula expected for 4 (C_104_H_107_N_11_ + H^+^) (Fig. S29 and 30†). Unfortunately, 3 and 4 proved difficult to separate cleanly by alumina column chromatography. Thus, an effort was made to optimize the reaction conditions. It was found that reacting 1 and 2 in 2 : 1 molar ratio in the presence of 1.0 equiv. of TFA yielded the bicyclic product 4 in 79% yield free of observable quantities of 3. Conversely, when 1 and 2 were condensed in a 1 : 1 molar ratio in the presence of 10.0 equiv. of TFA the monocyclic porphyrinoid 3 was obtained exclusively in 82% yield (*cf.* Table S1†).

**Fig. 1:**
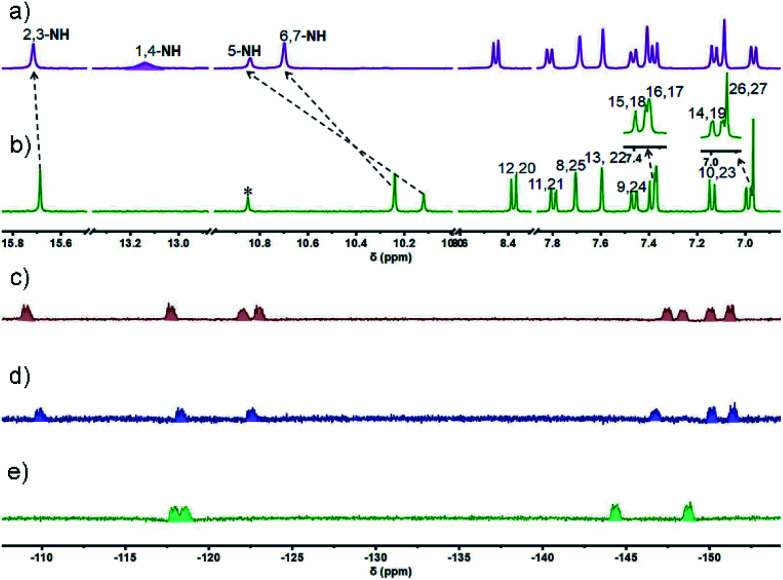
Comparative ^1^H NMR spectra (aromatic region) of ligand 4 recorded in THF-*d*_8_ at a) −70 °C and b) 25 °C and the ^19^F NMR spectra of c) 4·4BF_2_, d) 4·3BF_2_, and e) 4·2BF_2_ recorded in THF-*d*_8_ at −80 °C.

The ^1^H NMR spectrum of 4 recorded in THF-*d*_8_ at room temperature was characterized by the presence of well resolved signals consistent with a symmetric system (Fig. 1b). Three singlets at 15.69, 10.24, and 10.12 ppm are seen that are ascribed to the NH(2,3), NH(6,7), and NH(5) protons, respectively (*cf.*Scheme 1 for atom numbering). As expected, the NH protons were found to undergo exchange in the presence of D_2_O (Fig. S2†). The pyrrolic NH(1,4) proton signal was not observed, presumably due to its fast exchange with the imine moieties at room temperature; however, this signal, integrating to two protons, appeared at 13.19 ppm when the spectrum was recorded at 203 K (Fig. 1a and S7†). The substantial downfield shift of these protons relative to the precursors (*i.e.*, 7.55 ppm in 1 ^37^ and 9.74 ppm in 2 ^39^) is consistent with strong intramolecular hydrogen bonding interactions between the dipyrromethene units.

Another ten sets of signals were seen, including six doublets and four singlets in the aromatic region, each of which integrated to two protons; these signals were ascribed to the carbazole –CH protons at 8.38 (H(12,20)), 7.80 (H(11,21)), 7.70 (H(8,25)), 7.60 (H(13,22)), 7.46 (H(9,24)), 7.39(H(15,18)), 7.37 (H(16,17)), 7.14 (H(10,23)), 6.99 (H(14,19)), and meso –CH protons signal at 6.97 ppm (H(26,27)), respectively. These peak assignments were made with the aid of ^1^H–^1^H COSY and 2D ROESY analyses (Fig. S5 and S6†). Variable temperature (VT) NMR spectral analyses over the −90–25 °C range were also carried out in THF-*d*_8_ (Fig. S3 and S4†). On the basis of these studies, we infer that in solution cryptand 4 is conformationally rigid.

**Fig. 2:**
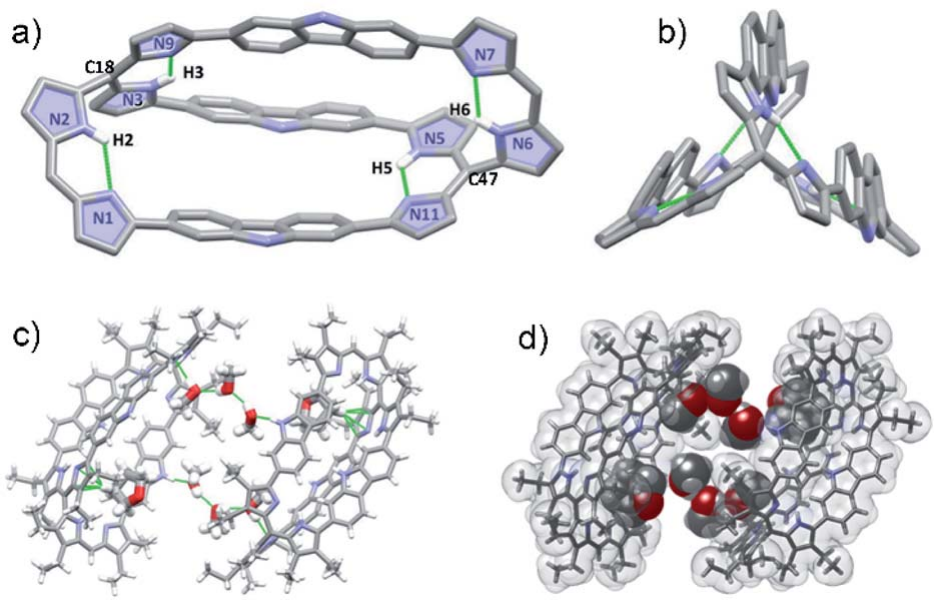
Single crystal X-ray structures of 4. a) Top and b) side views. Atom colour key: carbon (light grey), nitrogen (light purple), hydrogen (white). Hydrogen atoms that are not involved in intramolecular interactions, β-pyrrolic alkyl groups and solvent molecules have been omitted for clarity. c) and d) Molecular packing arrangement in stick and space-filling form, respectively, showing six methanol and two THF molecules encapsulated within a self-assembled dimer.

Diffraction grade single crystals of 4 were obtained *via* the slow diffusion of methanol into a THF solution. The resulting X-ray structure confirmed that in the solid state 4 possess a cryptand-like shape with *C*_2_ molecular symmetry (Fig. 2). Bicycle 4 has an ellipsoidal cavity and the two bridgehead C atoms (C18–C47) are separated by a distance of 13.611 Å. Three carbazole NH moieties orient towards the same side of the cryptate cavity and two of the three carbazole planes are nearly parallel and tilted towards the third one at angles of 36.94 and 38.95°, respectively (Fig. S34†). The pyrrolic amine and imine are involved in apparent intramolecular hydrogen bonding interactions as reflected in bond distances and angles of 2.111 Å and 125.09° for N2–H2⋯N1, 1.952 Å and 127.63° for N3–H3⋯N9; 1.957 Å and 127.83° for N5–H5⋯N11, and 2.078 Å and 125.92° for N6–H6⋯N7, respectively (Fig. 2a).

Further inspection of the crystal structure revealed the presence of six molecules of methanol and two molecules of THF encapsulated within the cavity of a dimer created *via* the self-assembly of 4. These guests are held in place *via* multiple hydrogen bonding and CH⋯π interactions, respectively (Fig. 2c and d).

Another structural feature revealed by the X-ray diffraction analysis is that the dihedral angles between the pyrrole rings in each dipyrromethene subunit (*i.e.*, N1 and N2, N3 and N9, N5 and N11, N6 and N7) are small (*i.e.*, 2.47 to 7.3°; Fig. S33†). In light of this finding, reflecting dipyrrin subunits that are nearly planar, we attempted to use 4 as a platform for preparing BODIPY-like arrays. With this goal in mind, bicycle 4 was treated with BF_3_·Et_2_O in the presence of triethylamine at 110 °C; this afforded the corresponding di-, tri-, and tetrakis-BF_2_ complexes, 4·2BF_2_, 4·3BF_2_, and 4·4BF_2_, respectively, albeit in low yield (Scheme 1 and S1†).

MALDI-TOF MS analyses revealed peaks corresponding to the expected compositions of the di-, tri-, and tetrameric BODIPY arrays (Fig. S31–S33†). The molecular structures of 4·2BF_2_, 4·3BF_2_, and 4·4BF_2_ were established on the basis of ^1^H, ^19^F, and ^11^B NMR spectroscopic analyses. In contrast to 4, the VT ^1^H NMR spectra of all these BODIPY arrays recorded in THF-*d*_8_ displayed features consistent with a degree of conformational flexibility on the NMR time scale; however, all expected signals were visible at lower temperatures (*cf.* Fig. S10, S16 and S22†). For instance, in the case of 4·4BF_2_, the ^1^H NMR spectrum contained a set of peaks integrating to two protons that were buried in the baseline at room temperature (Fig. S21†). This signal was readily visible at −40 °C with the overall spectrum reflecting an unsymmetric structure (Fig. S23†). The absence of pyrrolic NH signals was taken as further evidence that in 4·4BF_2_, the dipyrrin units are coordinatively saturated.

Further support for the conformational flexibility of the BODIPY arrays came from ^19^F NMR spectral studies. For instance, in the ^19^F NMR spectrum of 4·4BF_2_, a relatively small number of signals is seen at ambient temperature as would be expected for a system subject to conformational dynamics (Fig. S25†). Upon lowering the temperature to −80 °C, eight unresolved resonances ascribable to the four BF_2_ subunits appeared at −108.93, −117.59, −122.05, −122.87, −147.58, −148.28, −150.16, and −151.34 ppm, respectively, as would be expected for a conformationally locked form of 4·4BF_2_ wherein the fluorine atoms in 4·4BF_2_ are magnetically inequivalent (Fig. 1c).

The ^11^B NMR spectrum was characterized by the presence of two poorly resolved triplets at −0.28 and −0.65 ppm (Fig. S26†). Similar spectral patterns were also observed for 4·2BF_2_ and 4·3BF_2_. We thus suggest that in solution all three BODIPY arrays, 4·2BF_2_, 4·3BF_2_, and 4·4BF_2_, are conformationally mobile with this inferred motion likely resulting from the carbazole linkage undergoing ‘flipping’ on the NMR timescale. This behaviour contrasts with that of 4, which exists as a relatively rigid and symmetric structure in THF-*d*_8_ solution. While further study is required, the lack of conformational mobility seen for 4 could reflect the fact that it interacts with the solvent (*i.e.*, captures THF molecules). Such binding behaviour would be expected to inhibit molecular motion.

**Fig. 3:**
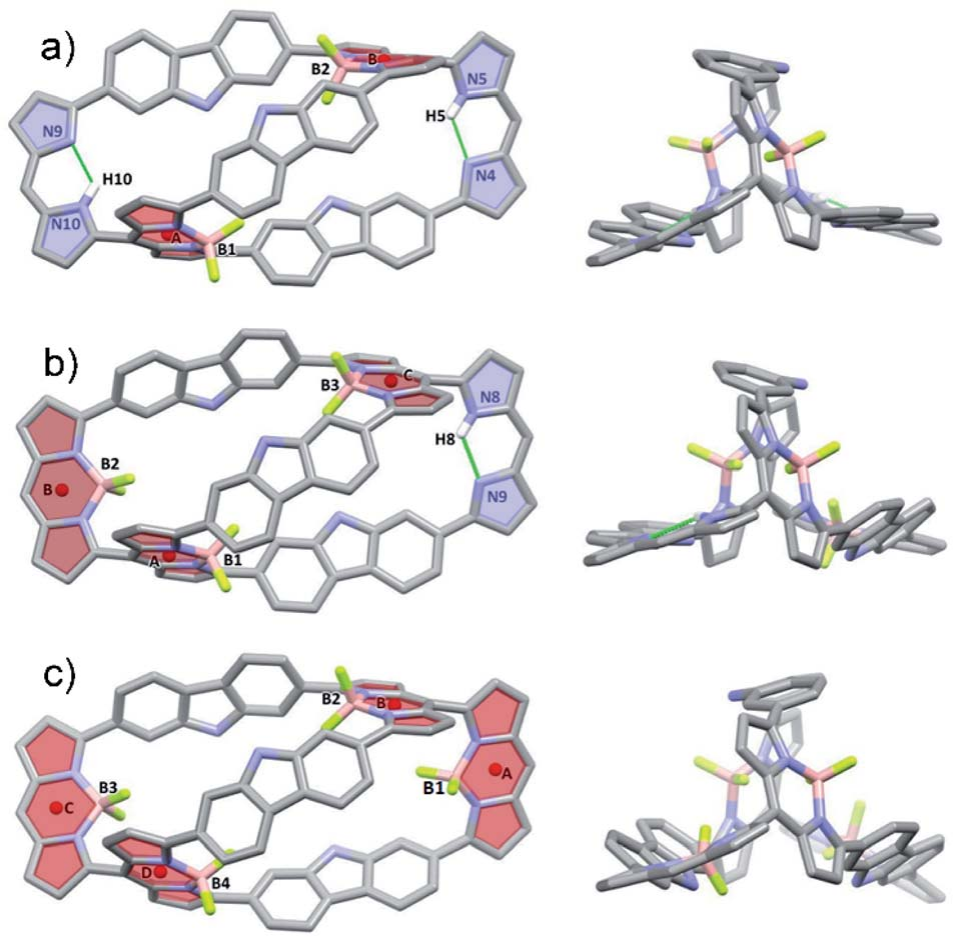
Single crystal X-ray structures of BODIPY arrays. Atom colour key: carbon (light grey), nitrogen (light purple), hydrogen (white), boron (light pink), fluorine (yellow green). Hydrogen atoms that are not involved in intramolecular interactions, β-pyrrolic alkyl groups and solvent molecules have been omitted for clarity. a) 4·2BF_2_, b) 4·3BF_2_, and c) 4·4BF_2_.

Further insights into the molecular structure of the BODIPY arrays came from single-crystals X-ray structural analyses. Fig. 3 shows the molecular structures of 4·2BF_2_, 4·3BF_2_ and 4·4BF_2_. All three systems are bicyclic and bear structural analogy to 4. For instance, the bis-BODIPY analogue, 4·2BF_2_, is pseudo-D_2_-symmetric with the two BF_2_ units bound to the meso-substituted dipyrrin moieties in a diagonal arrangement. The unbound dipyrrin units are engaged in intramolecular hydrogen bonding interactions characterized by bond distances and angles of 2.108 Å and 123.57° and 2.257 Å and 122.23° for N5–H5⋯N4 and N10–H10⋯N9, respectively (Fig. 3a). The centroid-to-centroid distance between the chromophore units (defined by the C_9_N_2_B planes indicated as A and B) is 10.475 Å. Five methanol molecules, solvents of crystallization, are observed in each unit (Fig. S37†). Two of these methanol molecules are found trapped in the cryptand-like void of 4·2BF_2_ being stabilized *via* presumed OH⋯F and NH⋯O H-bonding interactions; two others are H-bonded (NH⋯O) to the carbazole units, while the final methanol molecule does not appear to interact directly with 4·2BF_2_.

In the case of the tris-BODIPY 4·3BF_2_ BODIPY a shorter centroid-to-centroid distance between the adjacent chromophore units (defined by C_9_N_2_B planes) of 4.94 Å (A and B) was observed (Fig. 3b), whereas the solid-state structure of 4·4BF_2_ revealed the presence of two pair of orthogonally aligned BODIPY-like arrays characterized by dihedral angles of 84.84 and 86.30° (Fig. 3c and S38†). Centroid to centroid distances between the adjacent BODIPY units (defined by the respective C_9_N_2_B planes) of 4.942 and 4.983 Å were found (Fig. S37†). Five methanol molecules of crystallization are also observed in the solid-state structure of 4·4BF_2_ in analogy to what was seen in the case of 4·2BF_2_ (Fig. S36†).

All three BODIPY arrays contain six C–C bonds that serve to join the three carbazole units to the adjacent pyrrole rings. These C–C bonds were found to have significant single bond character based on the bond distances (ranging from 1.467 to 1.495 Å). Such a finding is consistent with the free rotation inferred from the solution phase NMR spectral studies (*vide supra*, Fig. S42†).

**Fig. 4:**
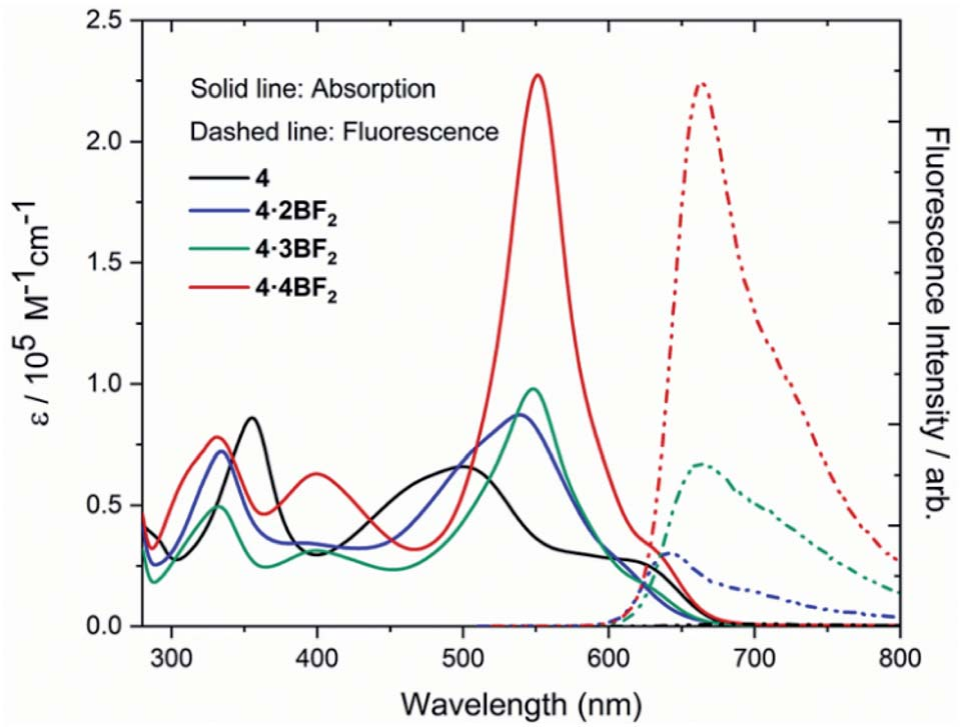
UV-Vis absorption and emission spectra of ligand 4, 4·2BF_2_, 4·3BF_2_, and 4·4BF_2_ recorded in toluene at room temperature.

The absorbance and luminescent properties of ligand 4 and corresponding BODIPY arrays 4·2BF_2_, 4·3BF_2_, and 4·4BF_2_ were measured in toluene (*cf.*Fig. 4 and Table 1). As the degree of BF_2_ substitution increases, both the absorption and emission spectra were found to red-shift slightly, and the extinction coefficients and fluorescence emission intensities were found to increase. The tetrakis system 4·4BF_2_ shows the most intense absorption band (*λ*_max_ = 551 nm) with an extinction coefficient, *ε* = 2.27 × 10^5^ M^−1^cm^−1^, which is *ca.* 2.5-fold larger than those for the corresponding bis- and tris-BODIPY arrays. In the case of 4·4BF_2_ (and to a much lesser extent, 4·3BF_2_), a high energy absorption feature is seen at 400 nm. These spectral features are taken as evidence of interchromophore exciton coupling between the two pair of orthogonally-aligned BODIPY subunits present in 4·4BF_2_.^28,40^

**Table 1:** Selected photophysical^a^ and electrochemical^b^ properties for cryptand ligand 4 and corresponding BODIPY arrays

Compound	*λ*_max_ [nm] (*Ɛ* [×10^5^ L mol^−1^ cm^−1^])	Fluorescence			*E*_ox.3_	*E*_ox.2_	*E*_ox.1_	*E*_red.1_	*E*_red.2_	*E*_red.3_
		*λ*_max_/nm^c^	*τ*/ns^d^	*ϕ*_F_ e						
4	500 (0.66)	—	—	—	0.71	0.45	0.33	−1.22	−1.78	−1.95
4·2BF_2_	539 (0.87)	640	4.87	0.03%	0.95	0.82	0.58	−1.18	−1.48	−1.93
4·3BF_2_	548 (0.98)	660	2.60	0.14%	0.85	0.69	0.56	−1.15	−1.39	−1.73
4·4BF_2_	551 (2.27)	664	4.01	0.25%	0.87	0.70	0.57	−1.18	−1.69	—

a UV-Vis absorption spectra were recorded in toluene at room temperature.

b Cyclic voltammetry studies were conducted in CH_2_Cl_2_ containing 0.1 M *n*-Bu_4_NPF_6_ as the supporting electrolyte; Ag/Ag^+^ was used as the reference electrode, a Pt wire was used as the counter electrode, and glassy carbon was used as the working electrode. Potentials were recorded *vs.* ferrocene/ferrocenium ion. Scan rates were 0.05 V s^−1^. These potentials were determined by differential pulse voltammetry.

c Fluorescence emission spectra were recorded in toluene (10^−5^ M) at room temperature.

d Fluorescence lifetime.

e Absolute fluorescence quantum yield.

The electronic absorption spectral features of ligand 4 and its BODIPY-like derivatives were further evaluated by time dependent density functional theory (TD-DFT) calculations at the CAM-B3LYP/6-311G(d,p) level.^41^ Across the board, these TD-DFT studies revealed a good correspondence between the observed and predicted spectral features (Fig. S64–S67 and Table S5–S8†).

All BODIPY arrays proved relatively non-fluorescent in various organic solvents (quantum yields < 1%). In toluene, the absolute fluorescence quantum yields (*ϕ*_FL_) were determined to be 0.03%, 0.14%, and 0.25% for 4·2BF_2_, 4·3BF_2_, and 4·4BF_2_, respectively. This lack of appreciable fluorescence in the case of the BODIPY arrays is ascribed to their conformational mobility as revealed in the VT NMR spectral studies discussed above. Such a molecular property would be expected to result in an enhanced rate of nonradiative decay.^42^ When the polarity of the solvent increased (*i.e.*, upon changing from toluene to methanol) slight changes in the absorption features were seen for 4·2BF_2_, 4·3BF_2_, and 4·4BF_2_, whereas an apparent decrease in the fluorescence intensity was observed (Fig. S43–S46†). This qualitative finding may reflect a degree of symmetry-breaking internal charge transfer (ICT) in the more polar and better bound solvents.^43,44^

The fluorescence lifetimes of the BODIPY arrays were determined in toluene by means of time-correlated single photo counting (TCSPC) with photo-excitation at 450 nm (Fig. S48†). The fluorescence decay profiles could be fitted to a double exponential function corresponding to two decay constants of 0.7 ns (36%) and 4.87 ns (64%) for 4·2BF_2_, 0.78 ns (15%) and 2.6 ns (85%) for 4·3BF_2_, and 1.86 ns (21%) and 4.01 ns (79%) for 4·4BF_2_, respectively. The biphasic decay pattern is consistent with the presence of two excited-state components that likely reflect the inherent molecular flexibility. Such findings provide support for the inference that all three 3D BODIPY arrays preserve their linear, orthogonally-aligned features in the photoexcited state.^45,46^

**Fig. 5:**
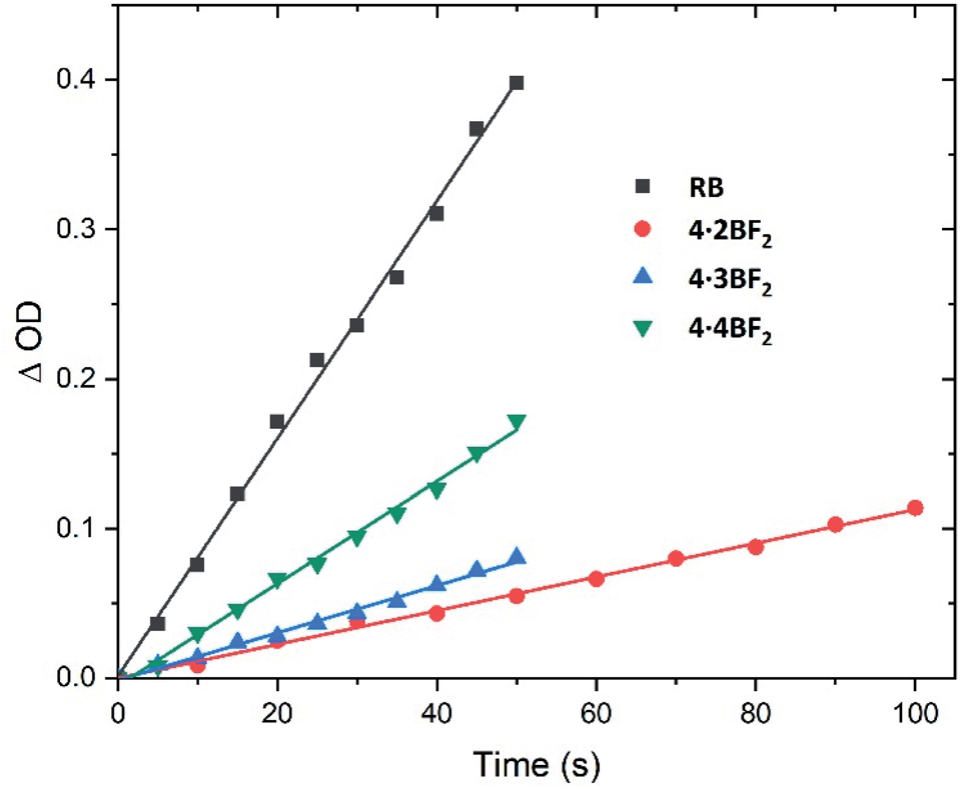
Plots of the change in absorbance of DPBF at 414 nm against irradiation time (*λ*_irr_ = 520 nm) seen in the presence of the BODIPY arrays of this study in CH_2_Cl_2_ at room temperature. RB in methanol was used as a refence standard. These plots were used to calculate the singlet oxygen (^1^O_2_) quantum yields (*cf.* eqn S1 in the ESI†).

Inspired by previously reported orthogonally-aligned BODIPY dimers or oligomers that produced reactive oxygen species (ROS) upon photoexcitation as the result of inferred spin–orbit charge transfer intersystem crossing (SOCT-ISC),^47,48^ we investigated their singlet oxygen generation efficiency under conditions of green light (*ca.* 520 nm) irradiation. Here, 1,3-diphenylisobenzofuran (DPBF) and Rose Bengal (RB) were used as the singlet oxygen scavenger and reference compound,^33^ respectively. A time-dependent decrease in the absorbance of DPBF (*λ*_max_ = 414 nm) was seen upon irradiation of the BODIPY arrays in CH_2_Cl_2_ solution, whereas no detectable decrease was seen in the dark (Fig. S56–S60†). The slope of the graph obtained by plotting the change in optical density against time was used to calculate the singlet oxygen quantum yield (Fig. 5). A value of *Φ*_Δ_ = 0.29 (eqn S1 in the ESI†) was obtained for 4·4BF_2_, which proved greater than the values for 4·2BF_2_ (0.05) and 4·3BF_2_ (0.05 and 0.19, respectively). This was taken as evidence of a facilitated ISC process in the case of 4·4BF_2_.

The redox properties of ligand 4 and its corresponding BODIPY arrays were also examined by cyclic voltammetry (CV) and differential pulse voltammetry (DPV) (Fig. S52–S55†). The resulting redox potentials are summarized in Table 1. All three BODIPY arrays display similar redox behaviour with the first oxidation waves being positively shifted by around 250 mV relative to the free ligand 4. In the case of 4·4BF_2_, the second reduction step is well separated from the first by 510 mV. This exceeds the splitting seen for 4·2BF_2_ (300 mV) and 4·3BF_2_ (240 mV). The larger splitting seen for 4·4BF_2_ is consistent with the high degree of electronic interaction between the BODIPY units and the relatively stronger exciton coupling inferred on the basis of the absorption spectral studies discussed above. We thus conclude that the structure, as well as degree of internal interaction, can be modified by conversion of 4 to the corresponding BF_2_-bound forms.

## Conclusions

In summary, we have optimized the synthesis of a bicyclic porphyrinoid 4 relative to a competing figure-of-eight monocyclic porphyrinoid product 3. This was done by controlling the stoichiometry and the acid-catalysed reaction conditions. A single-crystal X-ray diffraction based structural analyses revealed that ligand 4 interacts with THF and CH_3_OH in the solid state. It also detailed a structure that looked attractive as a starting material for the preparation of a series of covalently linked 3D BODIPY-like arrays. In fact, 4 could be converted readily to 4·2BF_2_, 4·3BF_2_, and 4·4BF_2_, albeit in modest yields. All three BODIPY arrays displayed similar solution state conformational dynamics as inferred from various spectroscopic studies. Nevertheless, they displayed well-defined solid state structures as inferred on the basis of single-crystal X-ray structural analyses. Of the three BODIPY-like species generated from 4, the tetrakis system, 4·4BF_2_, give rise to higher extinction coefficient (by *ca.* 2.5 times) than its bis- and tris-BF_2_ congeners, 4·2BF_2_ and 4·3BF_2_. Consistent with their conformationally dynamic features, all three BODIPY arrays proved only weakly fluorescent. However, evidence of intra-subunit coupling was inferred in at least the case of 4·4BF_2_. This leads us to suggest that further studies of 3D cryptand-like porphyrinoids is warranted both as potential receptors and as optical materials. Work along these lines is ongoing in our laboratories.

## Data availability

Crystallographic data for 4, 4·2BF_2_, 4·3BF_3_ and 4·4BF_2_ have been deposited at the CCDC under accession numbers 2110148 and 2150674–2150676, respectively, and can be obtained from the Cambridge Crystallographic Data Centre (CCDC). Data supporting this report, including cyclic voltammetry, singlet oxygen measurement, NMR and MS spectra, absorption and emission spectra, DFT calculation details and the calculated Cartesian coordinates of various complexes, have been uploaded as part of the ESI.†

## Author contributions

Project conceptualization and research supervision: J. L. S., T. S. and C. L.; synthesis, characterization, NMR, spectroscopy, CV and DPV studies: W. N. and L. Y.; single crystal generation and data analysis: W. N.; theoretical calculations: C. L.; writing–original draft: W. N.; writing–review and editing: J. L. S., C. L. and W. N. All authors proofread, commented on, and approved the final version of this manuscript.

## Conflicts of interest

There are no conflicts to declare.

## Supplementary Material

SC-013-D2SC01635D-s001

SC-013-D2SC01635D-s002
