# Supplementary material for: Controlled assembly of a bicyclic porphyrinoid and its 3-dimensional boron difluoride arrays
Source: Chem Sci. 2022 May 26;13(24):7276–82. doi: 10.1039/d2sc01635d (PMC9214847; doi:10.1039/d2sc01635d)
Supplement: SC-013-D2SC01635D-s001 [file SC-013-D2SC01635D-s001.pdf]

Electronic Supplementary Material (ESI) for Chemical Science.
This journal is © The Royal Society of Chemistry 2022

Supporting Information for:

Controlled assembly of a bicyclic porphyrinoid and Its 3-dimensional boron difluoride arrays

Weinan Zhou, Tridib Sarma, Liu Yang, Chuanhu Lei, and Jonathan L. Sessler

Table of Content

1. General information 3

2. Synthetic procedures and characterization data 4

3. NMR and MS spectra 9

4. Crystal data 26

5. Optical and electrochemical studies 32

6. Singlet oxygen measurements 38

7. DFT calculations 40

8. Supporting references 47

1. General information

All reagents and solvents were of commercial reagent grade and were used without further purification except where noted. Dry CH_2Cl_2 was obtained by refluxing and distillation over CaH_2. Silica gel column chromatography was performed on Qingdao Haiyang silica gel (200-300 mesh). Alumina column chromatography was performed on Merck deactivated Brockmann III neutral alumina oxide. Thin-layer chromatography (TLC) was carried out on pre-coated, glass-backed silica gel plates. All NMR spectra were acquired on either a Bruker 600 MHz instrument or a JEOL ECZ 400 MHz NMR spectrometer. Chemical shifts are reported in ppm using residual solvent signals as the internal reference standards. ^{1}H NMR signals were assigned from ^{1}H-^{1}H COSY and 2D ROESY experiments. Spectroscopic solvents were purchased from Cambridge Isotope Laboratories. Inc. Mass spectra (MS) were taken on Bruker (Autoflex speed) matrix-assisted laser desorption ionization time-of-flight mass spectrometry (MALDI-TOF MS). Optical absorption spectra were recorded on a Varian Cary 5000 spectrophotometer. Fluorescence spectra are recorded on a steady-state Shimadzu RF6000 fluorimeter and Edinburgh Instruments FS5 spectrofluorometer. Fluorescence decay and absolute quantum yields were recorded at room temperature with an Edinburgh Instruments FLS1000 spectrofluorometer. Cyclic voltammetric (CV) and differential pulse voltammetric (DPV) studies were carried out on a CHI660E Instrument with an electrochemical system involving a three-electrode configuration consisting of a glassy carbon (working electrode), platinum wire (counter electrode) and Ag/Ag^+ (reference electrode) in dry dichloromethane with n-tetrabutylammonium hexafluorophosphate ($TBAPF_6$, 0.1 M) as the supporting electrolyte. All the solutions were degassed by means of sonication prior to their used in the electrochemical studies.

Single crystal X-ray diffraction analyses were performed on a Bruker SMART APEX diffractometer equipped with a CCD area detector using a graphite monochromator with CuK_α (λ = 1.54187 Å) radiation. The structures were solved by direct methods, followed by full-matrix least-squares refinement against F^2 using the SHELXS and SHELXL programs in the Olex2 package.[1] The crystal data has been deposited in the Cambridge Crystallographic Data Centre with reference nos. CCDC 2110148 [**4**] CCDC 2150674 [**4·2BF$_2$**] CCDC 2150675 [**4·4BF$_2$**] and CCDC 2150676 [**4·3BF$_2$**]. Crystallographic data are also listed in Tables **S2** and **S3**.

2. Synthetic procedures and characterization data

Compounds **1**[2] and **2**[3]were prepared according to previously reported procedures.

Table S1. Optimization of the reaction conditions for **3** and **4**.

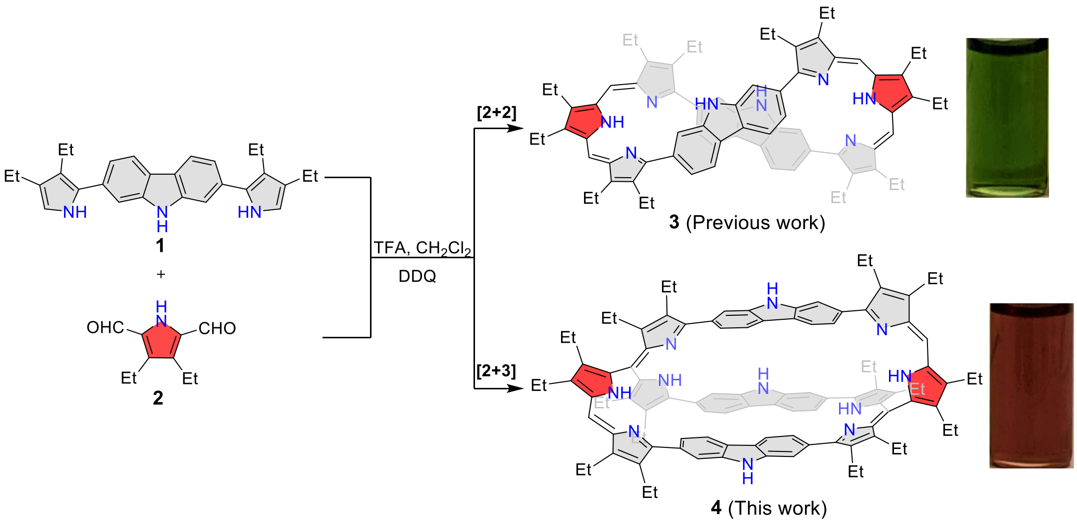

entry	Variation from the initial condition[a]	yield (%)	
		**3**	**4**
1	none	45	trace
2	TFA (5 equiv.)	58	trace
3	TFA (10 equiv.)	82	none
4	TFA (1 equiv.)	none	32
5	TFA (1 equiv.), DDQ (4 equiv.)	none	47
6	TFA (1 equiv.), DDQ (5 equiv.)	none	55
7	TFA (1 equiv.), DDQ (6 equiv.)	none	49
8	TFA (1 equiv.), DDQ (5 equiv.), **1** (2 equiv.)	none	79
9	TFA (1 equiv.), DDQ (5 equiv.), **1** (2 equiv.), 6 h	none	72
10	TFA (1 equiv.), DDQ (5 equiv.), **1** (2 equiv.), 2 h	none	62
11	TFA (1 equiv.), DDQ (5 equiv.), **1** (2.5 equiv.)	none	75

[a] Reaction conditions: **1** (0.28 mmol), **2** (0.28 mmol), TFA (54 µL, 2.5 equiv.), DDQ (0.84 mmol, 3 equiv), CH_2Cl_2 (500 mL, 0.55 mM), 4 h and then stirred for 40 min. after adding DDQ; room temperature. Yields are isolated yields. The term "none" refers to a level below what could be detected by TLC analysis.

Synthesis of BODIPY arrays

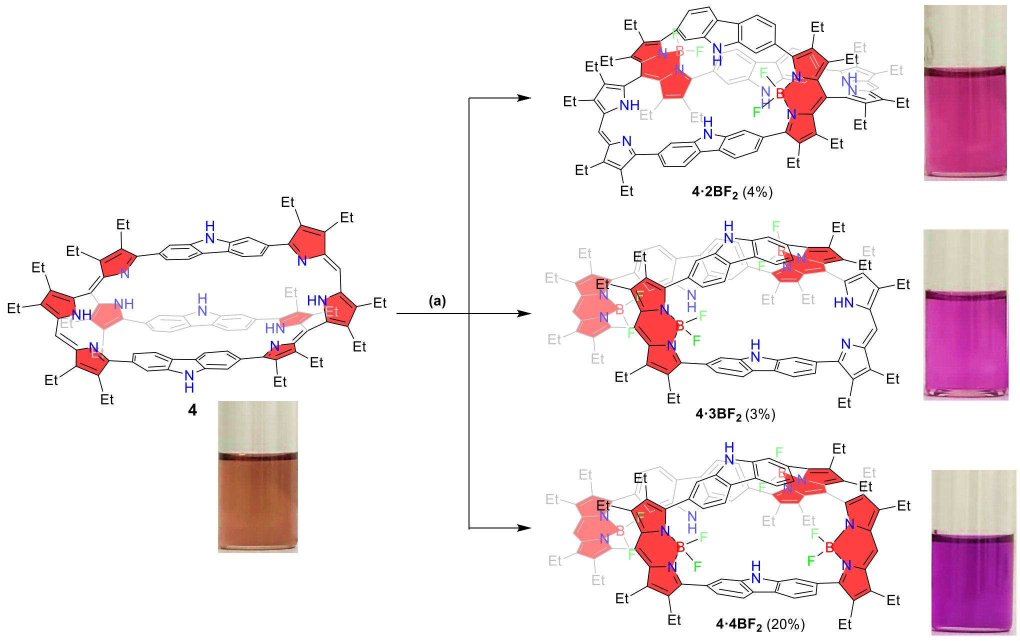

Scheme S1. Reagents and conditions: (a) $Et_2O{\cdot}BF_3$, TEA, 110 °C.

Optimized synthesis of cryptand 4

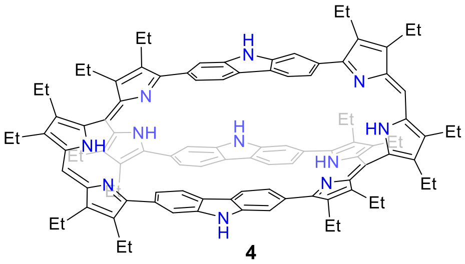

4

A 1 L two-necked round bottom flask equipped with a magnetic stirring bar, an N_2 inlet and a septum was charged with compounds **1** (228 mg, 0.56 mmol) and **2** (50 mg, 0.28 mmol). Freshly distilled CH_2Cl_2 (500 mL) was then added via a cannula under an inert atmosphere. The reaction mixture was allowed to mix well and stirred for 10 min. at room temperature. Trifluoroacetic acid (21 µL, 0.28 mmol) was then added via a micro syringe and the resultant mixture was allowed to stir at room temperature in the absence of light for 4 h. 2,3-Dichloro-5,6-dicyano-1,4-benzoquinone, DDQ (317 mg, 1.4 mmol) was then added slowly, and the mixture was stirred for a further 40 min. open to the air. After removal of the volatiles in a rotary evaporator, the residual was purified over a neutral alumina column using a mixture of CH_2Cl_2/*n*-hexane as the eluent. The dark reddish-brown coloured fraction was collected and evaporated to dryness to give the desired product **4**. Recrystallization from CH_2Cl_2/methanol afforded compound **4** in the form of a dark black solid in 79% yield.

Analytical data for 4: ^{1}H NMR (400 MHz, THF-d_8, 25 °C, δ in ppm) δ 15.69 (s, 2H), 10.24 (s, 2H), 10.12 (s, 1H), 8.38 (d, J = 8.2 Hz, 2H), 7.80 (d, J = 8.1 Hz, 2H), 7.70 (s, 2H), 7.60 (s, 2H), 7.46 (d, J =

8.4 Hz, 2H), 7.39 (d, J = 8.0 Hz, 2H), 7.37 (s, 2H), 7.14 (d, J = 8.3 Hz, 2H), 6.99 (d, J = 8.2 Hz, 2H), 6.97 (s, 2H), 3.04 – 2.98 (m, 2H), 2.84 – 2.63 (m, 20H), 2.47 – 2.38 (m, 10H), 2.26 – 2.16 (m, 4H), 1.41 (t, J = 7.6 Hz, 6H), 1.35 – 1.28 (m, 12H), 1.22 (m, 12H), 1.08 (t, J = 7.4 Hz, 6H), 1.02 (t, J = 7.4 Hz, 6H), 0.67 (t, J = 7.3 Hz, 6H).

^{1}H NMR (400 MHz, THF-d_8, -90 °C, aromatic region, δ in ppm) δ 15.74 (s, 2H), 13.19 (s, 2H), 11.01 (s, 1H), 10.84 (s, 2H), 8.50 (d, J = 7.9 Hz, 2H), 7.85 (d, J = 8.0 Hz, 2H), 7.71 (s, 2H), 7.62 (s, 2H), 7.49 (d, J = 8.3 Hz, 2H), 7.45 (s, 2H), 7.40 (d, J = 7.9 Hz, 2H), 7.15 (d, J = 6.8 Hz, 4H), 6.99 (d, J = 8.0 Hz, 2H).

MS (MALDI-TOF) *m/z*: Calcd for $C_{104}H_{107}N_{11}$+H^+ = 1511.8818 [M+H]$^+$; found = 1511.5491.

UV-Vis (toluene): λ_{max} [nm] (ε [M^{-1} cm^{-1}] x10^5) = 355 (0.86), 500 (0.66), 625 (0.25).

Synthesis of BODIPY arrays

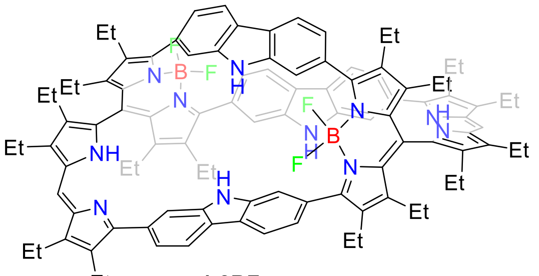

4·2BF$_2$

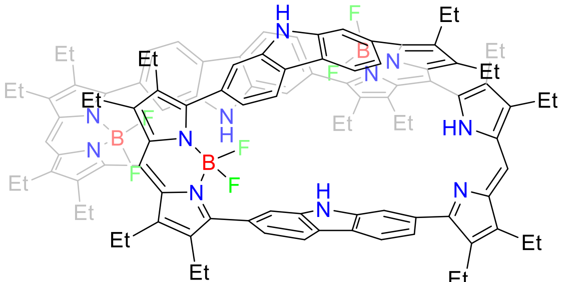

4·3BF$_2$

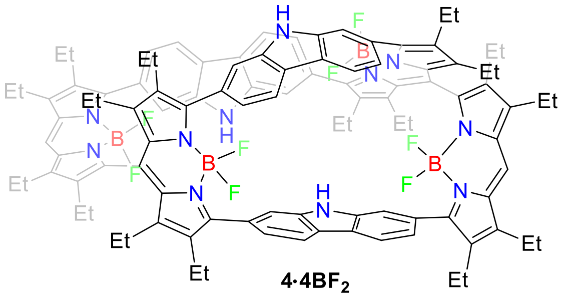

4·4BF$_2$

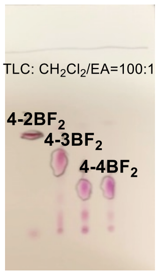

A 100 mL round bottom flask equipped with a condenser containing **4** (40 mg, 26.5 μmol) and a stirring bar was charged with 40 mL dry toluene under an inert atmosphere. The macrocycle was allowed to dissolve, and the reaction mixture stirred for 10 min. at 110 °C. Triethylamine (0.36 mL, 2.65 mmol) was then added to the solution, which was stirred for 15 minutes. BF_3·OEt_2 (0.39 mL, 3.18 mmol) was then added dropwise with constant stirring. The reaction mixture was further stirred for 8 h at 110 °C under an inert atmosphere. This procedure was repeated 5 times in order to obtain enough material to permit characterization. The volatiles were then removed using a rotary evaporator and the resulting residue purified *via* column chromatography on silica gel as detailed below.

Initially, the column was packed in petroleum ether (PE) and a silica slurry of the reaction mixture was

loaded on to the column. The initial eluent consisted of 10% dichloromethane in petroleum ether (PE). Carefully increasing the polarity up to 40% (dichloromethane in PE) caused compound **4·2BF$_2$** to elute as the main red coloured fraction. Evaporation and further recrystallization from a mixture of CH_2Cl_2/methanol gave 10 mg of **4·2BF$_2$** (4%) as a crystalline solid with a green metallic lustre. Further, increasing the polarity up to 50% caused complex **4·3BF$_2$** to elute as a purple-coloured fraction. Evaporation of the volatiles followed by recrystallization from CH_2Cl_2/methanol yielded 7.5 mg (3%) of **4·3BF$_2$** as a crystalline solid with a green metallic lustre. With no change in the eluent polarity **4·4BF$_2$** was subsequently seen to elute as a violet fraction. Evaporation of the volatiles followed by recrystallization from CH_2Cl_2/methanol yielded 50 mg (20%) of **4·4BF$_2$** as a crystalline solid with a golden metallic lustre.

Analytical data for 4·2BF$_2$: ^{1}H NMR (400 MHz, THF-d_8, 25 °C, δ in ppm) δ 10.44 (s, 1H), 8.93 (br, 1H), 8.07 (d, J = 8.1 Hz, 2H), 8.03 (d, J = 8.1 Hz, 2H), 7.79 (d, J = 8.1 Hz, 2H), 7.62 (d, J = 8.2 Hz, 2H), 7.47 (s, 2H), 7.42 (s, 2H), 7.17 (d, J = 8.1 Hz, 2H), 7.10 (s, 2H), 7.04 (d, J = 8.1 Hz, 2H), 2.85 – 2.67 (m, 22H), 2.35 – 2.06 (m, 10H), 1.33 (t, J = 7.5 Hz, 6H), 1.25 (q, J = 7.1 Hz, 18H), 1.11 (t, J = 7.4 Hz, 6H), 0.88 (q, J = 6.6 Hz, 12H), 0.76 (t, J = 7.4 Hz, 6H).

^{1}H NMR (400 MHz, THF-d_8, aromatic region, -80 °C, δ in ppm) δ 11.68 (s, 1H), 10.97 (s, 1H), 10.89 (s, 1H), 10.62 (s, 1H), 8.16 (d, J = 7.7 Hz, 1H), 8.08 (d, J = 7.8 Hz, 1H), 8.04 (d, J = 7.6 Hz, 1H), 7.95 (d, J = 7.8 Hz, 1H), 7.83 (dd, J = 11.0, 7.1 Hz, 2H), 7.61 (d, J = 7.8 Hz, 1H), 7.43 (d, J = 7.9 Hz, 1H), 7.39 – 7.19 (m, 8H), 7.15 (d, J = 5.0 Hz, 2H), 6.75 (d, J = 7.8 Hz, 2H).

^{19}F NMR (376 MHz, THF-d_8, -80 °C, δ in ppm) δ -118.03, -118.59, -144.38, -149.25.

^{11}B NMR (128 MHz, THF-d_8, 25 °C, δ in ppm) δ -0.32.

MS (MALDI-TOF) *m/z*: Calcd for $C_{104}H_{105}B_2F_4N_{11}$-$F^+$ = 1587.8726 [M-F]$^+$; found = 1587.493.

UV-Vis (toluene): λ_{abs} [nm] (ε [M^{-1} cm^{-1}] x10^5) = 334 (0.72), 539 (0.87); Fluorescence emission (toluene): λ_{em} [nm] = 640.

Analytical data for 4·3BF$_2$: ^{1}H NMR (600 MHz, THF-d_8, 25 °C, δ in ppm) δ 10.00 (s, 1H), 9.93 (s, 1H), 8.00 (t, J = 8.7 Hz, 2H), 7.91 (d, J = 7.8 Hz, 1H), 7.67 (s, 1H), 7.66 – 7.61 (m, 2H), 7.44 (d, J = 12.6 Hz, 1H), 7.30 (s, 1H), 7.16 (t, J = 7.8 Hz, 1H), 7.04 (s, 1H), 2.88 (dd, J = 7.6, 3.5 Hz, 2H), 2.85 – 2.80 (m, 4H), 2.75 – 2.65 (m, 12H), 2.48 – 2.43 (m, 8H), 2.32 – 2.28 (m, 4H), 2.23 – 2.19 (m, 2H), 1.34 – 1.19 (m, 27H), 1.04 – 0.92 (m, 12H), 0.83 – 0.80 (m, 6H), 0.76 (t, J = 7.4 Hz, 3H).

^{1}H NMR (400 MHz, THF-d_8, aromatic region, -60 °C, δ in ppm) δ 10.96 (s, 1H), 10.27 (s, 1H), 9.97 (s,

1H), 8.10 (d, *J* = 7.5 Hz, 1H), 8.04 (d, *J* = 7.8 Hz, 1H), 7.99 – 7.95 (m, 2H), 7.91 (s, 1H), 7.88 – 7.82 (m, 2H), 7.69 (d, *J* = 8.1 Hz, 1H), 7.62 (d, *J* = 9.0 Hz, 1H), 7.49 (d, *J* = 8.1 Hz, 1H), 7.38 (s, 1H), 7.30 (s, 1H), 7.23 – 7.10 (m, 6H), 6.73 (d, *J* = 8.8 Hz, 1H), 6.56 (s, 1H).

^{19}F NMR (376 MHz, THF-d_8, -80 °C, δ in ppm) δ -109.87, -118.15, -122.58, -146.84, -149.99, -151.50.

^{11}B NMR (128 MHz, THF-d_8, 25 °C, δ in ppm) δ -1.06, -0.36.

MS (MALDI-TOF) *m/z*: Calcd for $C_{104}H_{104}B_3F_6N_{11}$-$F^+$ = 1634.8675 $[M-F]^+$; found = 1634.475.

UV-Vis (toluene): λ_{abs} [nm] (ε [M^{-1} cm^{-1}] x10^5) = 330 (0.49), 400 (0.31), 548 (0.98); Fluorescence emission (toluene): λ_{em} [nm] = 660.

Analytical data for 4·4BF₂: ^{1}H NMR (400 MHz, THF-d_8, 25 °C, δ in ppm) δ 10.14 (s, 2H), 9.58 (s, 1H), 7.95 (t, *J* = 8.2 Hz, 4H), 7.77 (s, 2H), 7.63 (d, *J* = 3.4 Hz, 2H), 7.58 (d, *J* = 8.1 Hz, 2H), 7.40 (d, *J* = 7.8 Hz, 2H), 7.36 – 7.29 (m, 2H), 7.15 (d, *J* = 8.1 Hz, 2H), 7.04 (s, 1H), 2.97 – 2.81 (m, 8H), 2.75 –2.69 (m, 8H), 2.62 – 2.58 (m, 8H), 2.36 – 2.29 (m, 4H), 2.18 – 1.90 (m, 4H), 1.37 – 1.32 (q, *J* = 7.6 Hz, 12H), 1.28– 1.24 (t, *J* = 7.6 Hz, 6H), 1.02 – 0.96 (m, 18H), 0.81 – 0.77 (m, 12H).

^{1}H NMR (400 MHz, THF-d_8, aromatic region, -40 °C, δ in ppm) δ 10.82 (s, 1H), 10.19 (s, 1H), 9.75 (s, 1H), 8.10 (d, *J* = 8.0 Hz, 2H), 8.04 (d, *J* = 7.9 Hz, 1H), 7.93 (d, *J* = 8.0 Hz, 1H), 7.85 (s, 2H), 7.83 (d, *J* = 7.8 Hz, 1H), 7.78 – 7.76 (m, 2H), 7.66 (d, *J* = 8.0 Hz, 1H), 7.59 (d, *J* = 7.8 Hz, 1H), 7.49 (d, *J* = 8.2 Hz, 1H), 7.37 (s, 1H), 7.30 (s, 1H), 7.22 – 7.22 (m, 4H), 6.86 (d, *J* = 8.4 Hz, 1H), 6.57 (s, 1H).

^{19}F NMR (376 MHz, THF-d_8, -80 °C, δ in ppm) δ -108.93, -117.59, -122.05, -122.87, -147.58, -148.28, -150.16, -151.34.

^{11}B NMR (128 MHz, THF-d_8, 25 °C, δ in ppm) δ -0.28, -0.65.

MS (MALDI-TOF) *m/z*: Calcd for $C_{104}H_{103}B_4F_8N_{11}$ = 1701.8712 $[M]^+$; found = 1701.438.

UV-Vis (toluene): λ_{abs} [nm] (ε [M^{-1} cm^{-1}] x10^5) = 330 (0.78), 400 (0.63), 551 (2.27); Fluorescence emission (toluene): λ_{em} [nm] = 664.

3. NMR and MS spectra

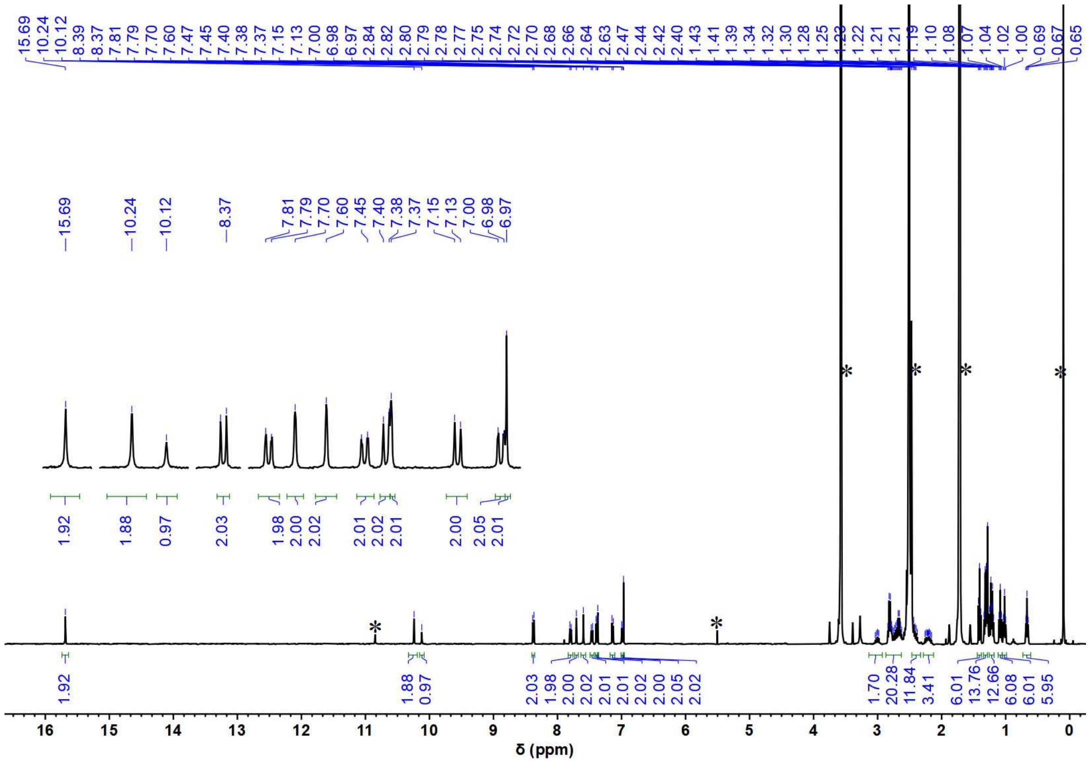

Figure S1. ^{1}H NMR spectrum of **4** recorded at 25 °C in THF-d_8. *Asterisk indicates residual solvent impurities.

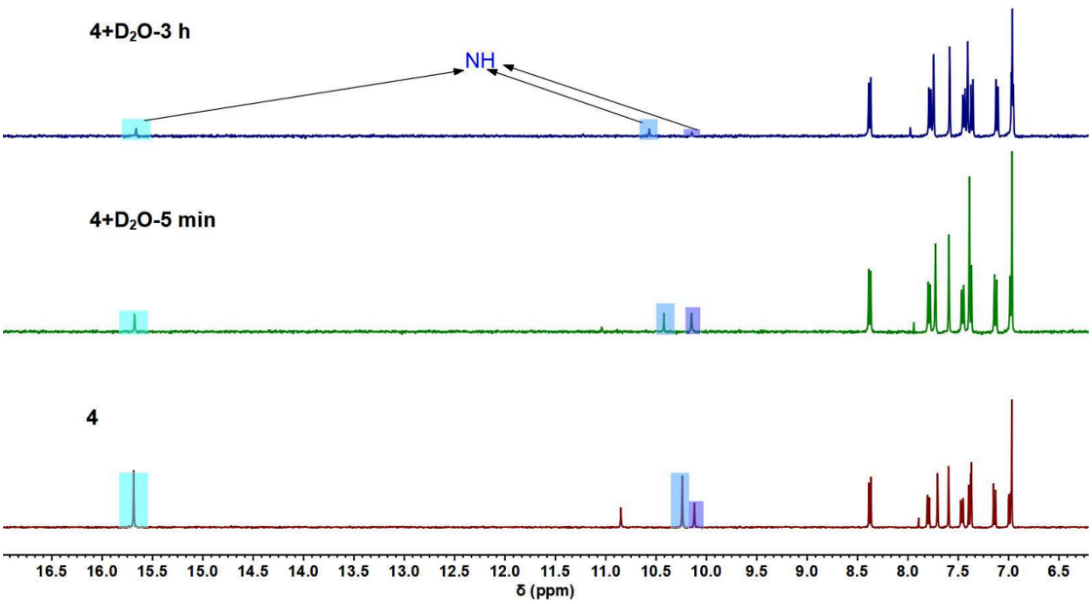

Figure S2. Comparative ^{1}H NMR spectrum of **4** after the addition of D_2O recorded at 25 °C in THF-d_8.

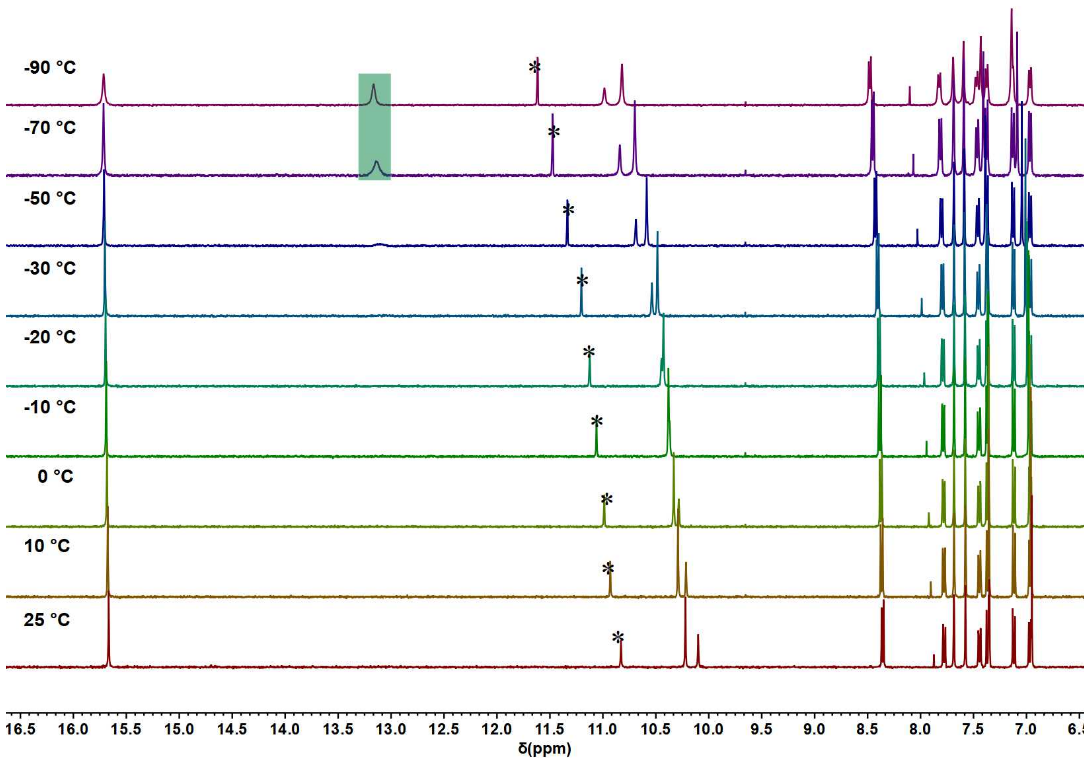

Figure S3. VT ^{1}H NMR spectra (aromatic region) of **4** recorded in THF-d_8. *Asterisks indicate residual solvents and impurities.

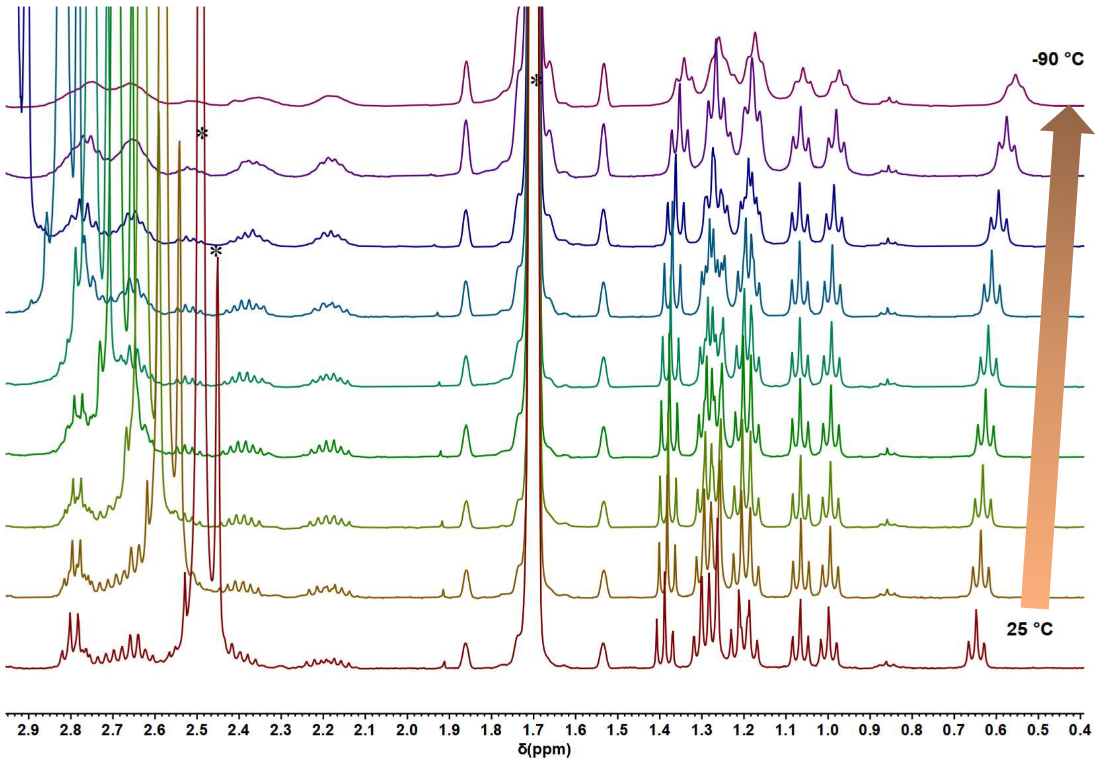

Figure S4. V.T. ^{1}H-NMR spectra (alkyl region) of **4** recorded in THF-d_8. *Asterisks indicate residual solvents and impurities.

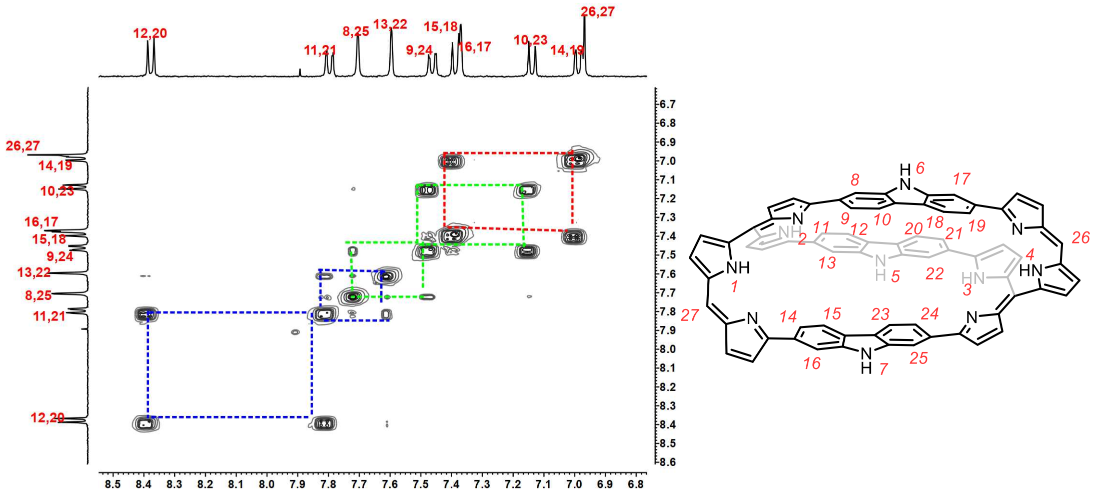

Figure S5. ^{1}H-^{1}H COSY spectrum of **4** (aromatic region) recorded at 25 °C in THF-d_8. COSY correlations are highlighted by dashed squares or with double arrows.

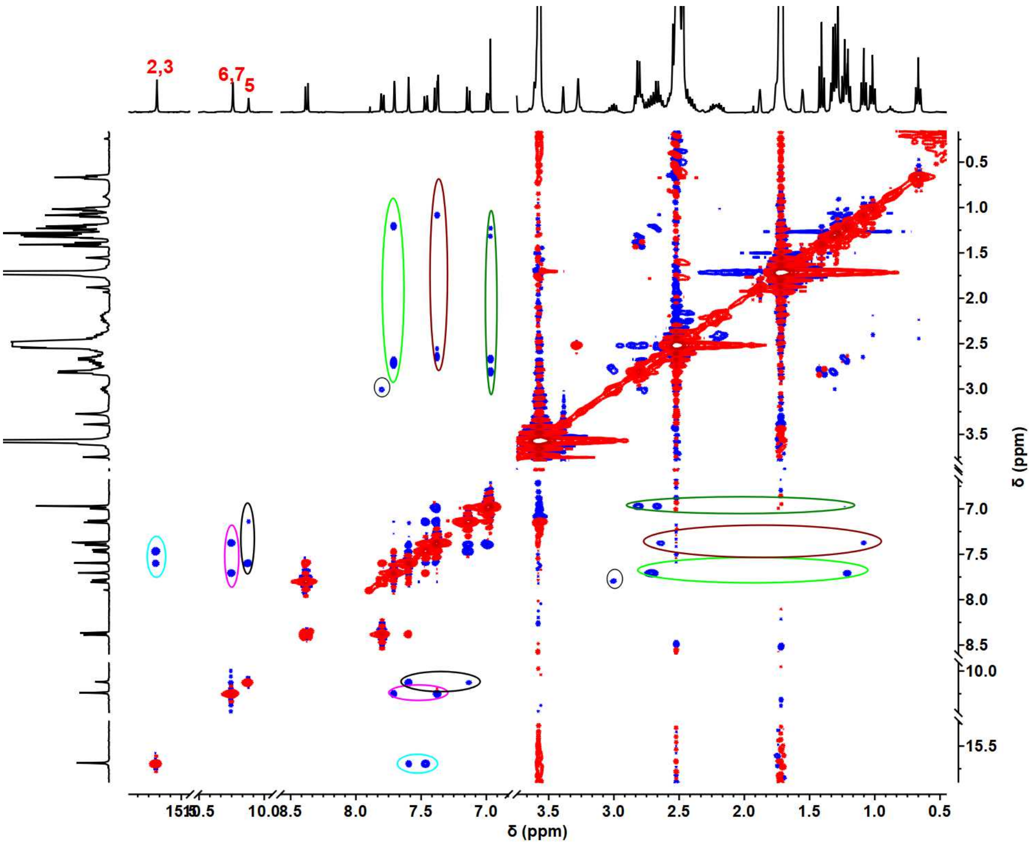

Figure S6. ROESY spectrum of **4** recorded at 25 °C in THF-d_8. Strong NOE effects are highlighted with ellipsoids.

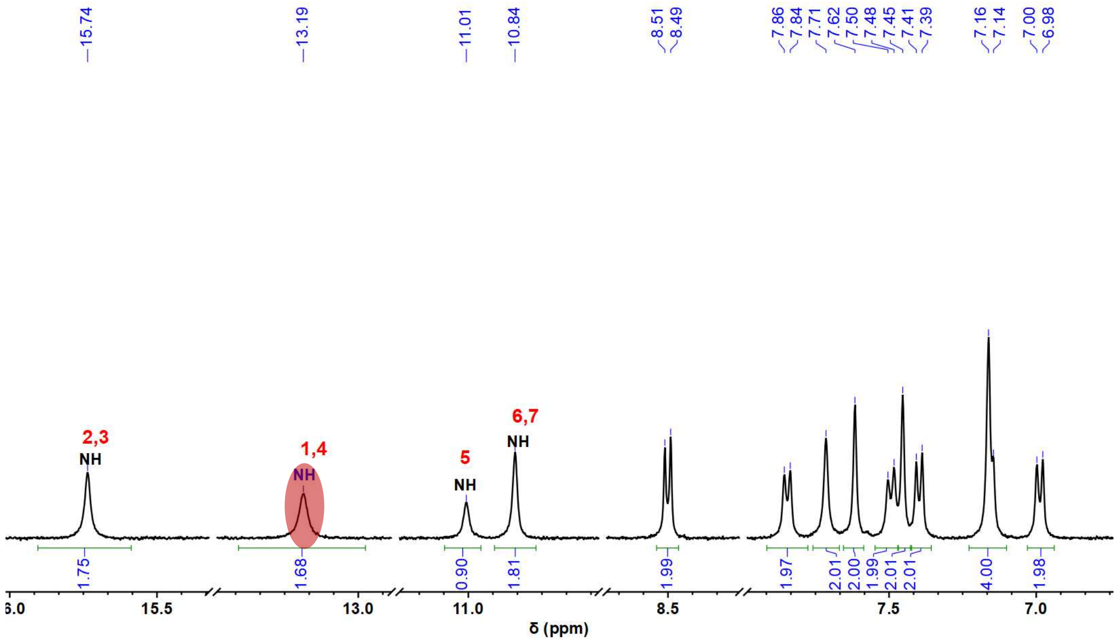

Figure S7. ^{1}H NMR spectrum of **4** recorded at -90 °C in THF-d_8.

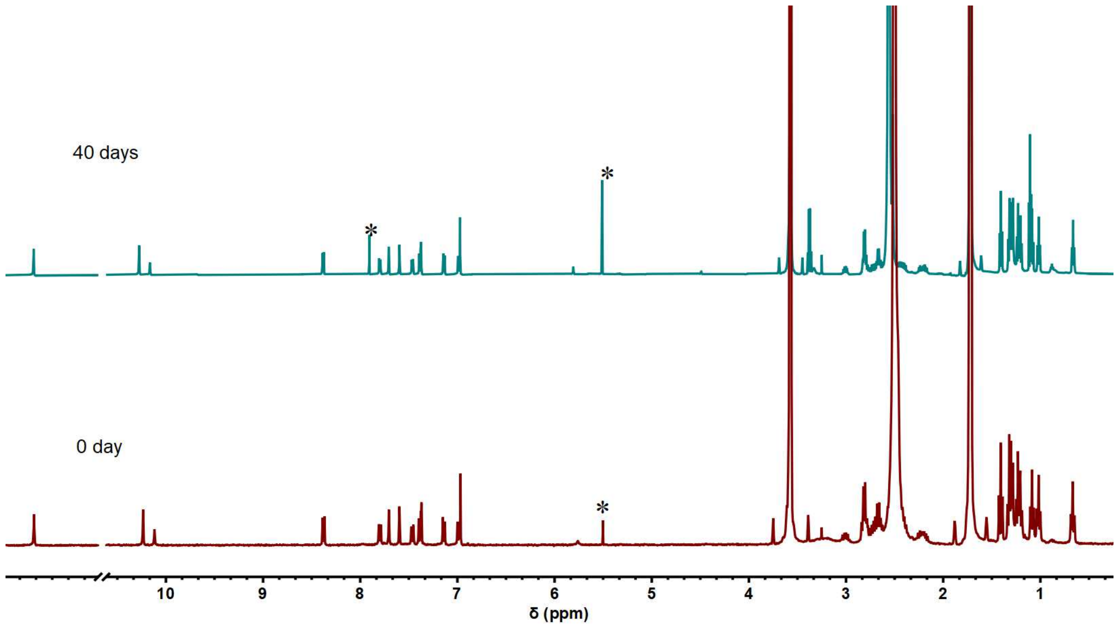

Figure S8. Comparative ^{1}H NMR spectrum of **4** recorded at 25 °C in THF-d_8 before and after being kept in a 2~8 °C refrigerator for 40 days. *Asterisks indicate residual solvents and impurities.

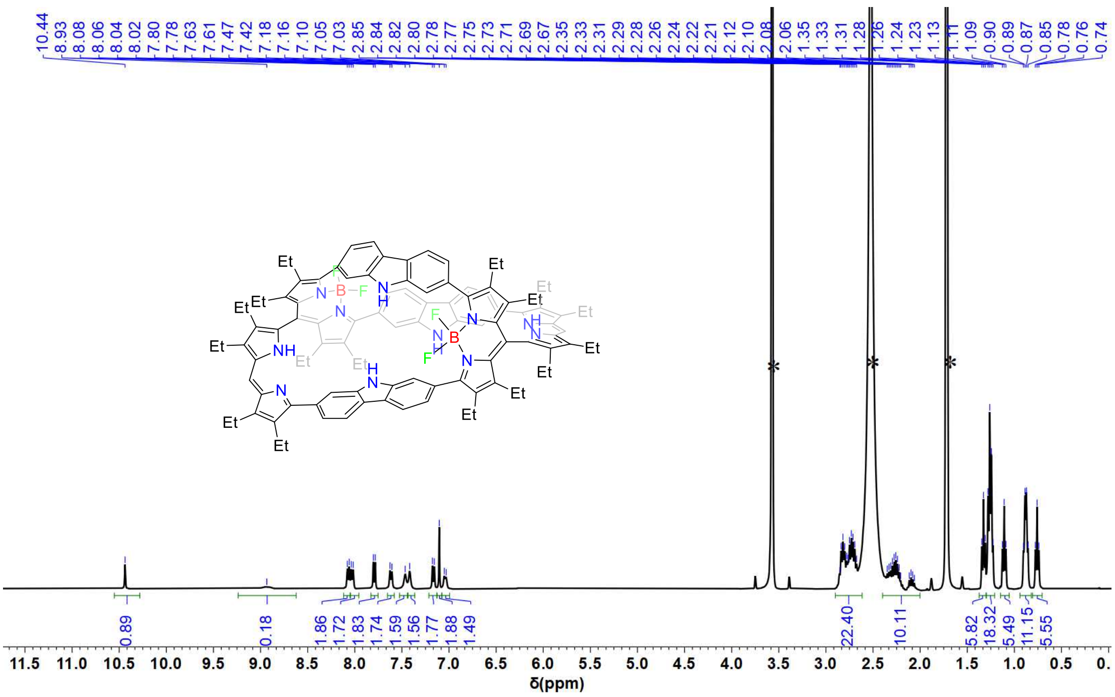

Figure S9. ^{1}H NMR spectrum of **4·2BF₂** recorded at 25 °C in THF-d_8. *Asterisks indicate residual solvent impurities.

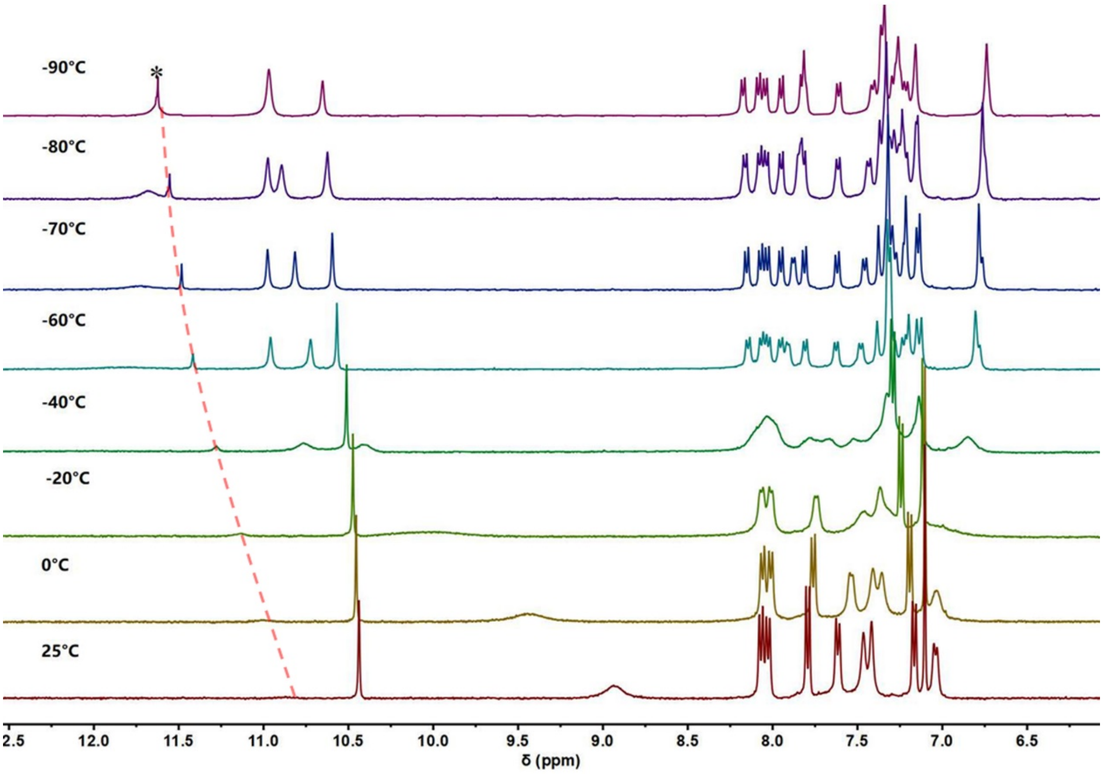

Figure S10. V.T. ^{1}H NMR spectra (aromatic region) of **4·2BF₂** recorded in THF-d_8. *Asterisks indicate residual solvent impurities.

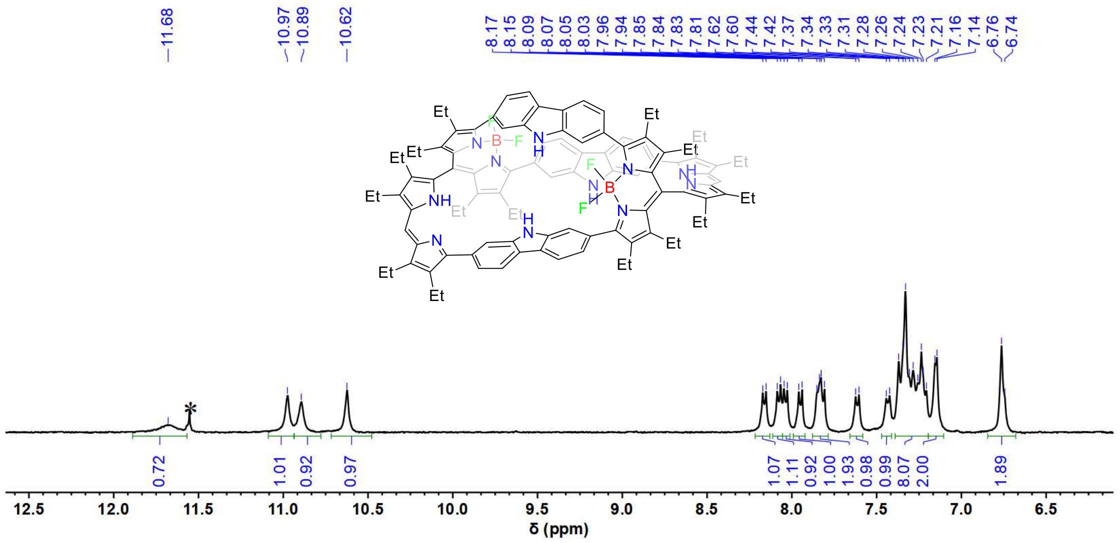

Figure S11. ^{1}H NMR spectra (aromatic region) of **4·2BF₂** recorded at -80 °C in THF-d_8.

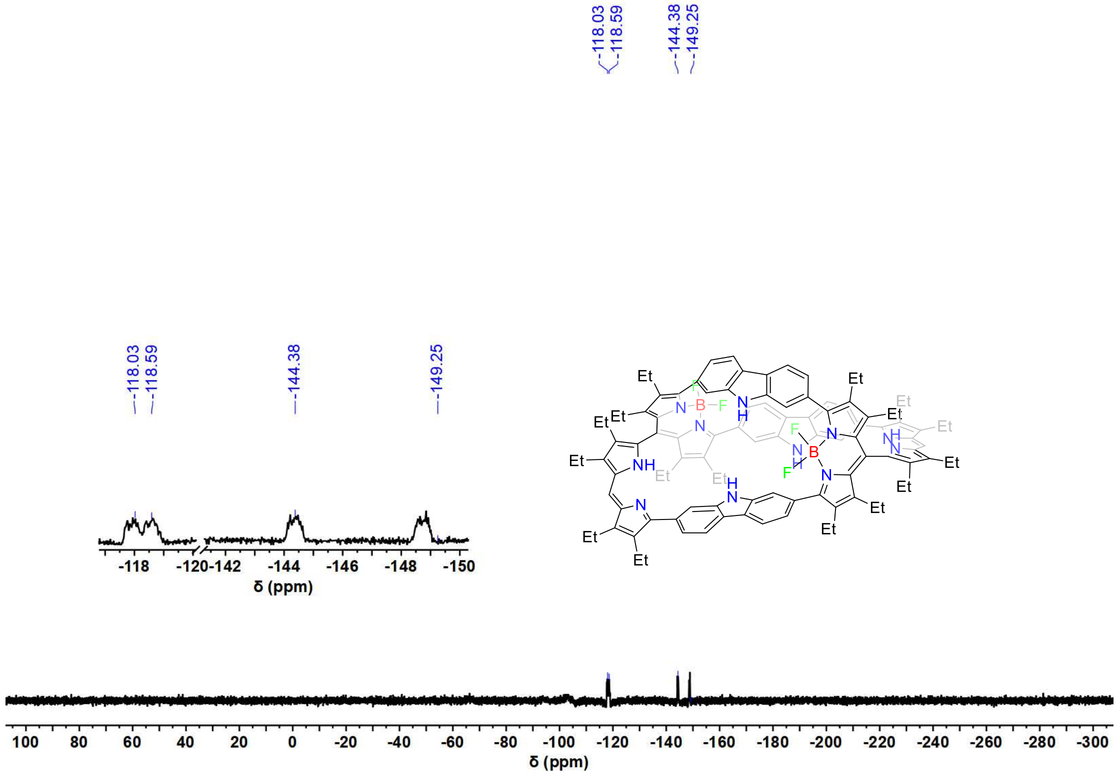

Figure S12. ^{19}F NMR spectrum of **4·2BF₂** recorded at -80 °C in THF-d_8.

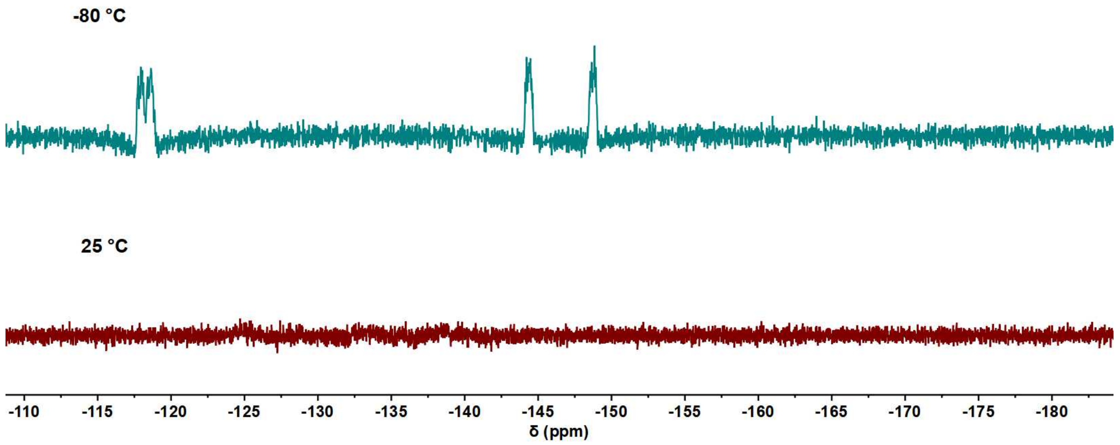

Figure S13. Comparison between ^{19}F NMR spectra of **4·2BF₂** recorded at two different temperatures in THF-d_8.

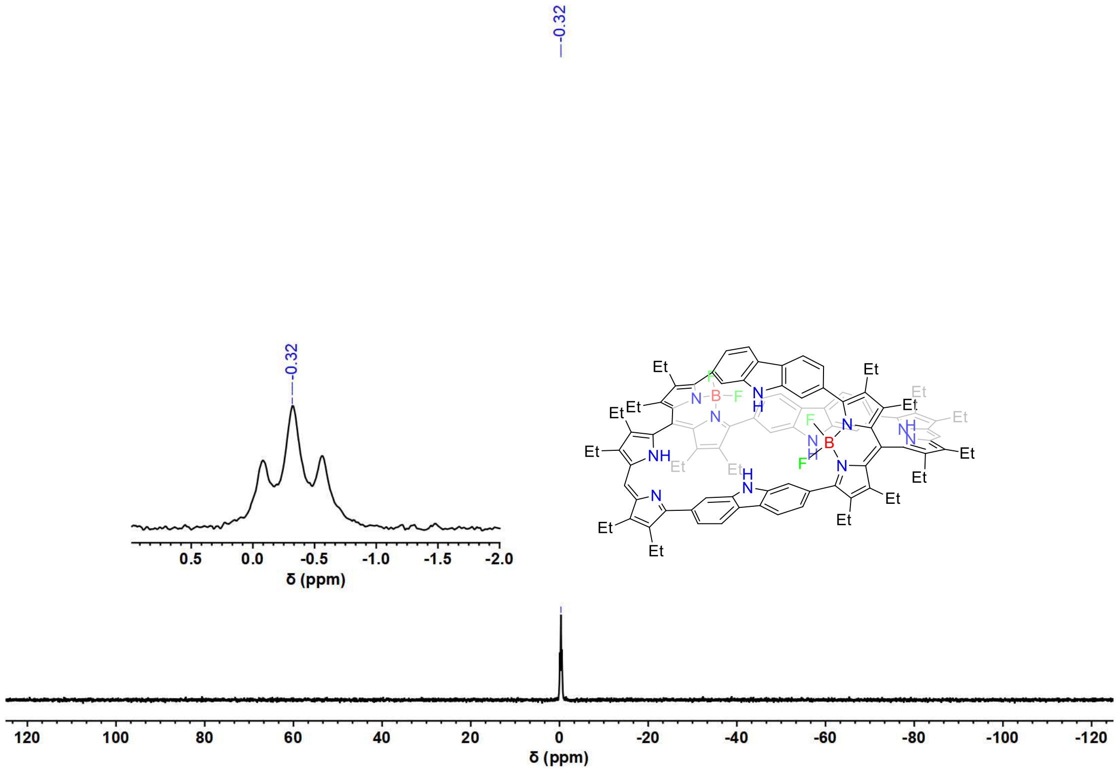

Figure S14. ^{11}B NMR spectrum of **4·2BF₂** recorded at 25 °C in THF-d_8.

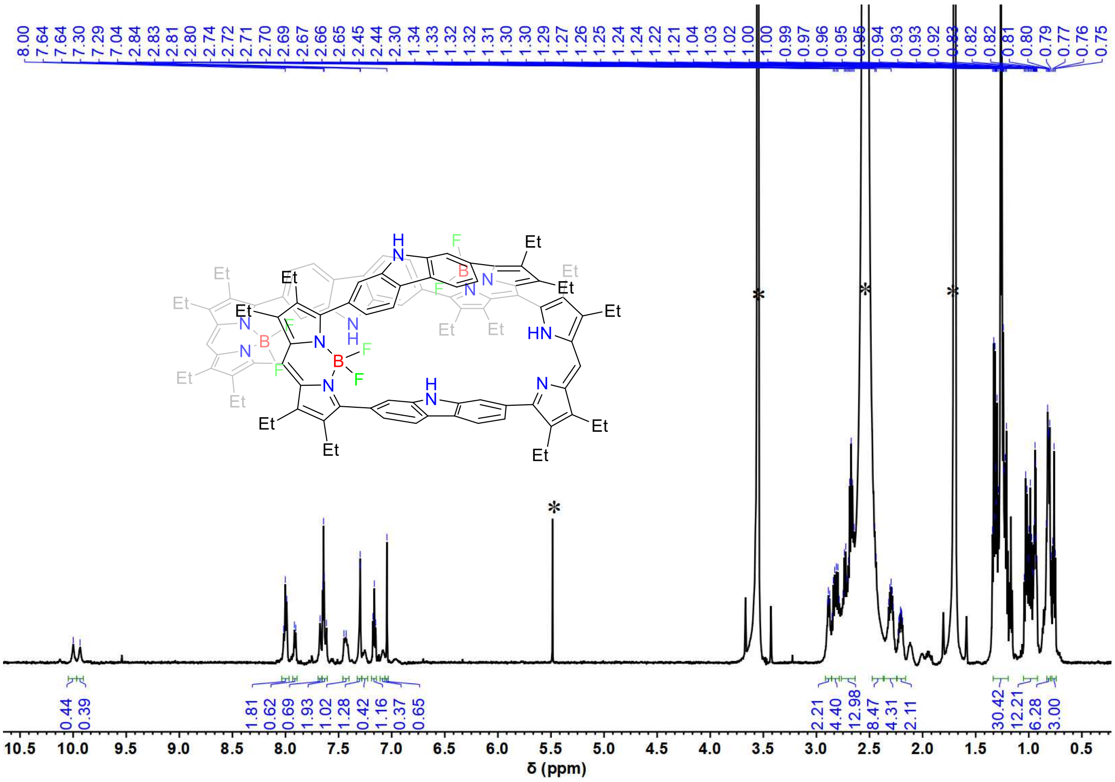

Figure S15. ^{1}H NMR spectrum of **4·3BF₂** recorded at 25 °C in THF-*d*8. *Asterisks indicate residual solvent impurities.

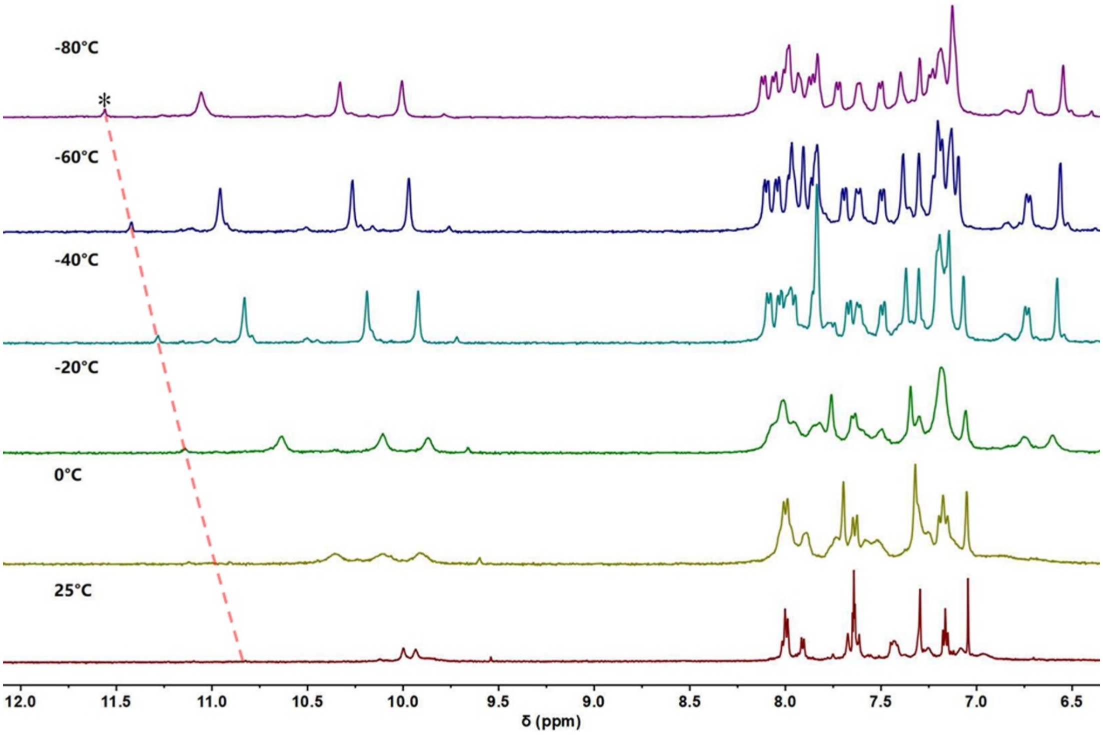

Figure S16. VT ^{1}H-NMR spectra (aromatic region) of **4·3BF₂** recorded in THF-*d*8. *Asterisks indicate represent residual solvent impurities.

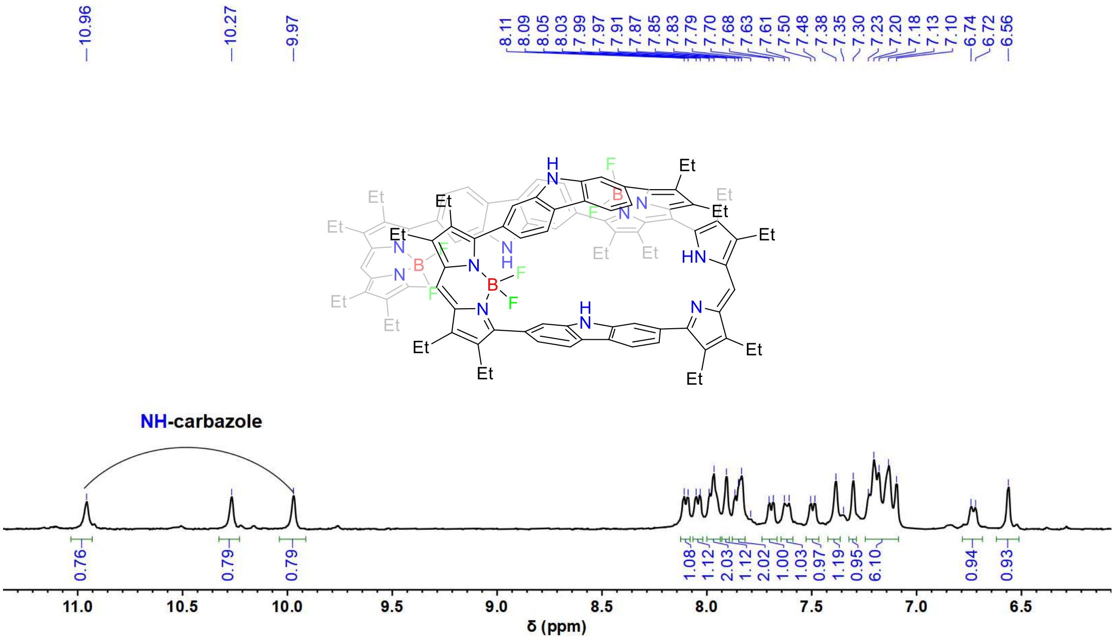

Figure S17 ^{1}H NMR spectrum (aromatic region) of **4·3BF₂** recorded at -60 °C in THF-*d*₈.

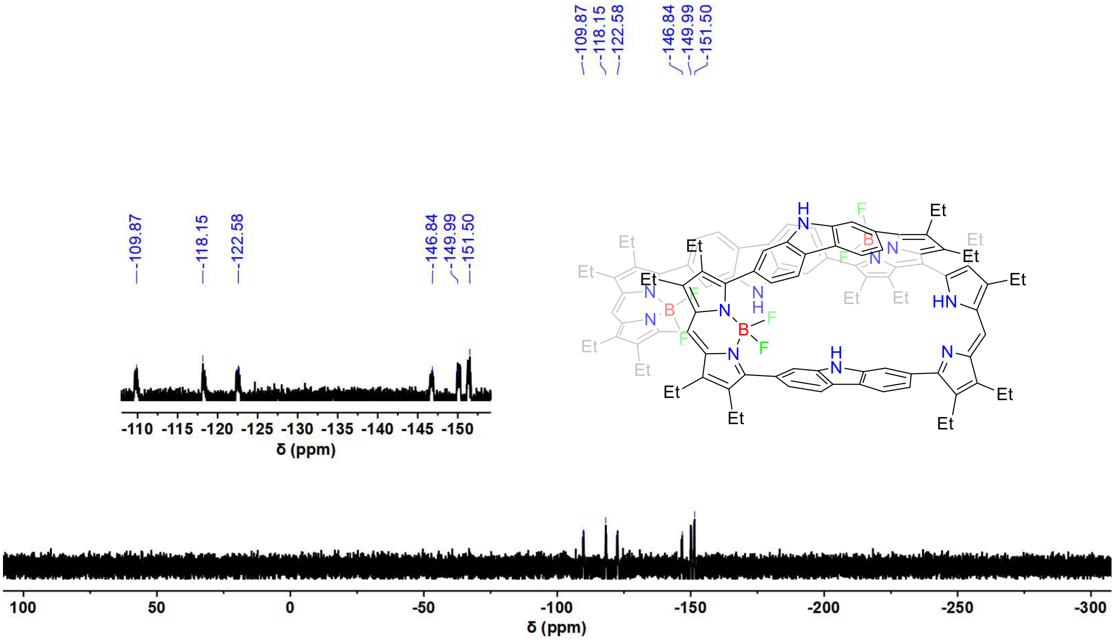

Figure S18. ^{19}F NMR spectrum of **4·3BF₂** recorded at -80 °C in THF-*d*₈.

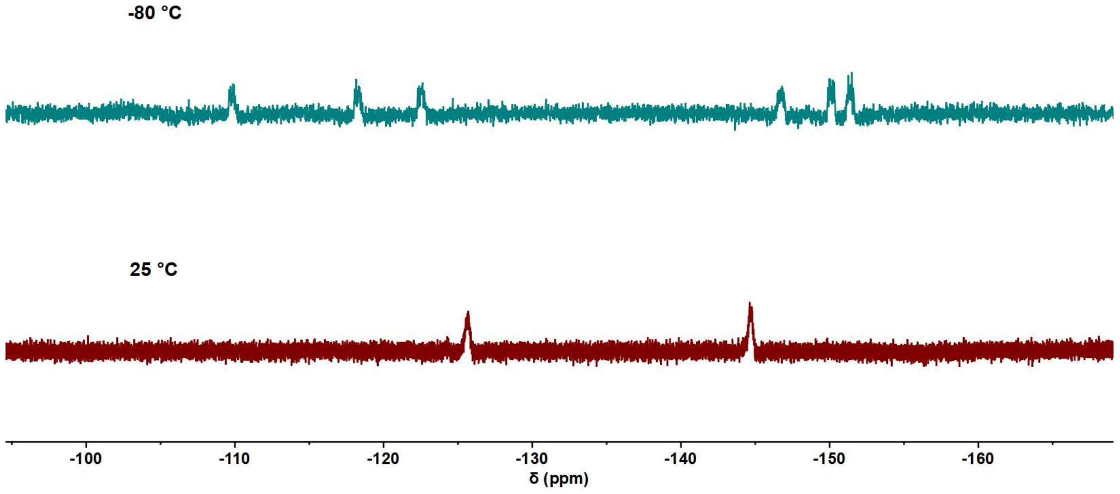

Figure S19. Comparison between the ^{19}F NMR spectra of **4·3BF$_2$** recorded at two different temperatures in THF-d_8.

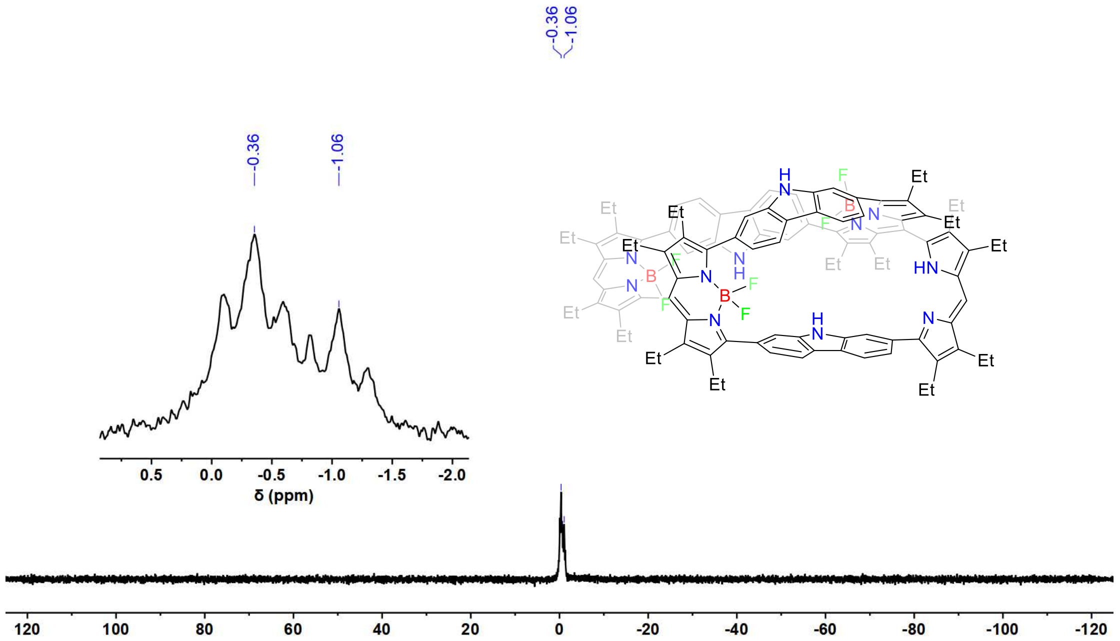

Figure S20. ^{11}B NMR spectrum of **4·3BF$_2$** recorded at 25 °C in THF-d_8.

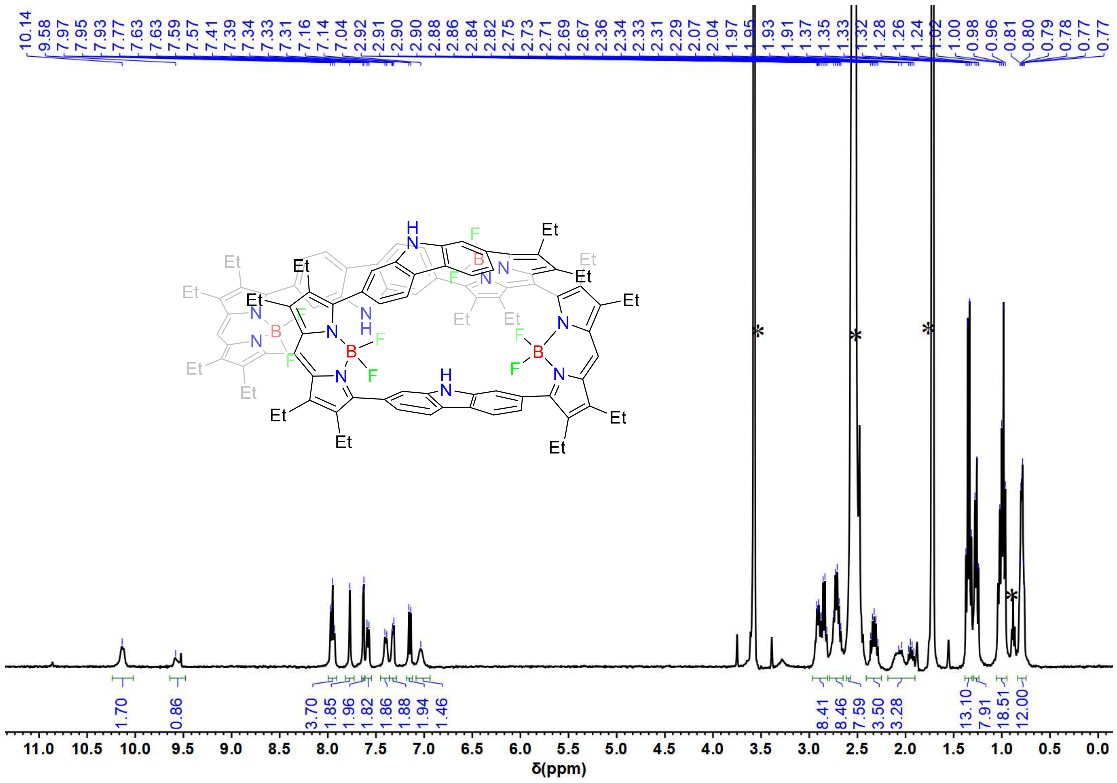

Figure S21. ^{1}H NMR spectrum of **4·4BF₂** recorded at 25 °C in THF-d_8. *Asterisks indicate residual solvent impurities.

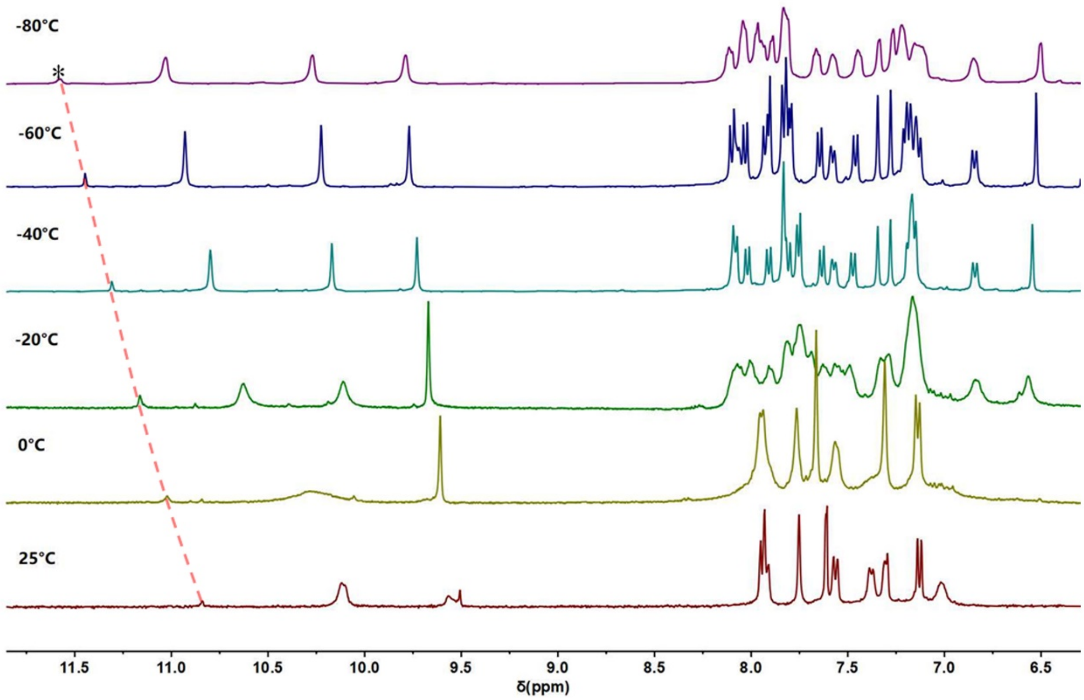

Figure S22. VT ^{1}H NMR spectra (aromatic region) of **4·4BF₂** recorded in THF-d_8.

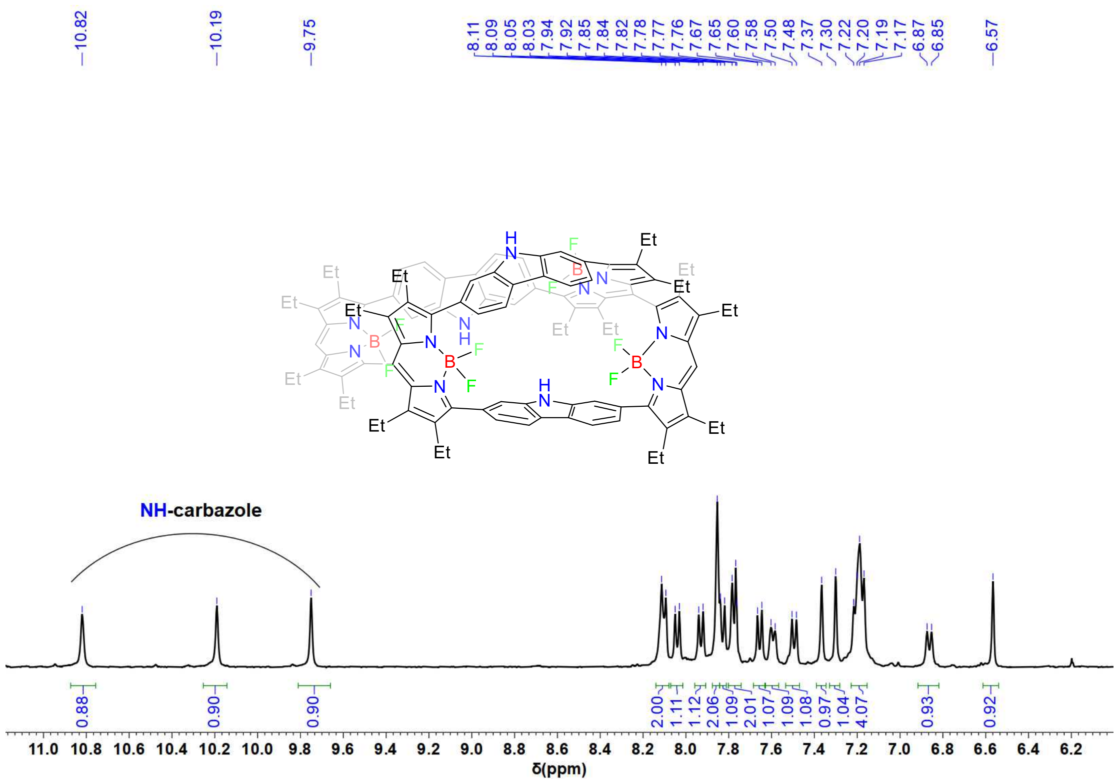

Figure S23. ^{1}H NMR spectrum (aromatic region) of **4·4BF₂** recorded at -40 °C in THF-d_8.

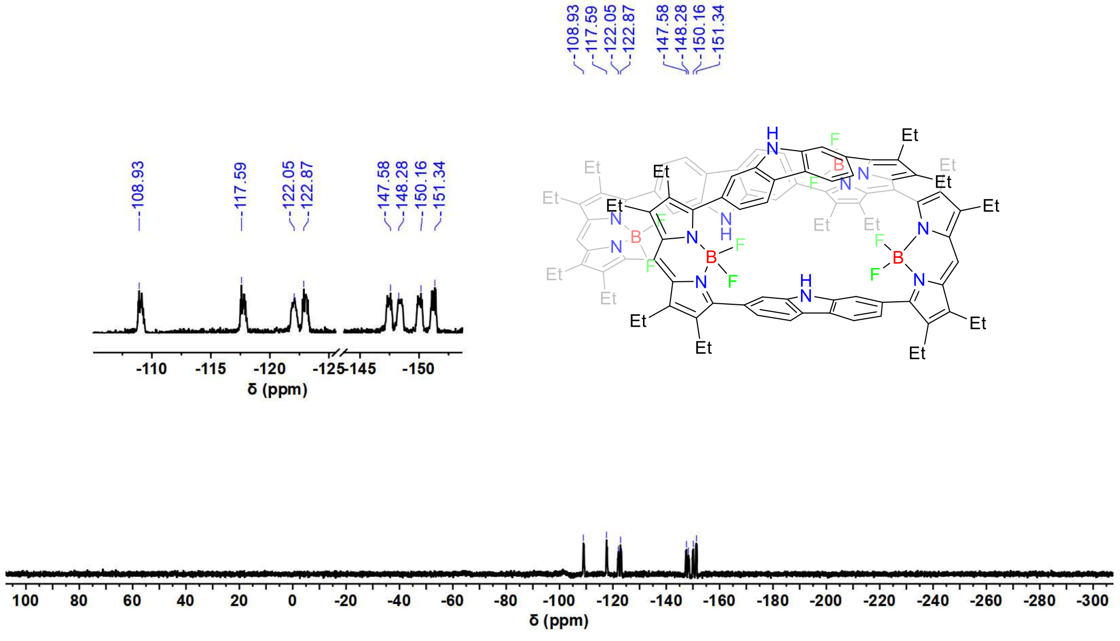

Figure S24. ^{19}F NMR spectrum of **4·4BF₂** recorded at -80 °C in THF-d_8.

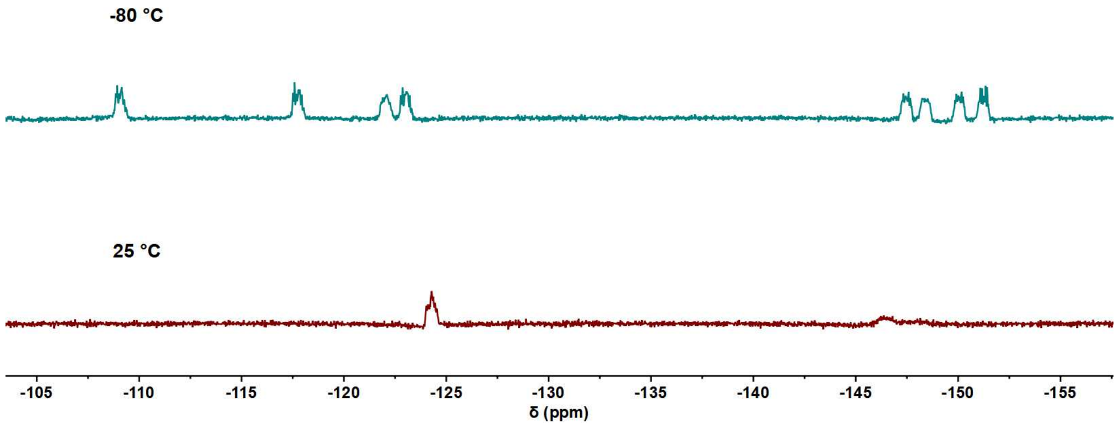

Figure S25. ^{19}F NMR spectra of **4·4BF₂** recorded at two different temperatures in THF-d_8.

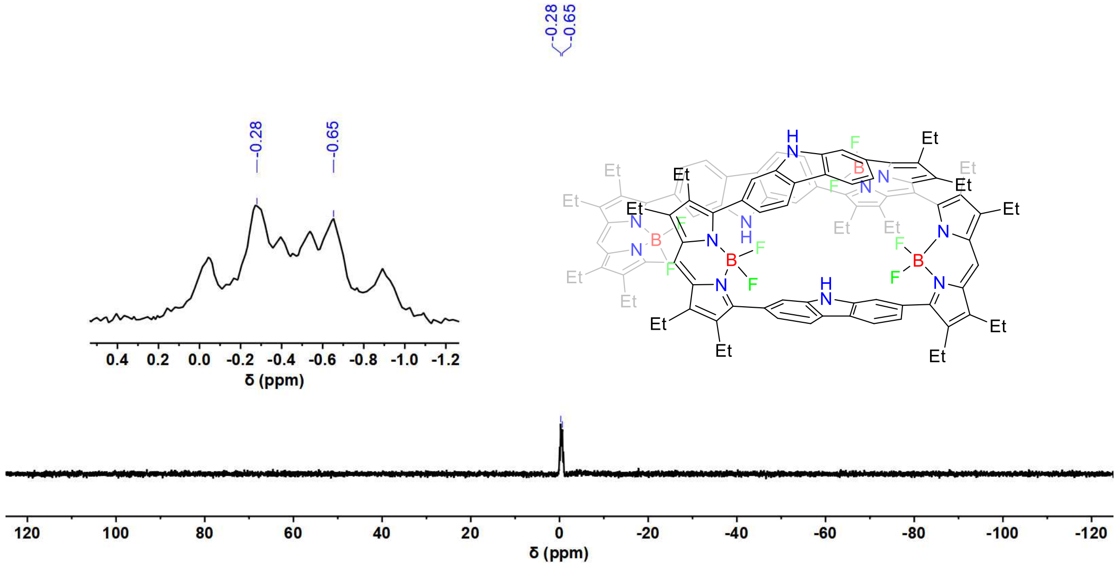

Figure S26. ^{11}B NMR spectrum of **4·4BF₂** recorded at 25 °C in THF-d_8.

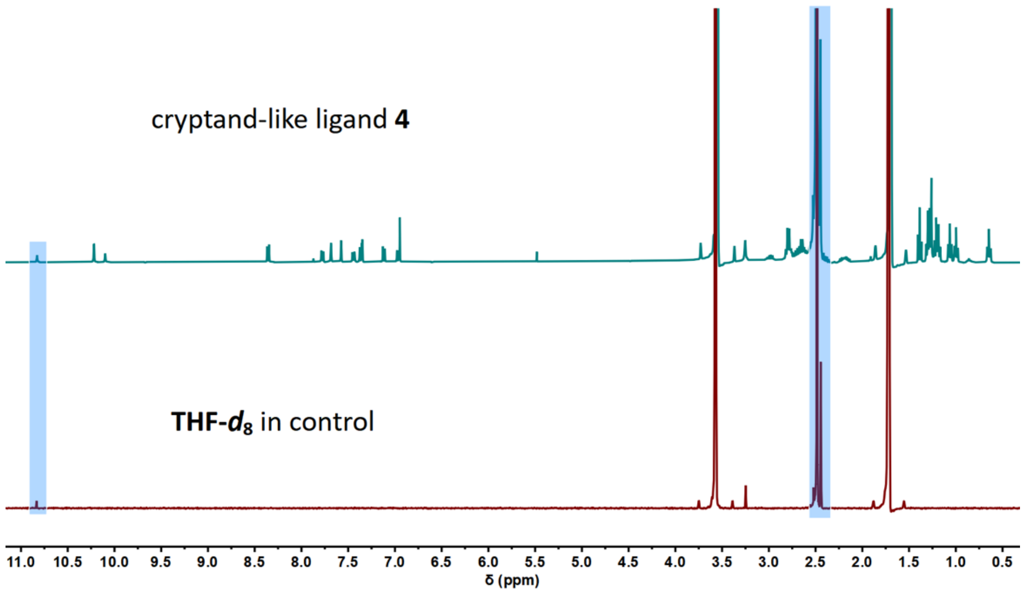

Figure S27. ^{1}H NMR spectra of THF-d_8 and **4** and at 25 °C.

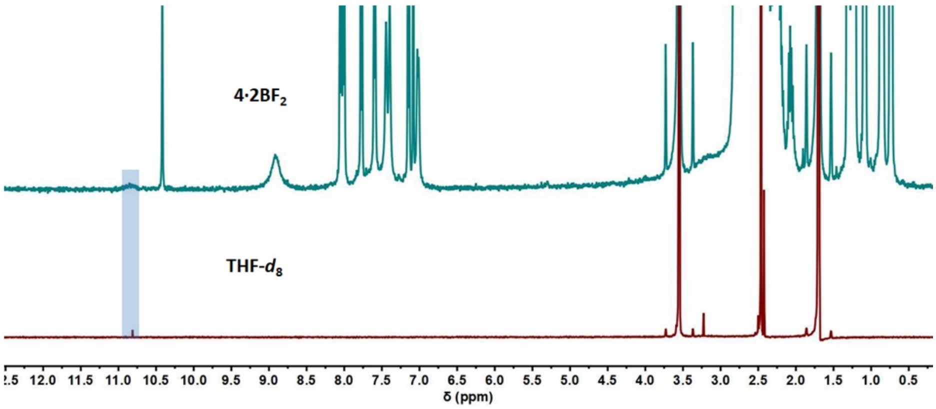

Figure S28. ^{1}H NMR spectra of THF-d_8 and **4·2BF$_2$** at 25 °C.

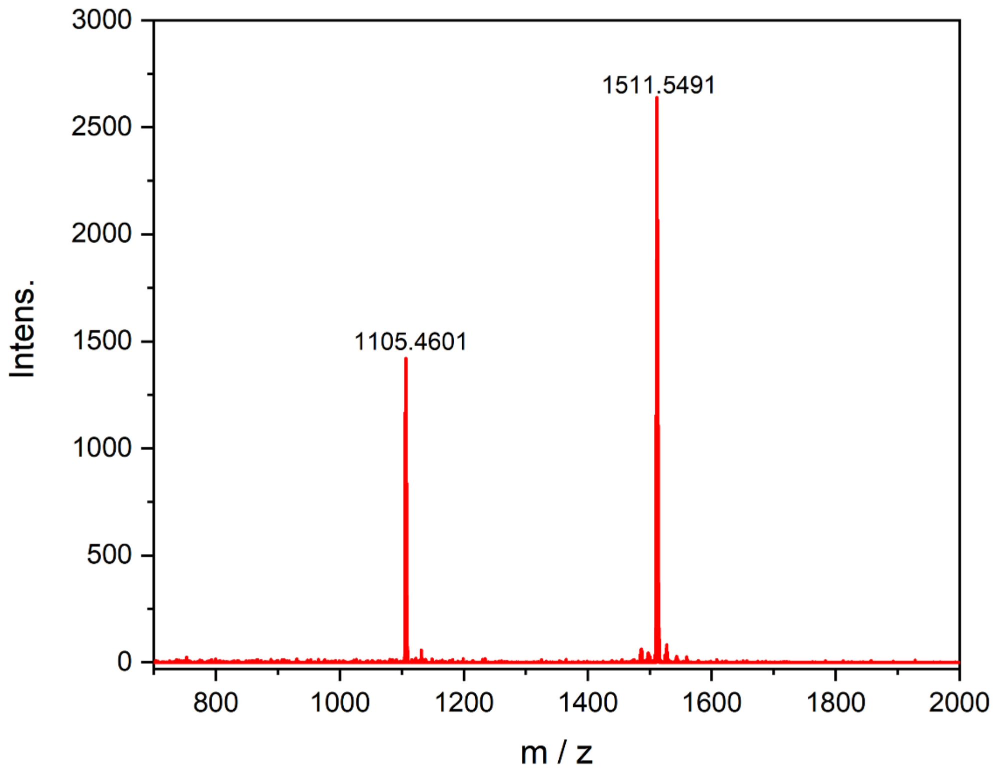

Figure S29. Observed MALDI-TOF MS spectrum of a mixture of **3** and **4** in THF.

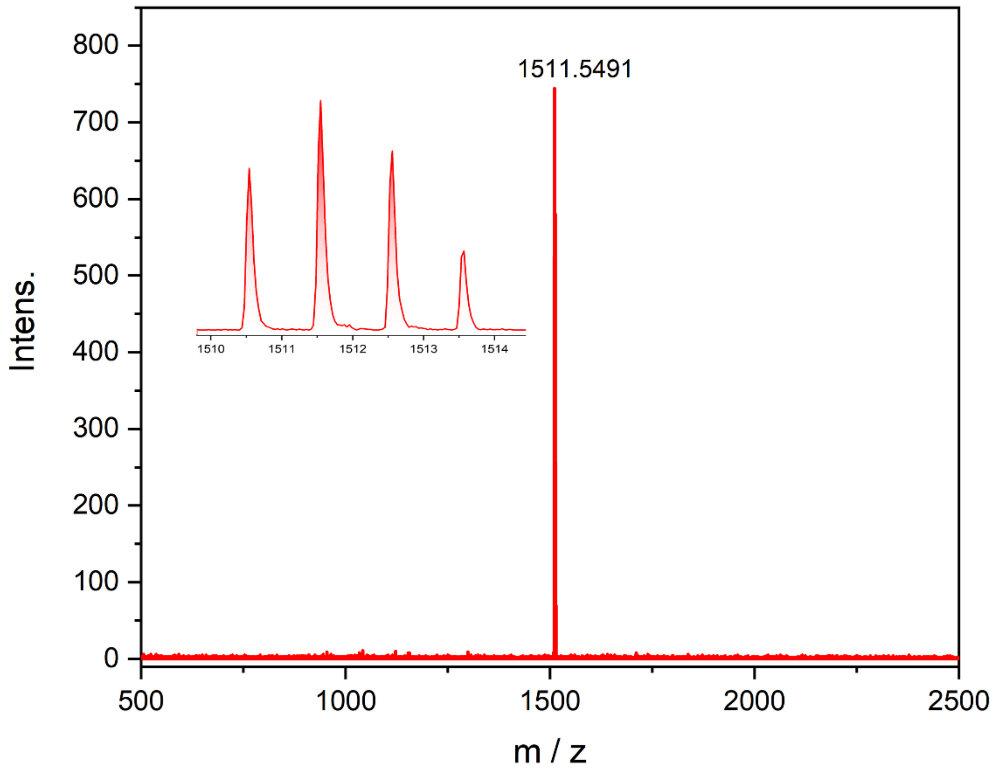

Figure S30. Observed MALDI-TOF MS spectrum of **4** in THF.

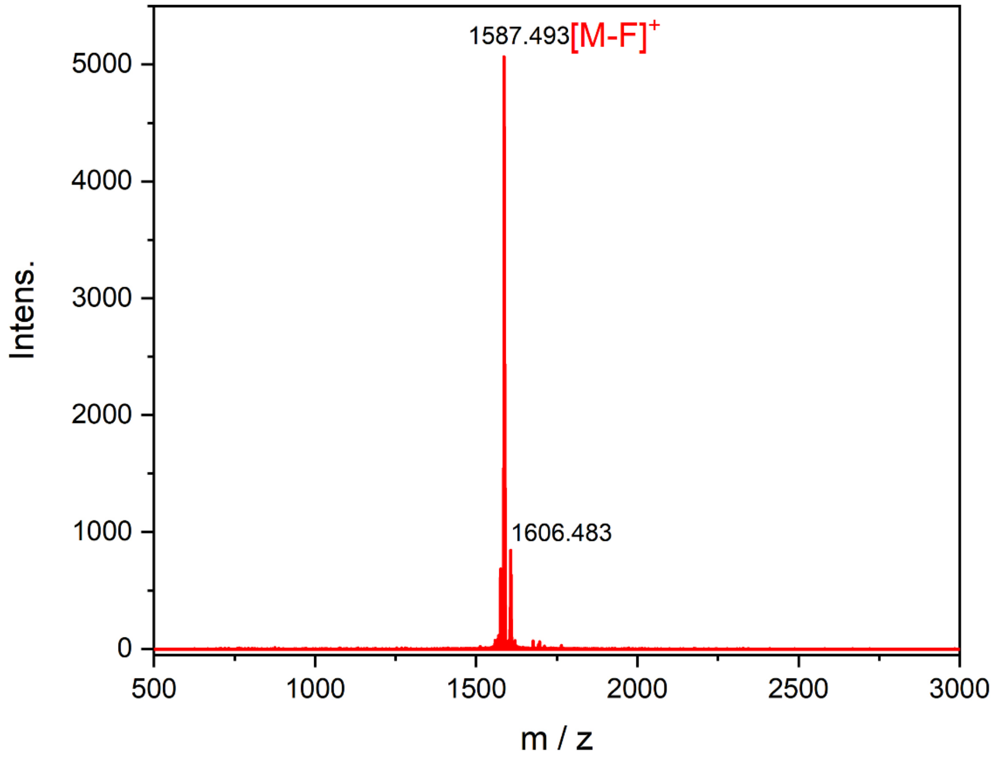

Figure S31. Observed MALDI-TOF MS spectrum of **4·2BF₂** in THF.

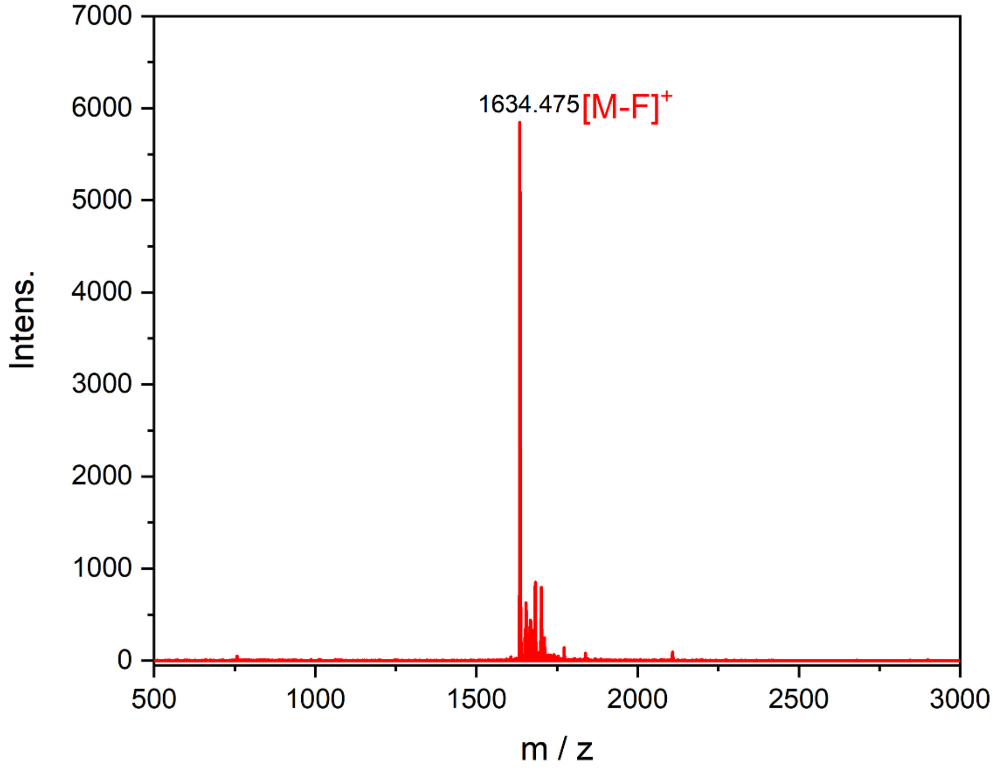

Figure S32. Observed MALDI-TOF MS spectrum of **4·3BF₂** in THF.

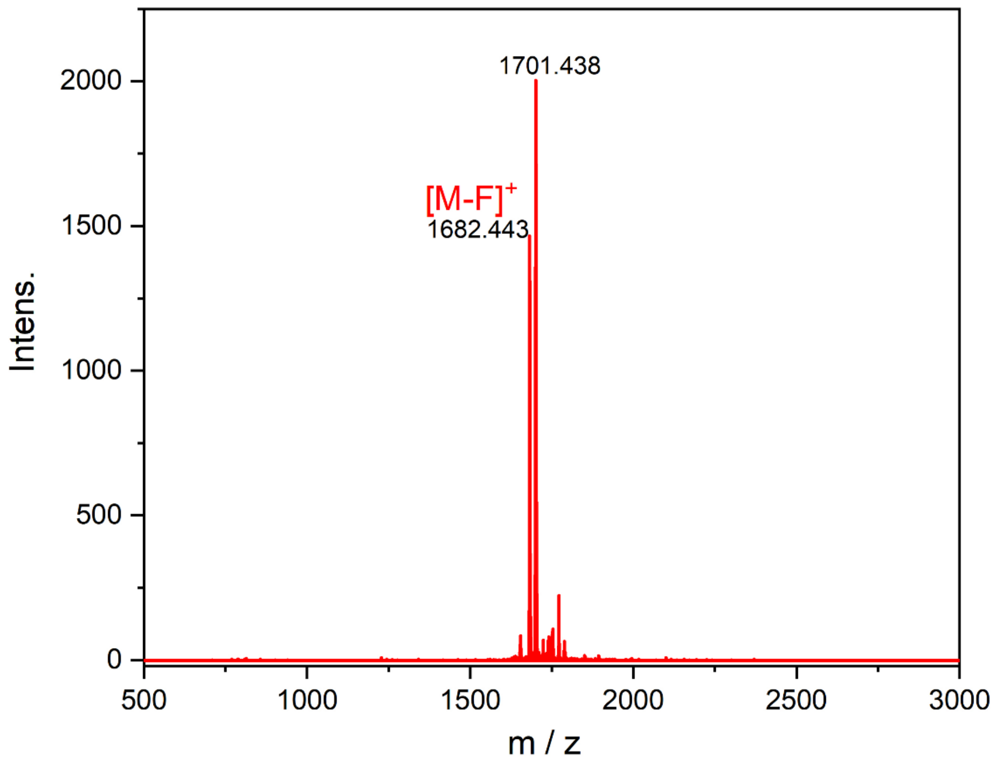

Figure S33. Observed MALDI-TOF MS spectrum of **4·4BF$_2$** in THF.

4. Crystal data

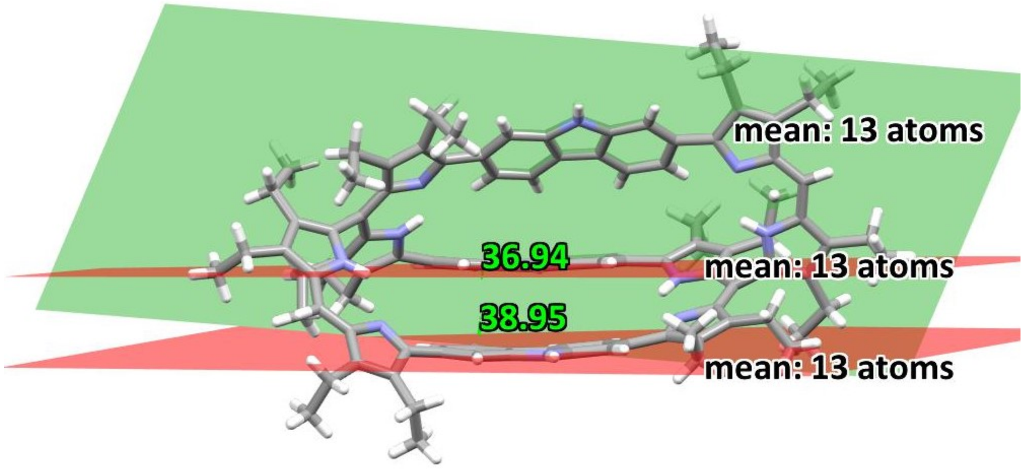

Figure S34. Single crystal X-ray diffraction structure of **4**. The dihedral angles between the mean carbazole planes are 2.01, 36.94, and 38.95°, respectively.

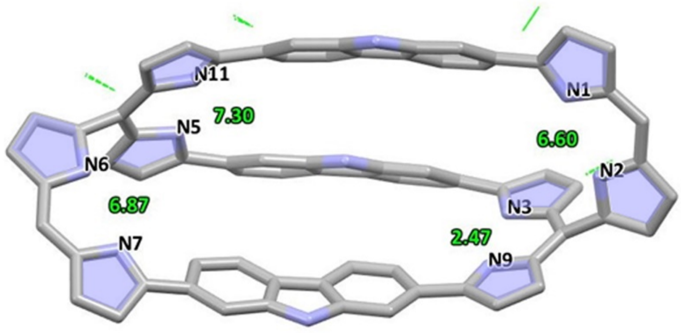

Figure S35. Single crystal X-ray diffraction structure of **4**. The dihedral angles between the mean pyrrole ring planes are 2.47, 6.60, 6.87 and 7.30°, respectively.

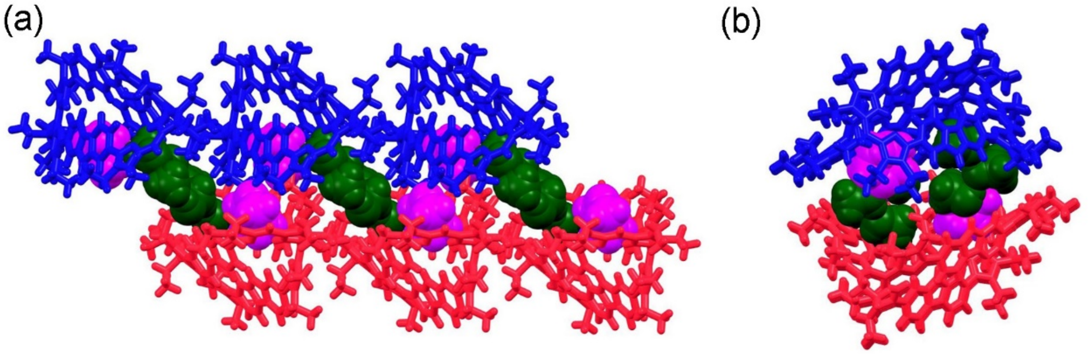

Figure S36. X-ray packing structures of **4**. (a) Top view and (b) side view. The green balls represent methanol; the purple balls represent THF.

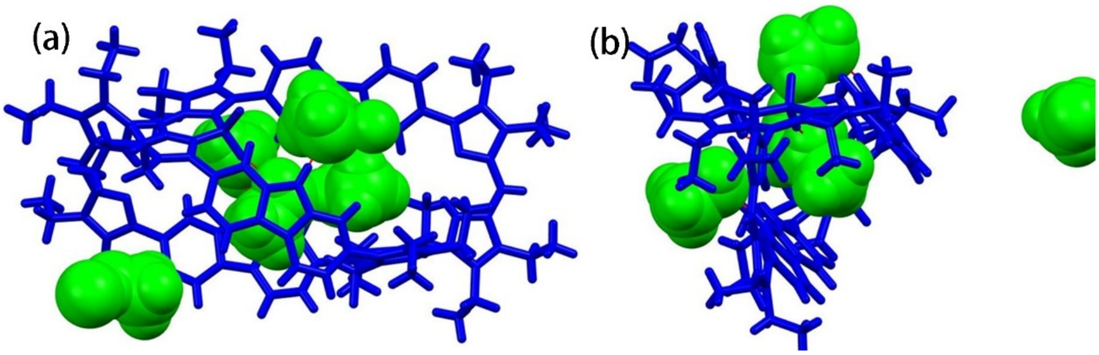

Figure S37. X-ray crystal structures of CH_3OH@**4·2BF₂**. (a) Top view and (b) side view. The green balls represent methanol and the blue sticks represent **4·2BF₂**.

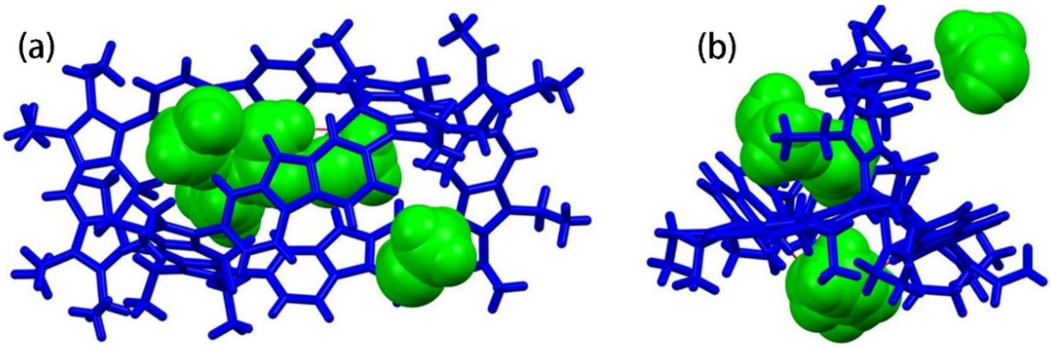

Figure S38. X-ray crystal structures of CH_3OH@**4·4BF₂**. (a) Top view and (b) side view. The green balls represent methanol and the blue stick represent **4·4BF₂**.

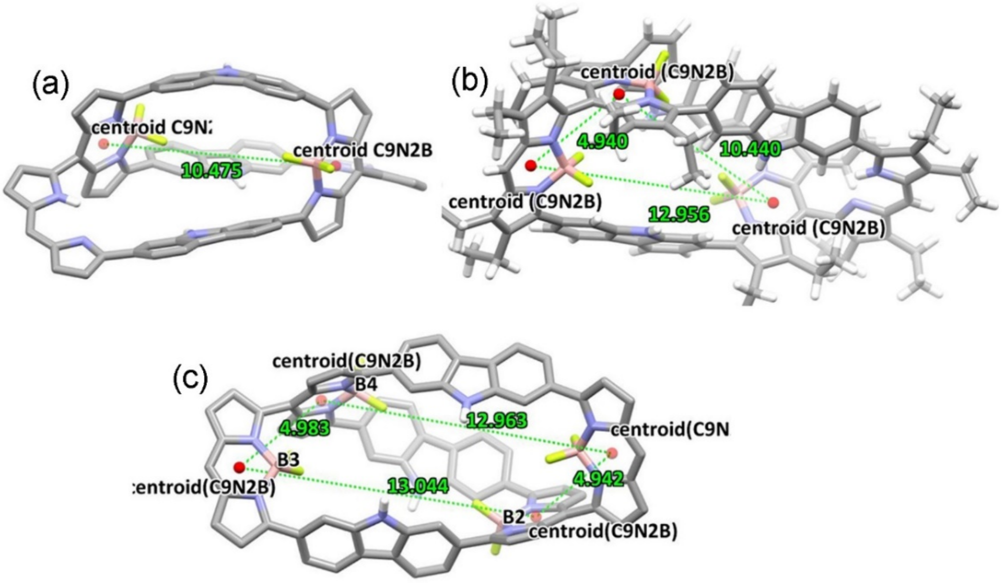

Figure S39. X-ray crystal structures of (a) **4·2BF₂**, (b) **4·3BF₂**, and (c) **4·4BF₂**. The indicated distances are provided to give a sense of the size of the BODIPY chromophore centroid.

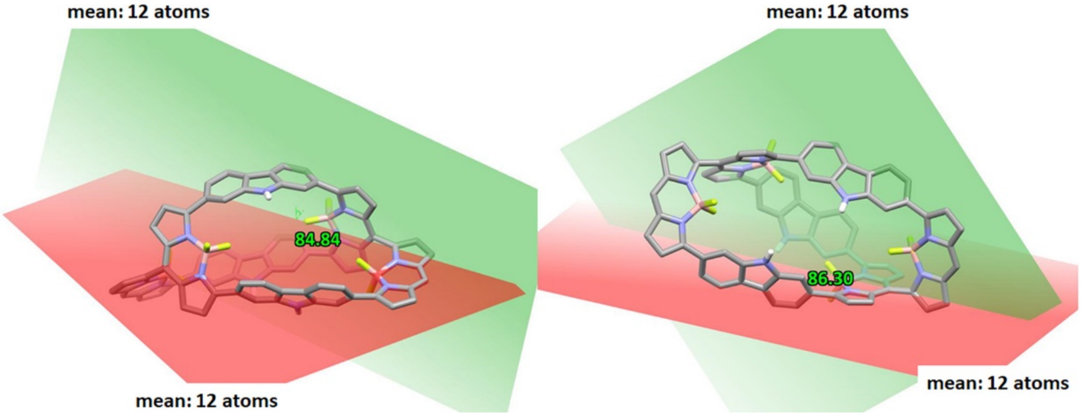

Figure S40. X-ray crystal structure of **4·4BF₂** showing the dihedral angle between adjacent mean BODIPY planes.

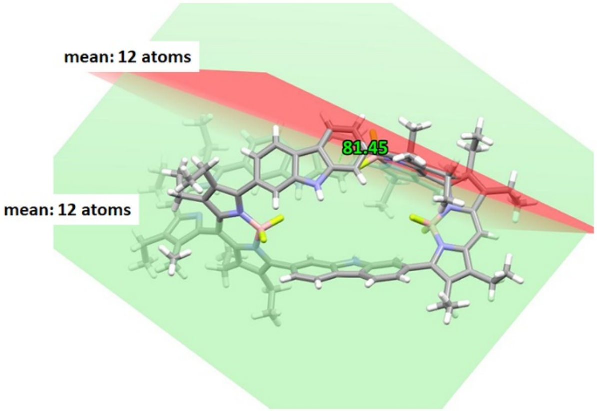

Figure S41. X-ray crystal structure of **4·3BF₂** showing the dihedral angle between adjacent mean BODIPY planes.

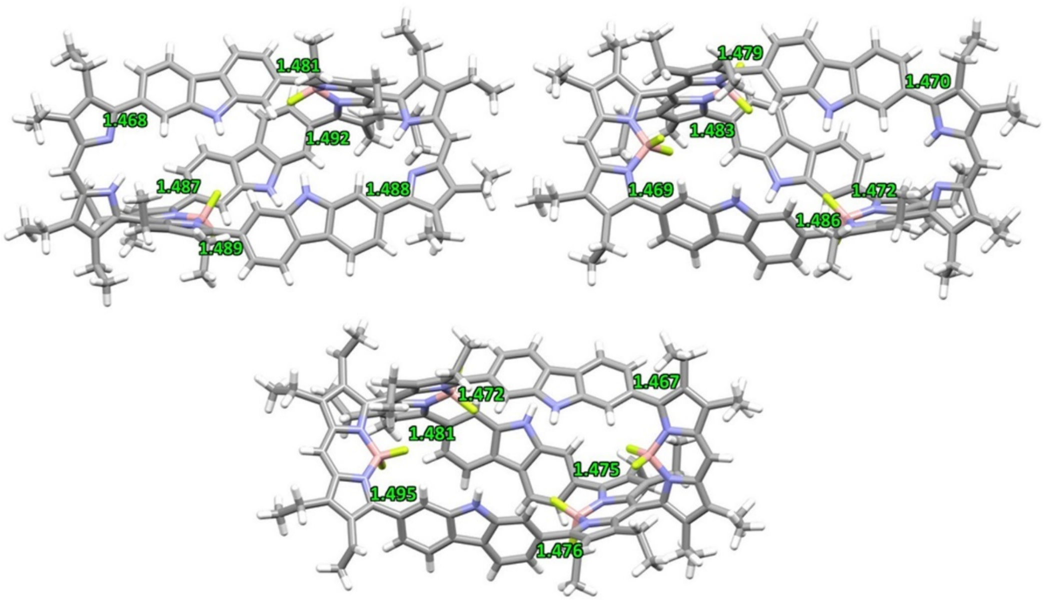

Figure S42. X-ray crystal structures of **4·3BF₂** showing the dihedral angle between adjacent mean BODIPY planes.

Table S2. Crystal data and structure refinements for **4** and **4·2BF$_2$**.

**Compound**	**4**	**4·2BF$_2$**
Formula	$C_{104}H_{107}N_{11}$, 3(CH_4O), C_4H_8O	$C_{104}H_{105}B_2F_4N_{11}$, 2($CH_2Cl_2$), 5($CH_4O$)
M_r	1679.23	1936.66
Temperature/K	173.0	173.0
Crystal system	Triclinic	Orthorhombic
Space group	*P* -1<br>2	*P* b c n<br>60
a/Å	14.7293(4)	38.9356(10)
b/Å	18.9653(6)	18.8132(4)
c/Å	22.0241(6)	29.4619(7)
α/°[deg]	66.8150(10)	90
β/°[deg]	72.3480(10)	90
γ/°[deg]	74.252(2)	90
Volume[Å3]	5307.4(3)	21580.9(9)
Z	2	8
ρcalc[g/cm^3]	1.051	1.192
Radiation	CuKα (λ = 1.54178)	CuKα (λ = 1.54178)
Reflections collected	50076	234927
Goodness-of-fit on F^2	1.818	1.340
Final R indices [I>=2σ (I)]	R1 = 0.1251, wR2 = 0.3110	R1 = 0.1029, wR2 = 0.3092
R indices [all data]	R1 = 0.1789, wR2 = 0.3375	R1 = 0.1328, wR_2 = 0.3439
CCDC no.	2110148	2150674
Solvents	THF/CH_3OH	CH_2Cl_2/CH_3OH

Table S3. Crystal data and structure refinements for **4·3BF$_2$** and **4·4BF$_2$**.

Compound	4·3BF$_2$	4·4BF$_2$
Formula	$C_{104}H_{104}B_3F_6N_{11}$	$C_{104}H_{103}B_4F_8N_{11}$, 5($CH_4O$)
M_r	1654.41	1860.40
Temperature/K	150.0	173.0
Crystal system	Triclinic	Orthorhombic
Space group	*P* -1<br>2	*P* 2_1 2_1 2_1<br>19
a/Å	12.9759(5)	17.2251(4)
b/Å	17.3000(6)	23.1997(7)
c/Å	23.1532(8)	28.8441(8)
α/°[deg]	82.590(2)	90
β/°[deg]	81.602(2)	90
γ/°[deg]	76.517(2)	90
Volume[Å^3]	4975.7(3)	11526.6(5)
Z	2	4
ρcalc[g/cm^3]	1.104	1.072
Radiation	CuKα (λ = 1.54178)	CuKα (λ = 1.54178)
Reflections collected	129483	76414
Goodness-of-fit on F^2	1.056	1.002
Final R indices [I>=2σ (I)]	R1 = 0.0881, wR2 = 0.2400	R1 = 0.0823, wR2 = 0.2307
R indices [all data]	R1 = 0.1316, wR2 = 0.2826	R1 = 0.0994, wR2 = 0.2518
CCDC no.	2150676	2150675
Solvents	CH_2Cl_2/CH_3OH	THF/CH_3OH

5. Optical and electrochemical studies

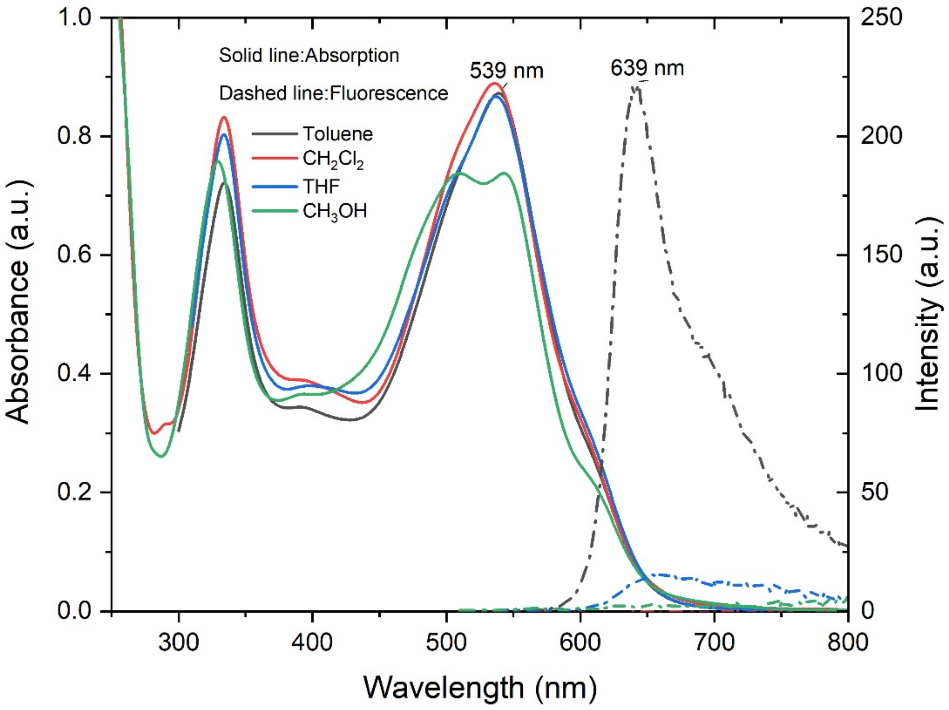

Figure S43. Comparative absorption (solid line) and fluorescence (dashed line) spectra of **4·2BF$_2$** (10^{-5} M) in various solvents. Excitation wavelength = 500 nm; slit width = 3 nm.

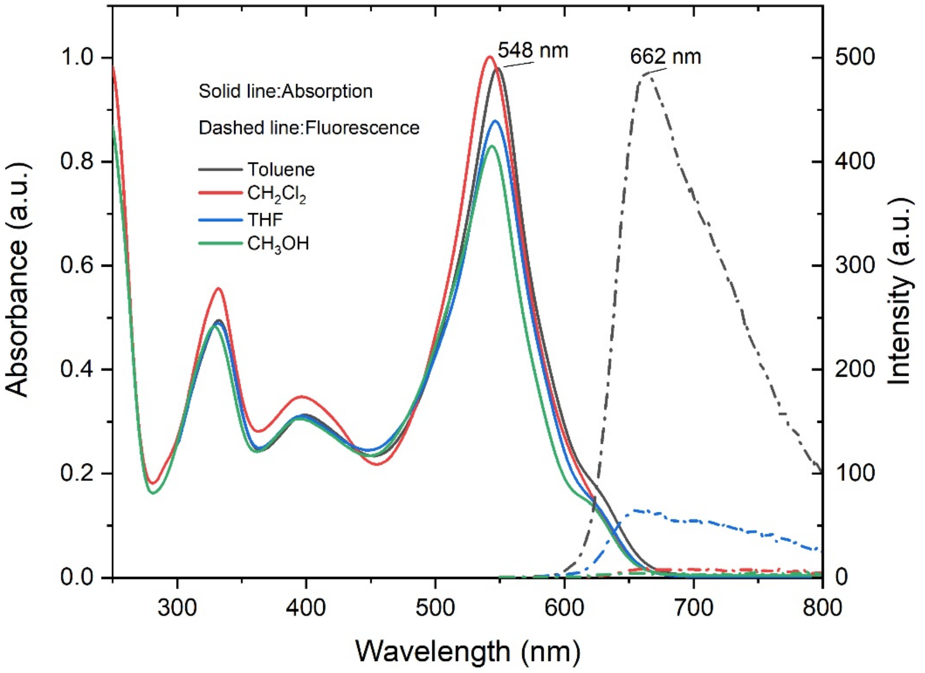

Figure S44. Comparative absorption (solid line) and fluorescence (dashed line) spectra of **4·3BF$_2$** (10^{-5} M) in various solvents. Excitation wavelength = 510 nm; slit width = 3 nm.

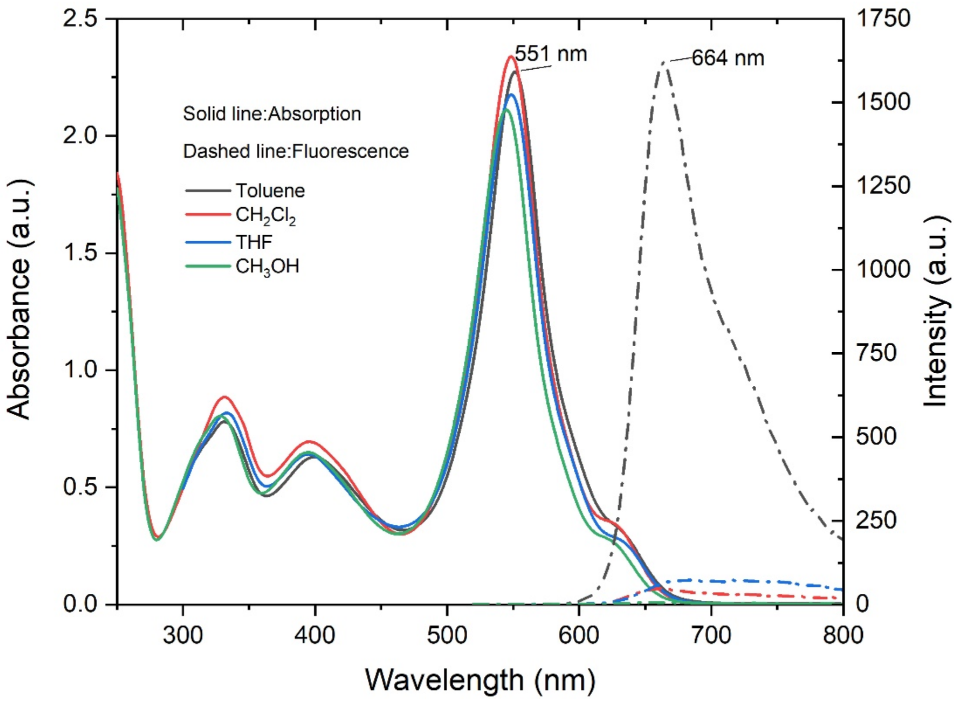

Figure S45. Comparative absorption (solid line) and fluorescence (dashed line) spectra of **4·4BF$_2$** (10^{-5} M) in various solvents. Excitation wavelength = 540 nm; slit width = 3 nm.

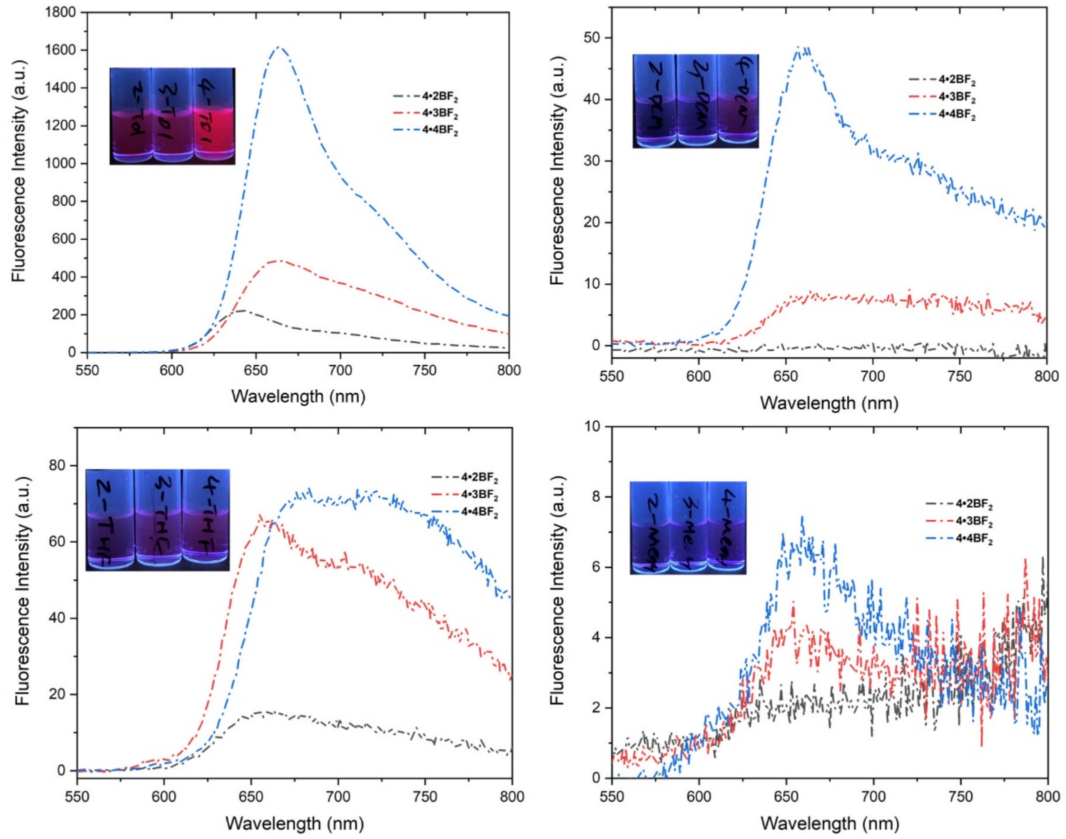

Figure S46. Comparative fluorescence spectra (10^{-5} M) recorded for **4·2BF$_2$**, **4·3BF$_2$** and **4·4BF$_2$** in various solvents: (a) toluene, (b) CH_2Cl_2, (c) THF, and (d) CH_3OH. Inset: Images of the solutions under 365 nm light. Excitation wavelengths of 500 nm, 510 nm and 540 nm were used with a slit width of 3 nm in all cases.

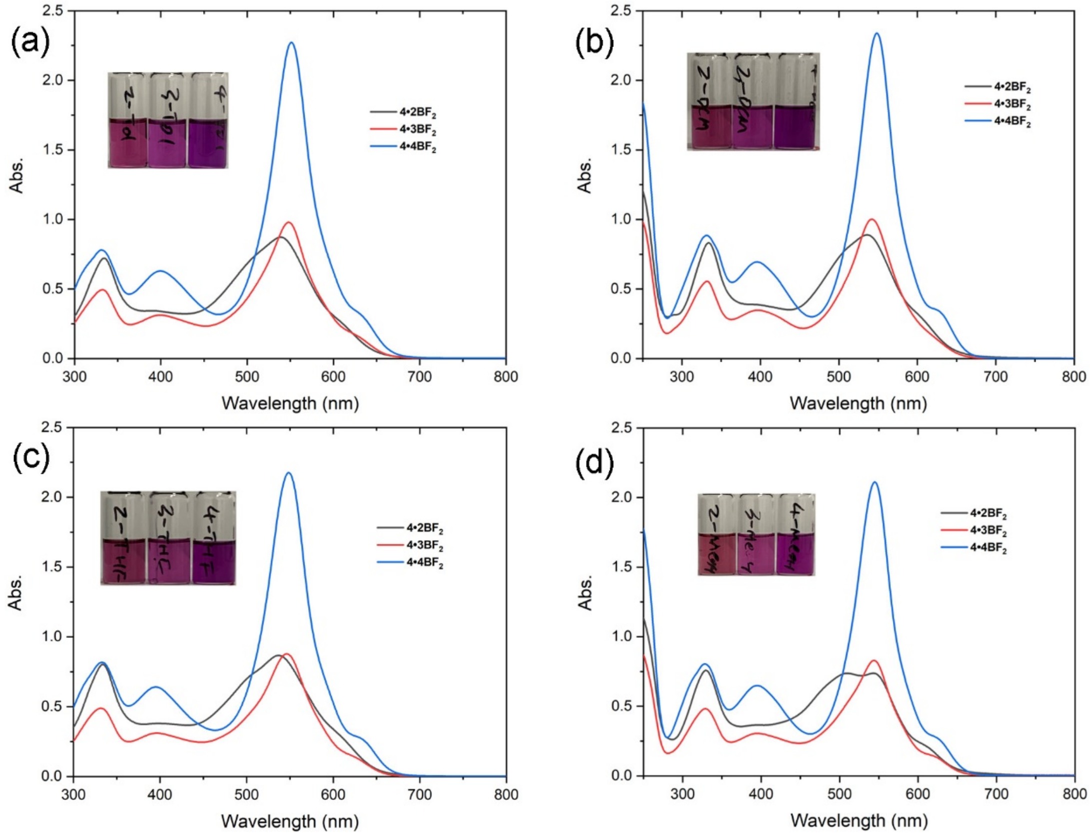

Figure S47. Absorption spectra (10^{-5} M) of **4·2BF$_2$**, **4·3BF$_2$** and **4·4BF$_2$** recorded in various solvents: (a) toluene, (b) CH_2Cl_2, (c) THF, and (d) CH_3OH. Inset: Images of the solution under ambient light.

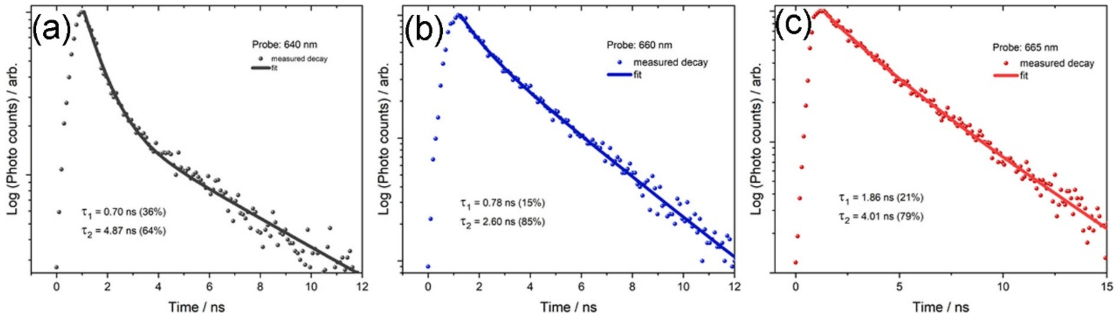

Figure S48. Fluorescence decay profiles of (a) **4·2BF$_2$**, (b) **4·3BF$_2$**, and (c) **4·4BF$_2$** in toluene

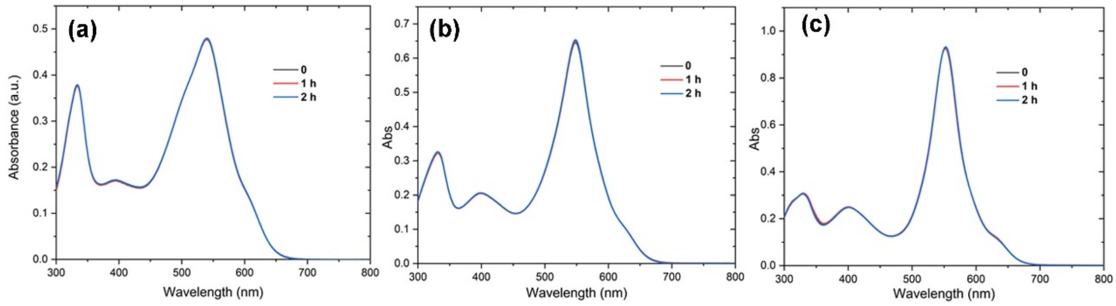

Figure S49. Absorbance spectra of (a) **4·2BF$_2$**, (b) **4·3BF$_2$**, and (c) **4·4BF$_2$** recorded in toluene under conditions of photoirradiation (520 nm, 200 mW·cm^{-2}) for up to 2 h.

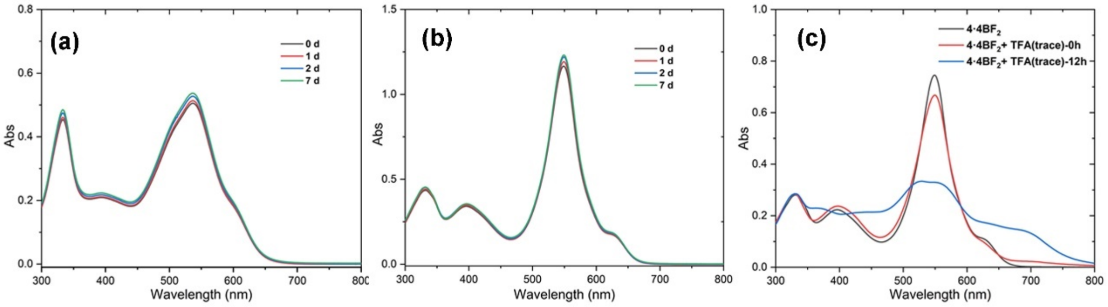

Figure S50. Absorbance spectra (a) **4·2BF$_2$**, (b) **4·4BF$_2$** and (c) **4·4BF$_2$** recorded after adding a trace quantity of TFA in CH_2Cl_2.

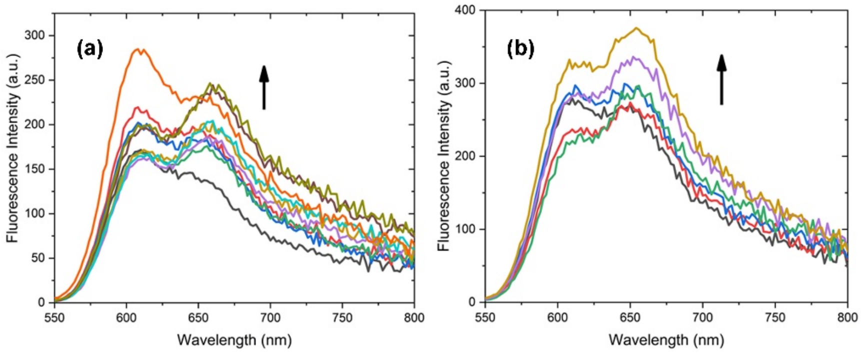

Figure S51. Viscosity-dependent fluorescence spectra of **4·4BF$_2$** in (a) methanol - ethylene glycol mixtures at various ratios (V:V from 10:0→1:9) and (b) methanol – glycerol systems at various ratios (V:V from 10:0→5:5). Excitation wavelength = 540 nm; slit width = 2 nm. Note: With increasing glycerol ratios, some precipitation occurs.

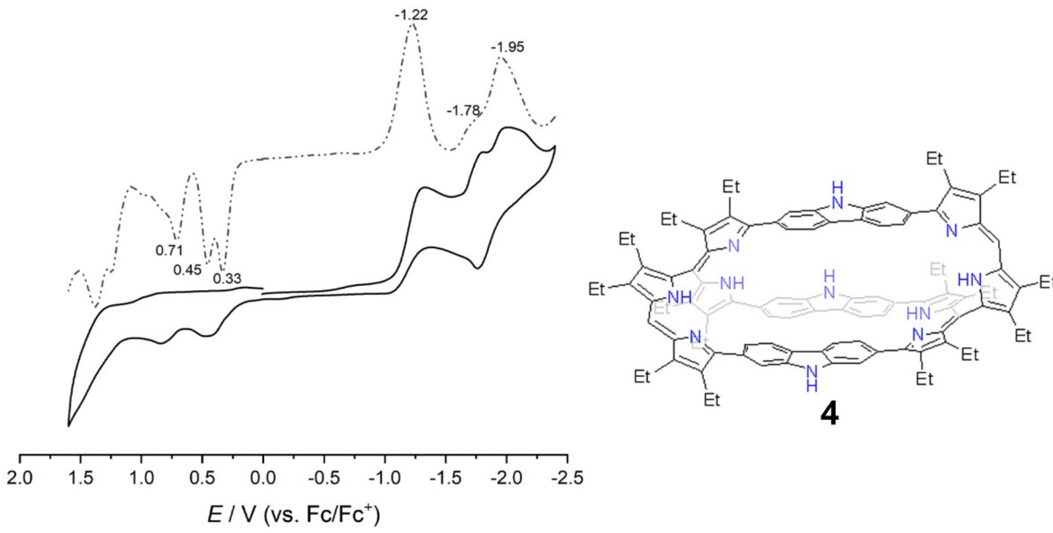

Figure S52. Cyclic voltammogram and differential pulse voltammogram of **4**.

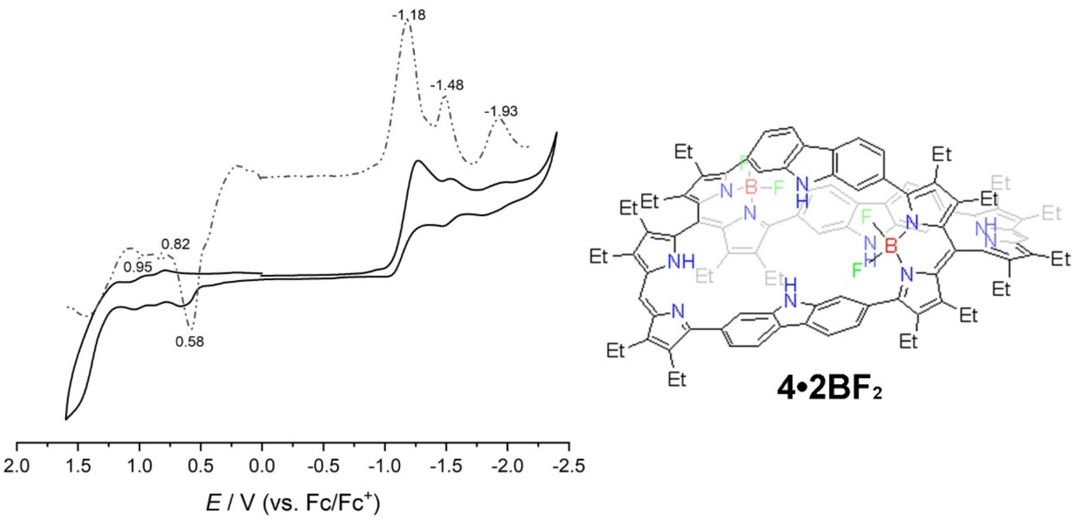

Figure S53. Cyclic voltammogram and differential pulse voltammogram of **4·2BF$_2$**.

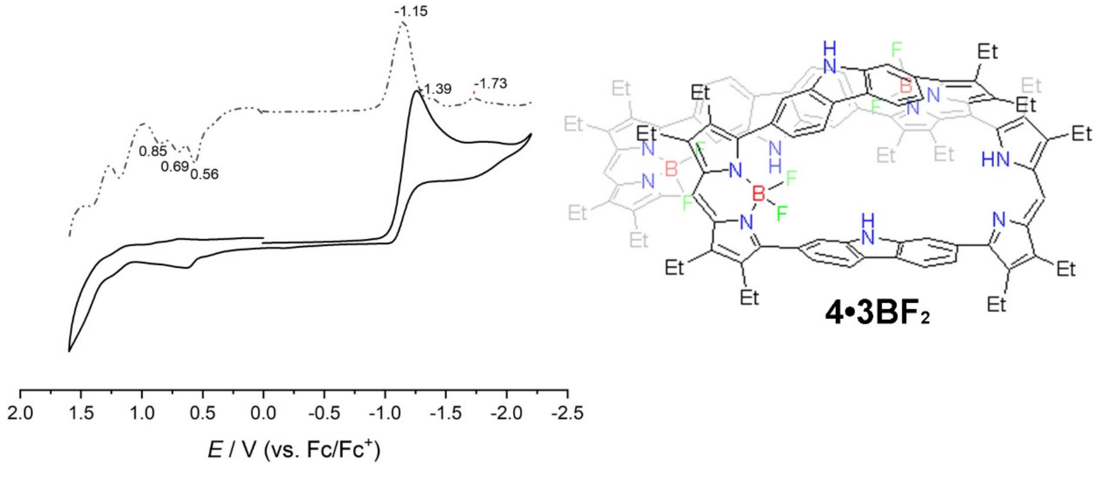

Figure S54. Cyclic voltammogram and differential pulse voltammogram of **4·3BF$_2$**.

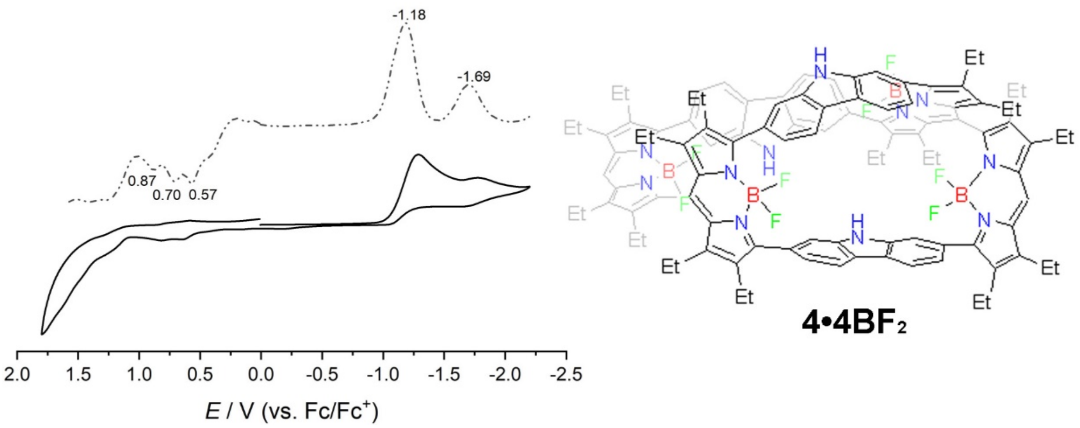

Figure S55. Cyclic voltammogram and differential pulse voltammogram of **4·4BF$_2$**.

6. Singlet oxygen measurements

A comparative study of the relative singlet oxygen generating efficiencies of these BODIPY arrays (≈ 5×10^{-6} M in CH_2Cl_2) was performed in air-saturated solvents under 520 nm irradiation (20 mW·cm^{-2}) using 1,3 diphenylisobenzofuran (DPBF, ≈2×10^{-4} M) as a singlet oxygen trap and Rose Bengal (**RB**, Φ_Δ = 80% in methanol) as the reference compound, respectively.[4] The decrease in the absorbance band of DPBF was monitored (Figures S51-S55). Singlet oxygen quantum yield (Φ_Δ) determinations were carried out using the chemical trapping method. The Φ_Δ value was obtained by comparison in accord with equation S1:

$$\Phi_\Delta(^1O_2)^{BODIPY} = \Phi_\Delta(^1O_2)^{RB} \frac{S^{BODIPY} F^{RB}}{S^{RB} F^{BODIPY}} \qquad \textbf{Equation S1}$$

where $\Phi_\Delta(^1O_2)$ is the quantum yield of singlet oxygen, superscripts "BODIPY" and "RB" represent **4·nBF$_2$** and Rose Bengal, respectively. "S" is the slope of a plot of the change in absorbance of DPBF (at 414 nm for BODIPY complexes and 411 nm for **RB**) as a function the irradiation time (Figures S56-59), and "F" is the absorption correction factor, which is given by F =1 − 10^{-OD} (where OD is the optical density at the irradiation wavelength of 520 nm).

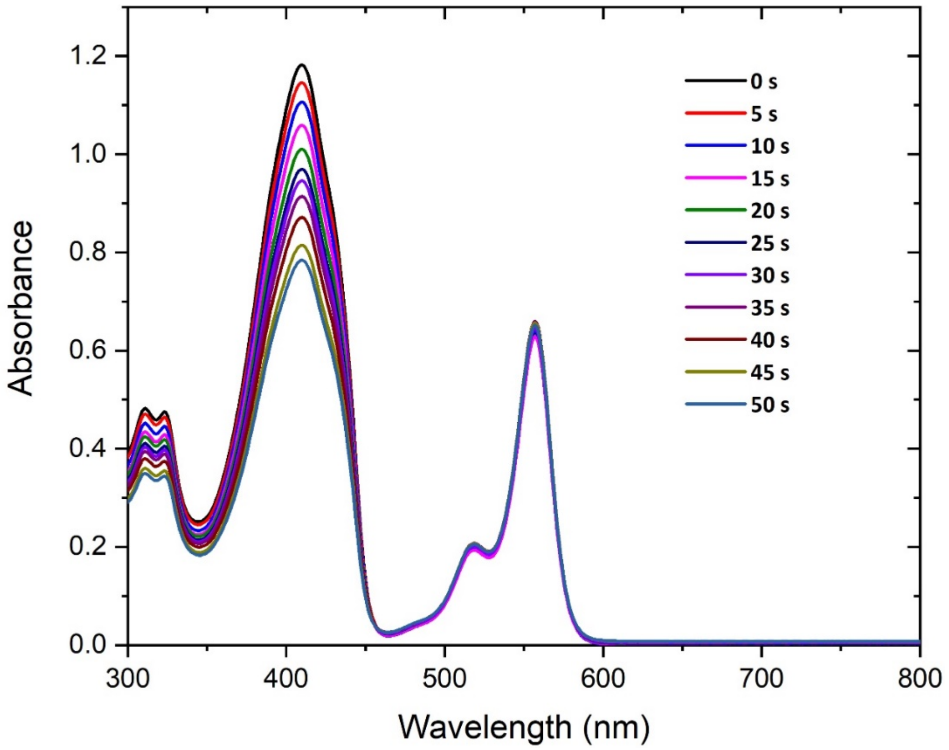

Figure S56. Changes in the absorption spectra of DPBF seen upon irradiation (λ_{irr} = 520 nm) in MeOH for the indicated times in the presence of **RB** used as a positive control.

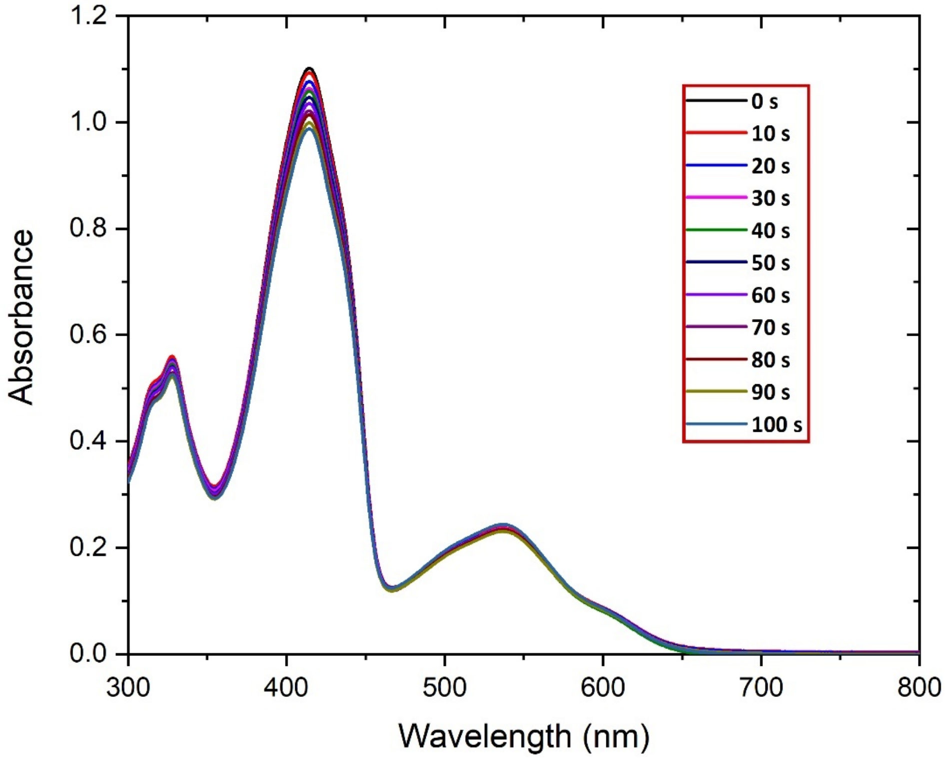

Figure S57. Changes in the absorption spectra of DPBF upon irradiation (λ_{irr} = 520 nm) at the indicated intervals in the presence of **4·2BF$_2$**.

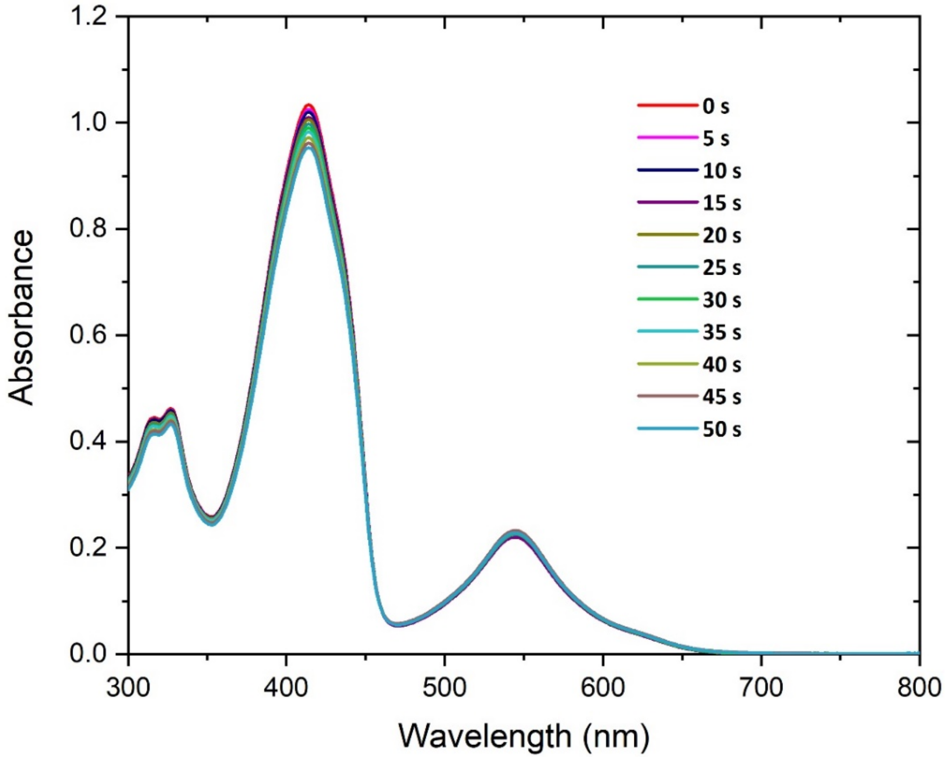

Figure S58. Changes in the absorption spectra of DPBF seen upon irradiation (λ_{irr} = 520 nm) in CH_2Cl_2 for the indicated times in the presence of **4·3BF$_2$**.

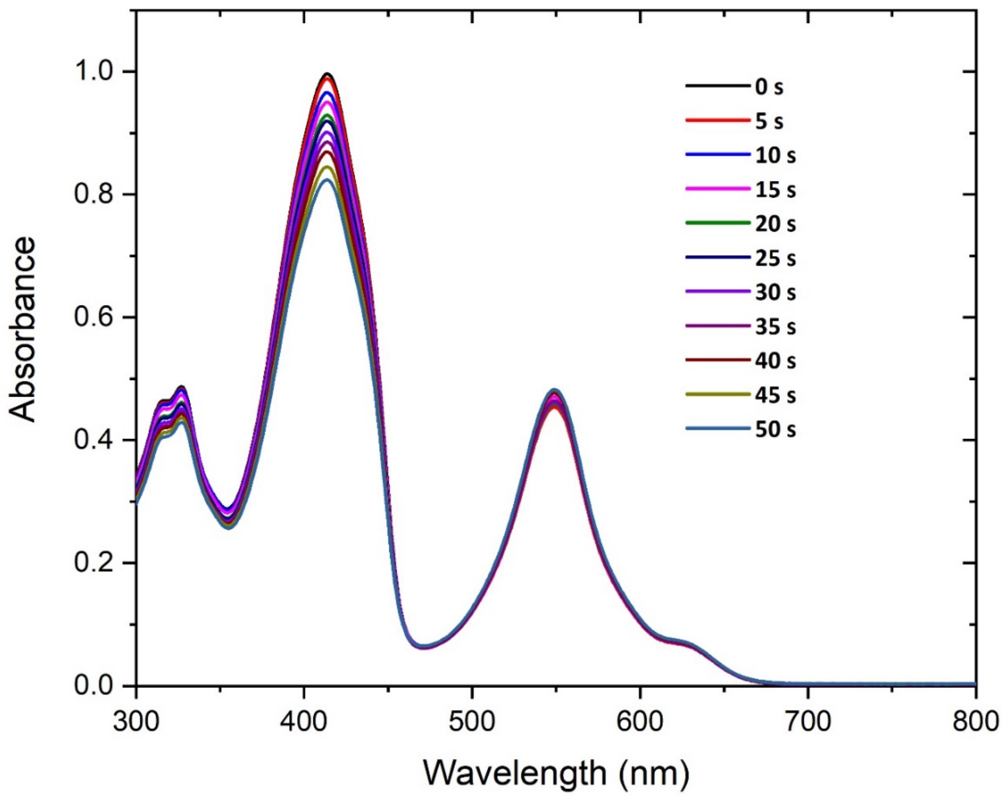

Figure S59. Changes in the absorption spectra of DPBF seen upon irradiation (λ_{irr} = 520 nm) in CH_2Cl_2 for the indicated times in the presence of **4·4BF₂**.

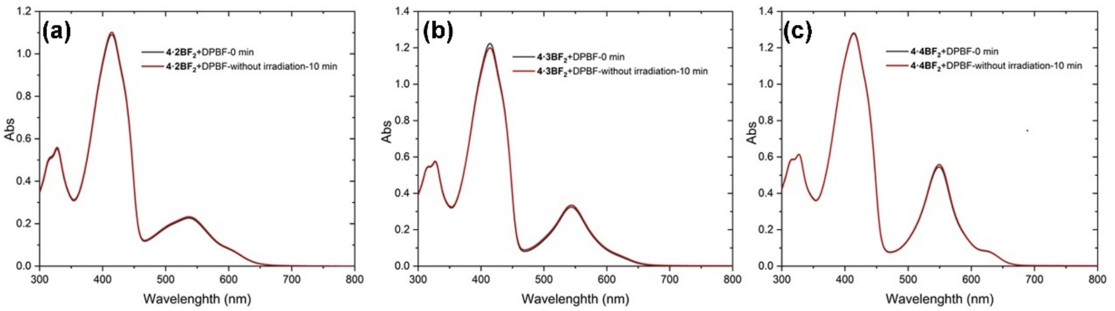

Figure S60. Changes in the absorption spectra of DPBF before and after allowing to stand in the presence of DPBF and (a) **4·2BF$_2$**, (b) **4·3BF$_2$** and (c) **4·4BF$_2$** for 10 min in CH_2Cl_2 in the dark.

7. DFT calculations

All calculations were carried out using the Gaussian 16 program.[5] Initial geometries for **4**, **4·2BF$_2$**, **4·3BF$_2$**, and **4·4BF$_2$** were obtained from the X-ray structures. The structures were fully optimized without any symmetry restrictions. Geometry optimizations in the ground state (S0) were performed using density functional theory (DFT) with Becke's three-parameter hybrid exchange functionals and the Lee-Yang-Parr correlation functional (B3LYP) employing the 6-311G (d, p) basis set for all atoms.[6] NICS (0) values[7] were calculated with GIAO method at the B3LYP level employing the same basis sets for geometry optimizations. TD-DFT computations used the optimized ground state geometries and were carried out at the CAM-B3LYP/6-311G (d, p) level.[8] The calculated structures in toluene were obtained using the Self-Consistent Reaction Field (SCRF) method and a Polarizable Continuum Model (PCM).[9]

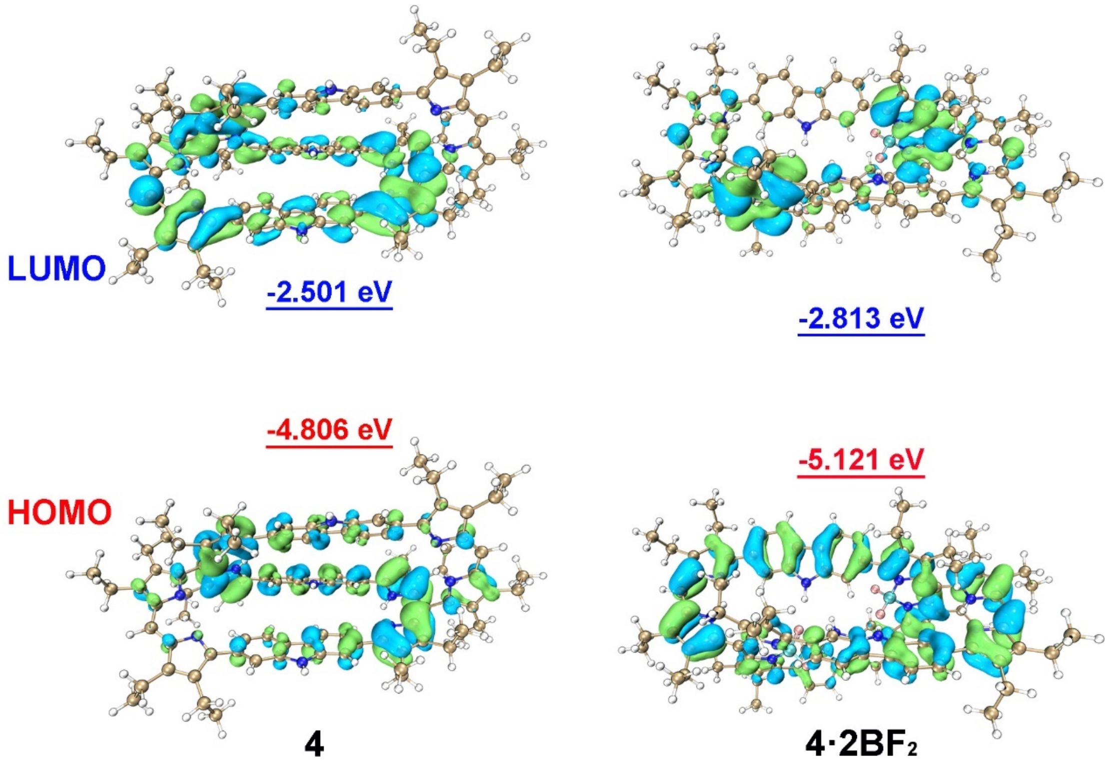

Figure S61. Pictorial presentation of the LUMO, HOMO and their energy levels for **4** and **4·2BF$_2$** calculated at the B3LYP/6-311G(d,p) level.

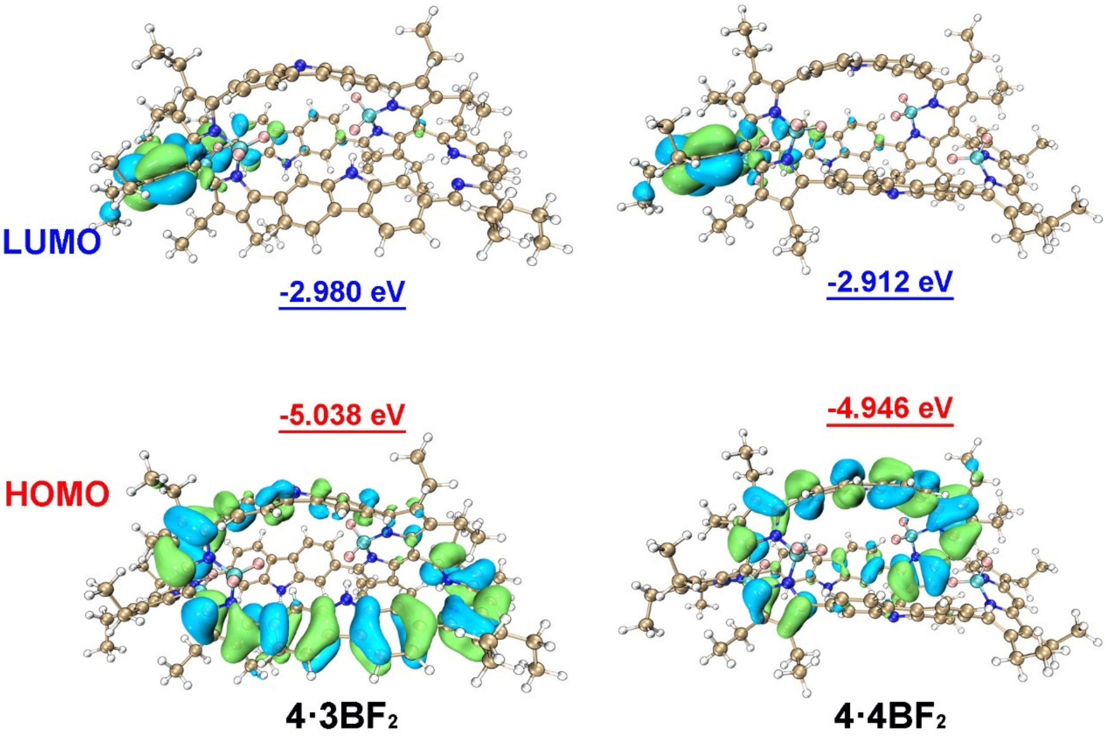

Figure S62. Pictorial presentation of the LUMO, HOMO and their energy levels for **4·3BF$_2$** and **4·4BF$_2$** calculated at the B3LYP/6-311G(d,p) level.

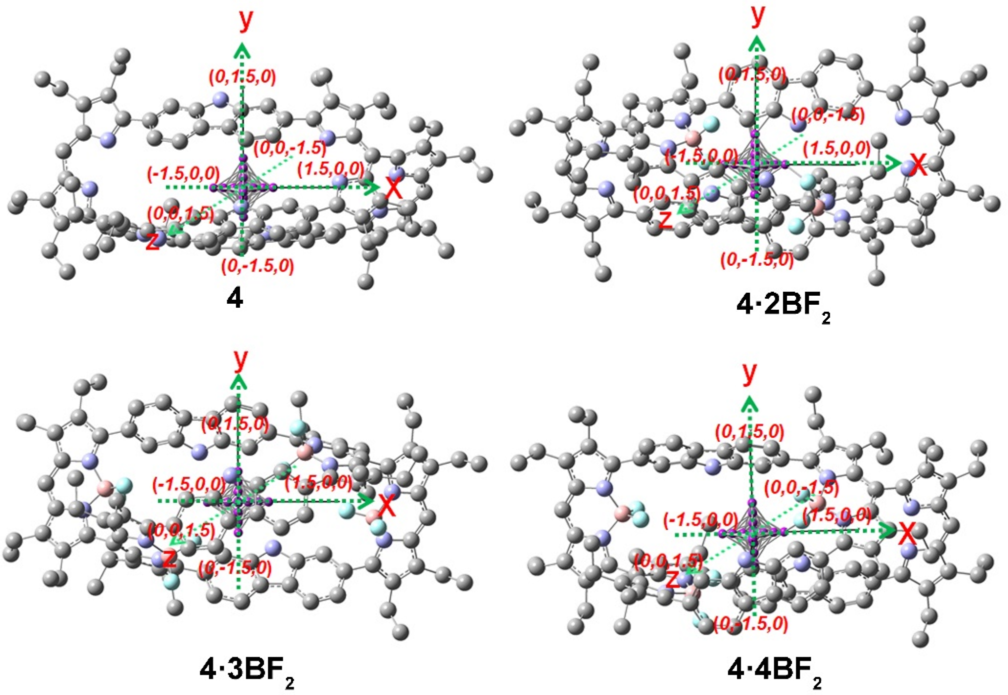

Figure S63. NICS values calculated at selected positions of **4** and the corresponding of BODIPY arrays.

Table S4. Calculated NICS values along the x, y and z axes referenced to the geometric center.

Bq Coordinates	4	4·2BF$_2$	4·3BF$_2$	4·4BF$_2$
**(0, 0, 0)**	**-1.78**	**1.32**	**-0.62**	**-2.11**
(0, 0.5, 0)	-2.13	0.64	-0.92	-2.14
(0, 1, 0)	-3.04	-3.57	-1.50	-2.54
(0, 1.5, 0)	-4.93	-10.28	-2.78	-3.73
(0, -0.5, 0)	-1.90	0.67	-0.90	-2.30
(0, -1, 0)	-2.70	-0.53	-2.54	-2.80
(0, -1.5, 0)	-5.16	-1.23	-4.47	-3.93
(0.5, 0, 0)	-1.72	1.30	-0.43	-2.11
(1, 0, 0)	-1.62	1.33	-0.34	-2.08
(1.5, 0, 0)	-1.49	1.08	-0.70	-2.04
(-0.5, 0, 0)	-1.76	1.29	-0.78	-2.08
(-1, 0, 0)	-1.69	1.22	-0.89	-2.00
(-1.5, 0, 0)	-1.56	0.77	-1.05	-1.92
(0, 0, 0.5)	-0.92	1.26	-1.00	-2.66
(0, 0, 1)	-1.87	0.81	-1.49	-3.12
(0, 0, 1.5)	-11.01	0.17	-1.95	-3.61
(0, 0, -0.5)	-2.63	0.86	-0.46	-1.45
(0, 0, -1)	-3.15	0.01	-0.45	-0.69
(0, 0, -1.5)	-3.29	-1.07	-0.55	0.05

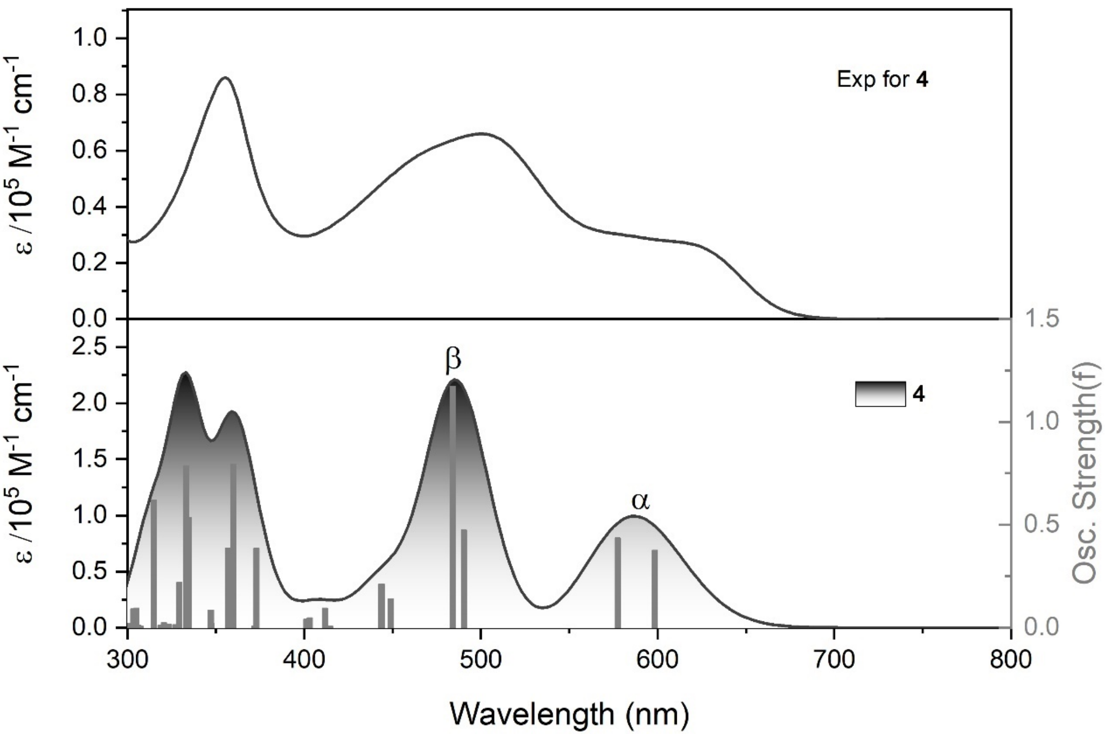

Figure S64. Comparison between the steady-state absorption (top) and TDDFT-predicted (bottom) spectra of **4** calculated at the CAM-B3LYP/6-311G(d,p) level.

Table S5. Selected TD-DFT calculated excitation state energies, oscillator strengths and compositions of the major electronic transitions of **4** calculated at the CAM-B3LYP/6-311G(d,p) level.

Peak	Sn	Wavelength [nm]	Oscillator Strength (f)	Energies [eV]	Major contributions
α	1	599.51	0.3740	2.3015	H→L (58.8%), H-1→L+1 (19.7%)
	2	576.63	0.4349	2.3754	H→L+1 (44.6%), H-1→L (30.8%)
β	3	490.94	0.4728	2.7412	H-2→L+1 (26.7%), H→L+3 (15.0%), H-2→L+2 (12.2%)
	4	484.12	1.1720	2.7722	H-3→L+2 (25.5%), H-2→L (14.1%), H-3→L (12.0%)

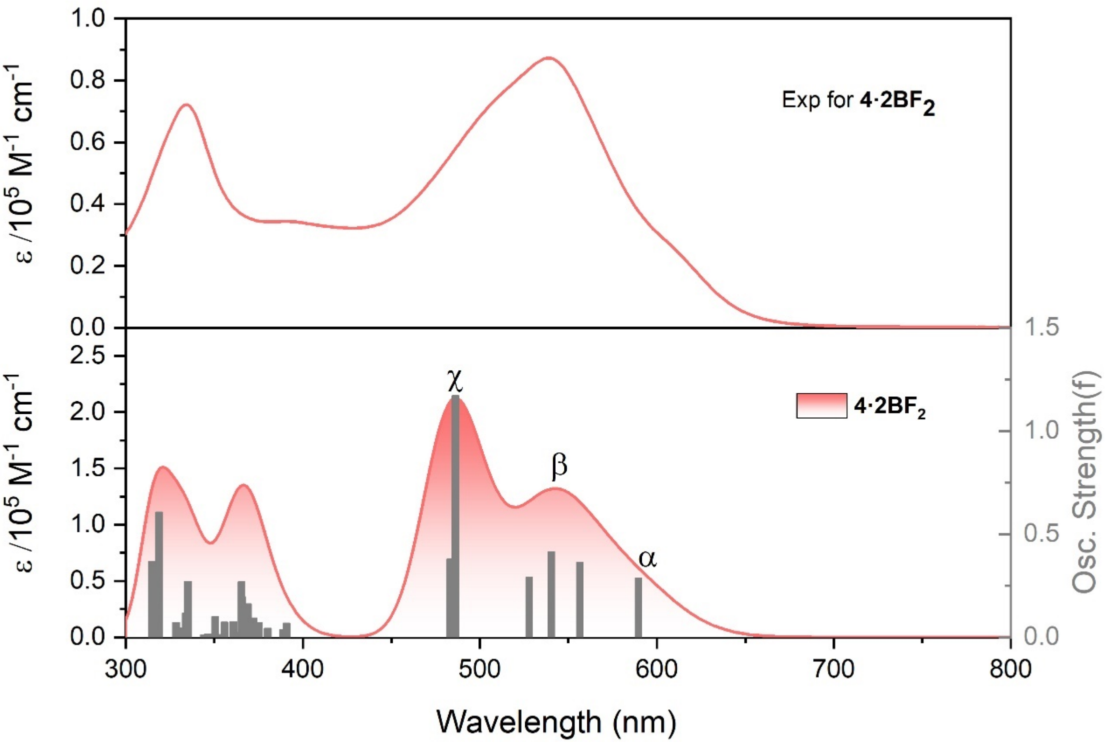

Figure S65. Comparison between the steady-state absorption (top) and TDDFT-predicted (bottom) spectra of **4·2BF$_2$** calculated at the CAM-B3LYP/6-311G(d,p) level.

Table S6. Selected TD-DFT calculated excitation state energies, oscillator strengths and compositions of the major electronic transitions of **4·2BF$_2$** calculated at the CAM-B3LYP/6-311G(d,p) level.

Peak	Sn	Wavelength [nm]	Oscillator Strength (f)	Energies [eV]	Major contributions
α	1	589.19	0.2866	2.3317	H→L (47.4%), H-2→L+1 (19.2%)
β	2	556.28	0.3643	2.4542	H-1→L (35.6%), H-3→L+1 (19.6%)
	3	540.31	0.4147	2.5186	H→L+1 (39.4%), H-2→L (17.1%), H-1→L (15.0%)
	4	528.43	0.2899	2.5706	H-1→L+1 (41.5%), H-3→L (20.0%), H-2→L (11.9%)
χ	5	486.46	1.1717	2.7613	H-1→L+2 (29.1%), H→L+3 (27.2%),
	6	483.54	0.3794	2.7760	H-1→L+3 (31.7%), H→L+2 (19.0%), H-3→L+1 (12.2%)

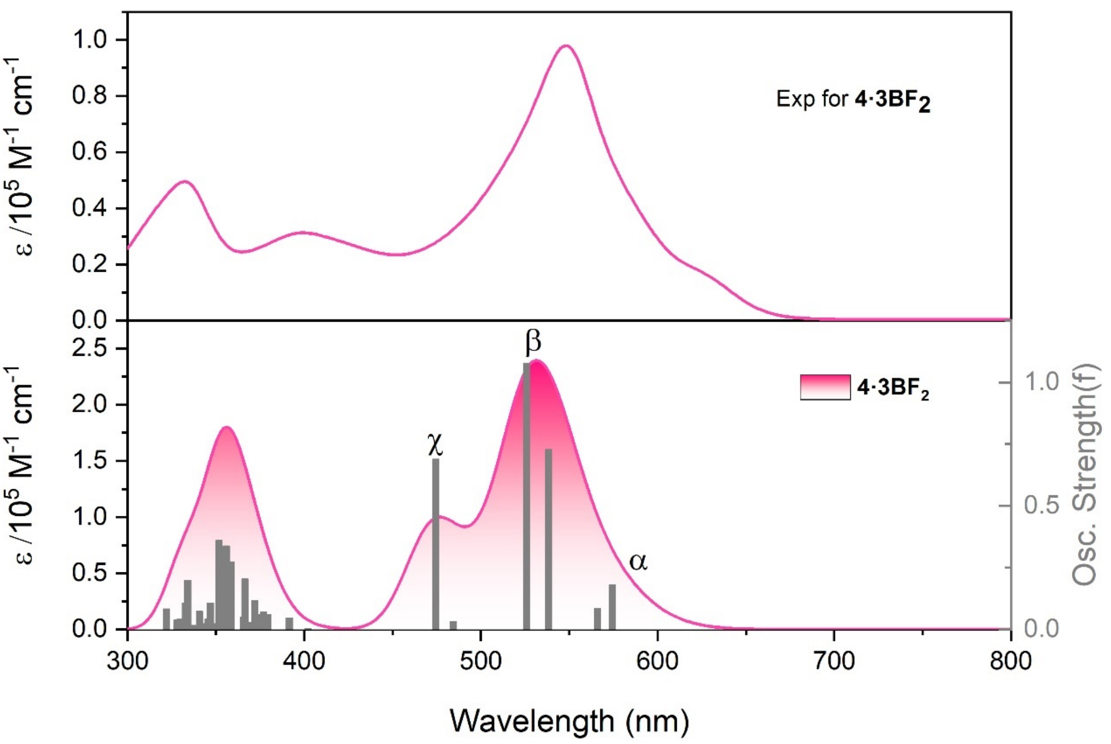

Figure S66. Comparison between the steady-state absorption (top) and TDDFT-predicted (bottom) spectra of **4·3BF₂** calculated at the CAM-B3LYP/6-311G(d,p) level.

Table S7. Selected TD-DFT calculated excitation state energies, oscillator strengths and compositions of the major electronic transitions of **4·3BF₂** calculated at the CAM-B3LYP/6-311G(d,p) level.

Peak	Sn	Wavelength [nm]	Oscillator Strength (f)	Energies [eV]	Major contributions
α	1	575.07	0.1800	2.3863	H-1→L+1 (24.7%), H-2→L+1 (12.8%), H→L+1 (11.8%)
β	3	537.39	0.7276	2.5266	H-3→L (28.7%), H-1→L+1 (18.3%), H→L+2 (12.4%), H-2→L (10.6%)
	4	525.51	1.0769	2.5792	H-3→L (23.9%), H→L+2 (22.9%), H-2→L+1 (16.7%)
χ	6	473.22	0.6881	2.8209	H→L+3 (41.5%), H-1→L+3 (15.3%), H-2→L+3 (10.6%)

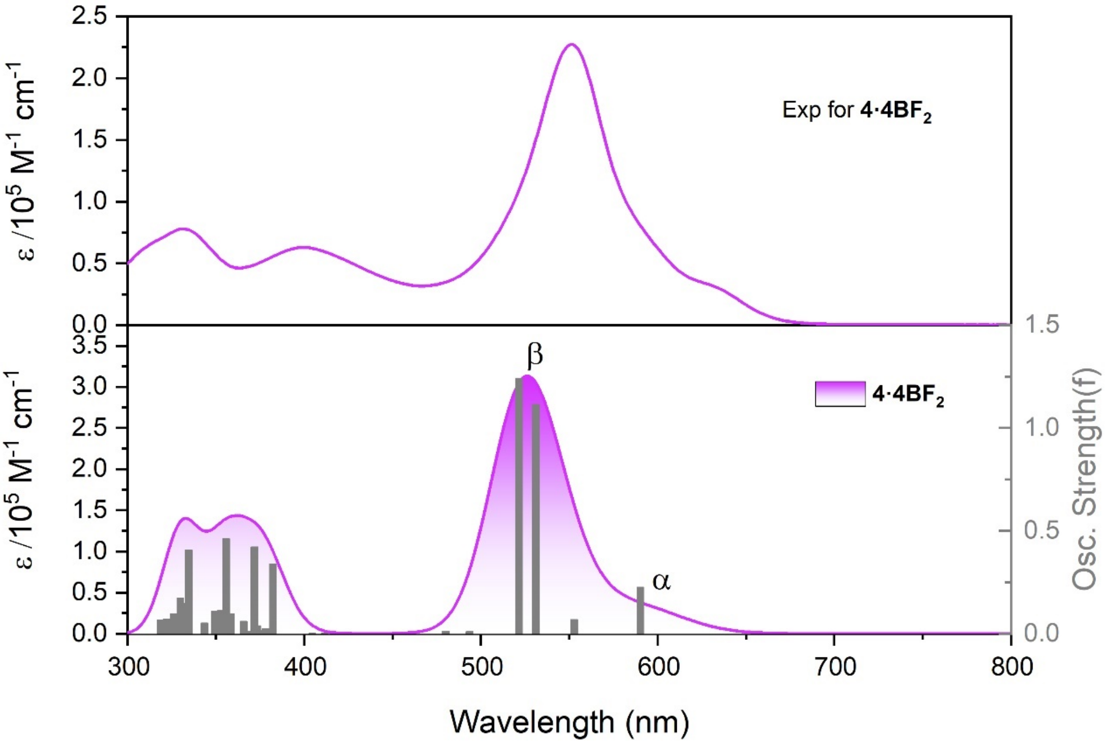

Figure S67. Comparison between the steady-state absorption (top) and TDDFT-predicted (bottom) spectra of **4·4BF₂** calculated at the CAM-B3LYP/6-311G(d,p) level.

Table S8. Selected TD-DFT calculated excitation state energies, oscillator strengths and compositions of the major electronic transitions of **4·4BF₂** calculated at the CAM-B3LYP/6-311G(d,p) level.

Peak	Sn	Wavelength [nm]	Oscillator Strength (f)	Energies [eV]	Major contributions
α	1	590.45	0.2258	2.3295	H→L+2 (60.9%), H-1→L+3 (10.6%)
β	3	530.57	1.1146	2.5576	H-2→L (26.4%), H-3→L (22.6%), H→L+3 (10.5%)
	4	520.35	1.2404	2.5984	H-1→L+3 (22.4%), H-2→L+1 (21.1%), H-3→L+1 (20.7%)

8. Supporting references

1 a) G. M. Sheldrick, *Methods. Enzymol.,* **1997**, *277*, 319; b) A. Altomare, M. C. Burla, M. Camalli, G. L. Cascarano, C. Giacovazzo, A. Guagliardi, A. G. G. Moliterni, G. Polidori and R. Spagna, *J. Appl. Cryst.,* 1999, **32***,* 115−119.

2 W. N. Zhou. M. D. Hao, T. Lu, Z. M. Duan, T. Sarma, J. L. Sessler and C. H. Lei, *Chem.-Eur. J.,* 2021, **27**, 16173-16180.

3 S. V. Shevchuk, J. M. Davis and J. L. Sessler, *Tetrahedron Lett.,* 2001, **42**, 2447−2450.

4 Z. Y. Zhu, X. Zhang, X. Guo, Q. H. Wu, Z. X. Li, C. J. Yu, E. H. Hao, L. J. Jiao and J. Z. Zhao, *Chem. Sci.*, 2021, **12**, 14944–14951.

5 M. J. F. Gaussian 16 Revision A.03, G. W. Trucks, H. B. Schlegel, G. E. Scuseria, M. A. Robb, J. R. Cheeseman, G. Scalmani, V. Barone, G. A. Petersson, H. Nakatsuji, X. Li, M. Caricato, A. V. Marenich, J. Bloino, B. G. Janesko, R. Gomperts, B. Mennucci, H. P. Hratchian, J. V. Ortiz, A. F. Izmaylov, J. L. Sonnenberg, D. Williams, F. Ding, F. Lipparini, F. Egidi, J. Goings, B. Peng, A. Petrone, T. Henderson, D. Ranasinghe, V. G. Zakrzewski, J. Gao, N. Rega, G. Zheng, W. Liang, M. Hada, M. Ehara, K. Toyota, R. Fukuda, J. Hasegawa, M. Ishida, T. Nakajima, Y. Honda, O. Kitao, H. Nakai, T. Vreven, K. Throssell, J. A. Montgomery Jr., J. E. Peralta, F. Ogliaro, M. J. Bearpark, J. J. Heyd, E. N. Brothers, K. N. Kudin, V. N. Staroverov, T. A. Keith, R. Kobayashi, J. Normand, K. Raghavachari, A. P. Rendell, J. C. Burant, S. S. Iyengar, J. Tomasi, M. Cossi, J. M. Millam, M. Klene, C. Adamo, R. Cammi, J. W. Ochterski, R. L. Martin, K. Morokuma, O. Farkas, J. B. Foresman, and D. J. Fox, Gaussian Inc., Wallingford, CT, **2016**.

6 a) A. D. Becke, *J. Chem. Phys*., 1993, **98**, 1372; b) C. Lee, W. Yang and R. G. Parr, *Phys. Rev. B*., 1988, **37**, 785.

7 a) Z. Chen, C. S. Wannere, C. Corminboeuf, R. Puchta, P. v. R. Schleyer, Chem Rev, 2005, 105, 3842−3888; b) P. V. R. Schleyer, C. Maerker, A. Dransfeld, H. Jiao, N. J. R. van Eikema Hommes, J. Am. Chem. Soc. 1996, 118, 6317-6318.

8 T. Yanai, D. P. Tew and N. C. Handy, *Chem. Phys. Lett.,* 2004, **393**, 51– 56.

9 B. Mennucci, E. Cancès and J. Tomasi, *J. Phys., Chem. B.,* 1997, **101**, 10506–10517.

Cartesian coordinates of optimized geometries and minimized energies

Table S8: Cartesian coordinates of the S_0 optimized geometry of **4** optimized at B3LYP/6-311G(d,p) level of theory.

* Sum of imaginary frequencies = 0

*Total Energy (hartree) = -4631.614881 Hartree

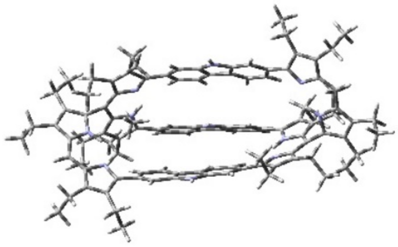

Optimized structure Coordinates:

Atom	X	Y	Z
N	6.854346	-1.466258	-1.982129
N	7.736724	0.56643	-0.590037
H	6.945755	-0.039277	-0.838286
N	4.896844	0.639109	2.466633
H	4.204034	1.317247	2.115454
N	-0.079574	-0.628512	2.429506
H	-0.112809	-0.359203	1.459721
N	-4.970429	-2.041104	1.975617
H	-4.272862	-2.34615	1.279858
N	-7.737608	-0.163705	-0.740598
H	-6.92792	0.465086	-0.700992
N	-6.809299	2.277316	-1.044598
N	-1.13201	4.284864	-1.258377
H	-1.0049	4.763855	-2.133179
N	4.265068	2.654399	1.01355
N	1.153031	-2.827692	-3.223873
H	1.037361	-2.749471	-4.219555
N	-4.289075	-2.650389	-0.425173
C	6.098818	-2.334147	-2.622293
C	6.829486	-2.968243	-3.724545
C	8.090092	-2.419927	-3.705306
C	8.100793	-1.454065	-2.615073
C	6.331713	-4.08247	-4.596295
H	5.398827	-3.796106	-5.092429
H	7.054927	-4.26131	-5.396817
C	6.101024	-5.388423	-3.817144
H	5.357094	-5.246823	-3.030641
H	7.028246	-5.721571	-3.344317
H	5.748748	-6.18335	-4.480274
C	9.268405	-2.770872	-4.561033
H	9.860163	-1.871153	-4.758992
H	8.924856	-3.126866	-5.536337
C	10.168473	-3.839675	-3.916661
H	10.535956	-3.50231	-2.944495
H	11.030375	-4.063974	-4.551179

H	9.611424	-4.765636	-3.755418
C	9.094738	-0.58157	-2.233802
H	10.046477	-0.621459	-2.751499
C	8.948423	0.39701	-1.217001
C	9.826156	1.343748	-0.649295
C	9.098524	2.071075	0.330269
C	7.791162	1.558895	0.326501
C	11.284051	1.490303	-0.960953
H	11.465522	1.233183	-2.009247
H	11.579389	2.537876	-0.849325
C	12.169738	0.609434	-0.062025
H	12.022123	0.856174	0.992254
H	13.229097	0.741967	-0.298374
H	11.916752	-0.445648	-0.19133
C	9.66444	3.160688	1.199307
H	10.651782	2.846122	1.554297
H	9.848316	4.056016	0.590035
C	8.801212	3.543127	2.404614
H	7.836477	3.949899	2.097078
H	9.306577	4.299109	3.010082
H	8.603471	2.67618	3.03942
C	6.514212	1.764378	1.053207
C	6.186698	0.747463	2.006654
C	6.923944	-0.297713	2.641478
C	6.023433	-0.992134	3.480171
C	4.753818	-0.394495	3.32259
C	8.361619	-0.689739	2.447359
H	8.799517	-0.922802	3.423615
H	8.931446	0.146845	2.046006
C	8.532616	-1.907572	1.519319
H	8.082393	-1.725109	0.544385
H	8.058479	-2.796643	1.941415
H	9.59271	-2.128741	1.368653
C	6.344844	-2.149745	4.379646
H	7.110912	-2.777388	3.918056
H	5.458202	-2.781291	4.487556
C	6.832442	-1.709106	5.770359
H	6.08019	-1.097085	6.274578
H	7.740116	-1.105769	5.690635
H	7.051751	-2.573122	6.403975
C	3.40039	-0.752433	3.737349
C	3.077025	-1.268175	5.012358
H	3.867626	-1.429995	5.732406
C	1.759769	-1.522163	5.374865

H	1.536909	-1.902476	6.36507
C	0.727428	-1.274036	4.463164
C	-0.714895	-1.423877	4.471647
C	-1.66939	-1.837698	5.408525
H	-1.365108	-2.135351	6.405333
C	-3.012153	-1.889836	5.051018
H	-3.742629	-2.247335	5.765047
C	-3.434536	-1.542635	3.749649
C	-2.495419	-1.086411	2.812053
H	-2.807547	-0.776001	1.823786
C	-1.164587	-1.031727	3.180891
C	1.07225	-0.777591	3.175489
C	2.378688	-0.518797	2.80425
H	2.608362	-0.162829	1.808822
C	-4.806926	-1.669276	3.261752
C	-6.071108	-1.278078	3.752899
C	-6.987458	-1.372857	2.681198
C	-6.263242	-1.854411	1.549585
C	-6.363969	-0.819035	5.150682
H	-7.131846	-0.040753	5.132221
H	-5.467187	-0.353735	5.570796
C	-6.825848	-1.960206	6.072258
H	-7.027113	-1.595082	7.083311
H	-6.064911	-2.742334	6.136104
H	-7.737886	-2.424924	5.689196
C	-8.420017	-0.929083	2.763033
H	-8.812	-1.186756	3.751861
H	-9.029201	-1.466427	2.038279
C	-8.58845	0.584014	2.533172
H	-9.641258	0.870584	2.602757
H	-8.223432	0.870995	1.547245
H	-8.0288	1.15731	3.276385
C	-6.562341	-2.026313	0.163367
C	-7.83681	-1.447618	-0.325811
C	-9.164066	-1.862179	-0.506804
C	-9.864002	-0.757596	-1.064947
C	-8.946913	0.305807	-1.196812
C	-9.777363	-3.19786	-0.18419
H	-10.698501	-3.027812	0.386986
H	-10.100791	-3.680562	-1.113975
C	-8.883188	-4.165737	0.594924
H	-8.586783	-3.751367	1.560115
H	-7.967874	-4.398944	0.04746
H	-9.412199	-5.10384	0.778253

C	-11.298323	-0.748398	-1.495463
H	-11.683467	0.275535	-1.481161
H	-11.900396	-1.310255	-0.773895
C	-11.497046	-1.348938	-2.898295
H	-12.552533	-1.344663	-3.183923
H	-11.138689	-2.380304	-2.93524
H	-10.936371	-0.777884	-3.642384
C	-9.069201	1.64589	-1.648754
H	-10.024902	1.943818	-2.064719
C	-8.057186	2.576612	-1.597046
C	-8.046571	3.949332	-2.083965
C	-6.787937	4.440193	-1.831909
C	-6.055757	3.349867	-1.17546
C	-9.225286	4.671012	-2.66221
H	-8.882505	5.44293	-3.356998
H	-9.826625	3.97568	-3.256816
C	-10.111984	5.315344	-1.582325
H	-10.974152	5.81854	-2.028732
H	-9.545158	6.051702	-1.007748
H	-10.478874	4.561699	-0.881352
C	-6.301833	5.842096	-2.052635
H	-5.373811	5.842981	-2.633347
H	-7.034053	6.38085	-2.660405
C	-6.071616	6.608367	-0.738998
H	-6.996779	6.661448	-0.159802
H	-5.730905	7.628701	-0.93558
H	-5.320077	6.111343	-0.122652
C	-4.686752	3.333873	-0.639331
C	-4.446798	2.621791	0.560973
H	-5.288733	2.150214	1.049492
C	-3.179201	2.530546	1.109331
H	-3.022924	1.988091	2.034966
C	-2.103513	3.134756	0.448133
C	-0.681401	3.194941	0.686295
C	0.165718	2.719189	1.695383
H	-0.239482	2.206751	2.559523
C	1.530685	2.902135	1.575924
H	2.189889	2.538393	2.350452
C	2.108894	3.548407	0.45092
C	1.265438	4.069543	-0.539951
H	1.666061	4.548683	-1.419852
C	-0.108341	3.895091	-0.406524
C	-2.346464	3.828012	-0.768513
C	-3.623356	3.950963	-1.307098

H	-3.783751	4.465887	-2.245418
C	3.571075	3.534719	0.312281
C	4.453027	4.29649	-0.569673
C	5.718337	3.773002	-0.401298
C	5.592381	2.71164	0.598712
C	4.081943	5.46931	-1.432498
H	3.213237	5.973597	-1.001749
H	4.89343	6.20345	-1.402984
C	3.802322	5.117305	-2.904576
H	3.455267	5.994864	-3.458037
H	3.046695	4.332865	-2.994652
H	4.705736	4.747601	-3.391691
C	6.955503	4.258945	-1.099008
H	6.820661	5.317727	-1.342735
H	7.808974	4.209122	-0.424142
C	7.295929	3.495929	-2.391243
H	6.487806	3.584283	-3.119889
H	7.452344	2.436328	-2.197857
H	8.207729	3.896709	-2.842395
C	-5.611962	-2.416126	-0.786253
C	-5.715845	-2.512305	-2.243123
C	-4.446265	-2.783773	-2.70392
C	-3.577628	-2.831204	-1.527567
C	-6.937234	-2.413799	-3.112834
H	-7.60143	-1.626727	-2.760854
H	-6.629374	-2.1187	-4.119401
C	-7.713763	-3.73748	-3.198081
H	-8.025485	-4.069206	-2.207603
H	-7.093029	-4.526341	-3.63083
H	-8.608461	-3.624704	-3.816816
C	-4.078666	-2.967558	-4.149627
H	-4.932735	-3.395025	-4.681645
H	-3.280498	-3.708005	-4.244419
C	-3.675472	-1.653833	-4.842713
H	-4.507112	-0.94562	-4.825376
H	-3.394016	-1.825575	-5.885971
H	-2.836359	-1.173897	-4.333492
C	-2.117085	-2.956358	-1.413952
C	-1.257291	-2.892401	-2.520365
H	-1.639715	-2.805922	-3.524578
C	0.116885	-2.895888	-2.30316
C	2.367951	-2.780094	-2.556592
C	3.657414	-2.66951	-3.067248
H	3.831665	-2.609797	-4.13406

C	4.719447	-2.557752	-2.16368
C	4.46829	-2.580011	-0.77074
H	5.308939	-2.475678	-0.097811
C	3.186981	-2.732327	-0.268646
H	3.019575	-2.75283	0.802126
C	2.111898	-2.833981	-1.159486
C	0.680677	-2.932794	-1.002143
C	-0.182582	-3.043272	0.095197
H	0.211681	-3.1127	1.101783
C	-1.548388	-3.063453	-0.115642
H	-2.21496	-3.157628	0.727876

Table S9: Cartesian coordinates of the S_0 optimized geometry of **4·2BF$_2$** optimized at B3LYP/6-311G(d,p) level of theory.

* Sum of imaginary frequencies = 0

*Total Energy (hartree) = -5080.025206 Hartree

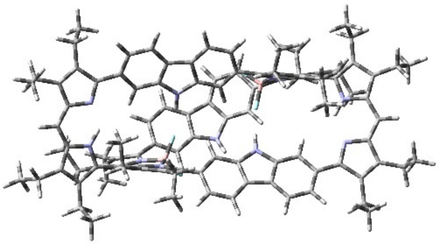

Optimized structure Coordinates:

Symbol	X	Y	Z
F	2.066831	-2.992965	0.675525
F	2.628849	-0.765155	0.8903
F	-2.424862	0.27388	1.250431
F	-2.190321	2.297005	2.34265
N	4.310858	-2.462527	1.402696
N	3.589823	-1.985307	-0.933721
N	-2.110776	-1.612961	-1.094335
H	-2.198271	-1.001379	-0.290535
N	-7.314199	-1.496237	-1.728363
N	-7.615822	0.686703	-0.344838
H	-7.013275	-0.086412	-0.655217
N	-4.319615	1.122532	2.529294
N	-3.579525	2.207492	0.389764
N	2.134171	2.032481	0.107673
H	2.233051	1.046082	0.321115
N	7.329062	2.333959	-0.358096
N	7.624841	-0.247479	-0.537578
H	7.019337	0.559718	-0.341377
N	0.509477	-0.18601	4.378811
H	0.81015	0.712681	4.035948
C	4.282822	-2.822045	2.706014
C	5.596743	-3.043738	3.167805
C	6.464408	-2.765664	2.106346
C	5.64307	-2.394582	0.991739
C	5.948061	-3.484002	4.557521
H	5.207789	-3.077335	5.25273
H	6.91298	-3.059794	4.84838
C	5.992818	-5.013426	4.70576
H	5.018053	-5.45012	4.476343
H	6.262907	-5.302057	5.725146
H	6.723148	-5.451813	4.020557
C	7.960438	-2.803218	2.225939
H	8.417423	-3.138809	1.298635
H	8.221777	-3.543537	2.987084
C	8.56235	-1.44347	2.621828
H	8.154204	-1.101825	3.576303

H	8.342973	-0.684728	1.871103
H	9.648218	-1.519887	2.72014
C	5.934517	-1.938859	-0.302867
C	4.923393	-1.693565	-1.240826
C	4.986335	-1.320658	-2.622771
C	3.69574	-1.456469	-3.133288
C	2.849784	-1.868998	-2.061792
C	6.153836	-0.778286	-3.39601
H	6.092097	-1.136988	-4.426078
H	7.093891	-1.153159	-2.996069
C	6.178579	0.762502	-3.405719
H	5.260672	1.167933	-3.837221
H	7.02257	1.122181	-4.000047
H	6.28243	1.171461	-2.400827
C	3.268754	-1.15582	-4.54404
H	3.695183	-0.193172	-4.841849
H	2.184656	-1.030424	-4.570372
C	3.682155	-2.216005	-5.578982
H	3.22753	-3.184403	-5.362387
H	4.764415	-2.360347	-5.595022
H	3.367811	-1.915633	-6.581999
C	1.420046	-2.200649	-2.192599
C	1.105356	-3.108277	-3.230966
H	1.913646	-3.546954	-3.797486
C	-0.197839	-3.474463	-3.500077
H	-0.419009	-4.181189	-4.291326
C	-1.222576	-2.91763	-2.73687
C	-2.651042	-3.047638	-2.781619
C	-3.518218	-3.761262	-3.607953
H	-3.129338	-4.388556	-4.401704
C	-4.882363	-3.64282	-3.41754
H	-5.548589	-4.164137	-4.085785
C	-5.417608	-2.811031	-2.407419
C	-4.548373	-2.099298	-1.55828
H	-4.952734	-1.461143	-0.784516
C	-3.175132	-2.21919	-1.750983
C	-0.916046	-2.015916	-1.680064
C	0.403789	-1.656483	-1.396234
H	0.642373	-0.986041	-0.582874
C	-6.867321	-2.589765	-2.316432
C	-7.987817	-3.375079	-2.875055
C	-9.123679	-2.650884	-2.609536
C	-8.698729	-1.456933	-1.896611
C	-7.95509	-4.73432	-3.513679

H	-7.366139	-4.720794	-4.436275
H	-8.969995	-4.992145	-3.82748
C	-7.429231	-5.834026	-2.577327
H	-8.05295	-5.899658	-1.682381
H	-6.406621	-5.629207	-2.256273
H	-7.440786	-6.807586	-3.074956
C	-10.537556	-2.963665	-2.99561
H	-10.691907	-4.046239	-2.997806
H	-11.22066	-2.564947	-2.239292
C	-10.91193	-2.3927	-4.374371
H	-11.951754	-2.619104	-4.62579
H	-10.27087	-2.814003	-5.152361
H	-10.782396	-1.307782	-4.393093
C	-9.459248	-0.39662	-1.461546
H	-10.520831	-0.394284	-1.678837
C	-8.934454	0.704028	-0.73383
C	-9.48789	1.90234	-0.23427
C	-8.456068	2.586292	0.461759
C	-7.310388	1.786871	0.372482
C	-10.920481	2.328344	-0.342267
H	-11.357622	1.928737	-1.2622
H	-10.974186	3.417693	-0.429405
C	-11.759479	1.870932	0.863733
H	-12.798146	2.199604	0.770619
H	-11.354683	2.276756	1.794172
H	-11.748713	0.781599	0.948286
C	-8.526508	3.940398	1.104945
H	-9.535353	4.113971	1.491421
H	-7.857221	3.966603	1.970713
C	-8.141433	5.077132	0.143689
H	-8.222708	6.050955	0.633996
H	-8.790647	5.081189	-0.735672
H	-7.112555	4.954894	-0.202502
C	-5.925566	1.868116	0.874715
C	-5.642101	1.412353	2.169419
C	-6.499401	0.940985	3.216712
C	-5.680262	0.32421	4.159218
C	-4.345281	0.415555	3.691075
C	-7.993159	1.025563	3.346643
H	-8.378797	1.900532	2.831403
H	-8.231905	1.159545	4.405859
C	-8.715482	-0.229892	2.827418
H	-9.795723	-0.131476	2.960694
H	-8.38331	-1.120732	3.366095

H	-8.517565	-0.385603	1.767661
C	-6.09996	-0.226075	5.49197
H	-5.476056	-1.081019	5.757517
H	-7.125845	-0.598138	5.432386
C	-5.994483	0.827888	6.607215
H	-6.312081	0.415636	7.568785
H	-6.617333	1.698037	6.384263
H	-4.963087	1.173996	6.707845
C	-3.181413	-0.316151	4.200072
C	-3.31303	-1.718801	4.345115
H	-4.288869	-2.168317	4.21717
C	-2.204877	-2.531167	4.51573
H	-2.320024	-3.607946	4.554741
C	-0.934578	-1.948771	4.52585
C	0.405557	-2.469849	4.368481
C	0.930245	-3.751514	4.177111
H	0.304612	-4.627014	4.304514
C	2.235257	-3.887022	3.726337
H	2.625563	-4.865525	3.477756
C	3.02206	-2.751499	3.46469
C	2.556202	-1.471669	3.757421
H	3.157123	-0.604333	3.519225
C	1.251452	-1.343505	4.202541
C	-0.829834	-0.535675	4.466095
C	-1.934725	0.289514	4.33779
H	-1.823	1.354016	4.207763
C	-4.909703	2.206057	-0.029505
C	-4.95851	2.749527	-1.352812
C	-3.655551	3.136009	-1.672972
C	-2.822689	2.79577	-0.570115
C	-6.121246	2.809916	-2.301182
H	-6.039237	3.717112	-2.904407
H	-7.062404	2.884902	-1.760381
C	-6.165699	1.588985	-3.240481
H	-6.280441	0.657832	-2.685738
H	-5.250106	1.513667	-3.831379
H	-7.010152	1.673378	-3.929539
C	-3.205743	3.740418	-2.975162
H	-3.631641	3.158181	-3.798018
H	-2.121862	3.639639	-3.059403
C	-3.593023	5.216564	-3.167046
H	-4.673108	5.360802	-3.097132
H	-3.268814	5.573374	-4.148245
H	-3.128987	5.852135	-2.410766

C	-1.395485	3.14402	-0.447602
C	-1.089176	4.50532	-0.680337
H	-1.90177	5.193876	-0.860442
C	0.209152	4.972781	-0.637584
H	0.422138	6.02169	-0.807775
C	1.23906	4.071454	-0.373213
C	2.666635	4.211468	-0.302477
C	3.534012	5.287449	-0.487308
H	3.146167	6.272632	-0.719301
C	4.898893	5.079722	-0.397771
H	5.561708	5.907493	-0.58782
C	5.435301	3.799925	-0.124959
C	4.565054	2.712523	0.08291
H	4.967209	1.730406	0.289605
C	3.193702	2.922976	-0.010857
C	0.938917	2.703639	-0.122032
C	-0.375429	2.231248	-0.15208
H	-0.608632	1.195959	0.0511
C	6.883875	3.562704	-0.172146
C	8.006091	4.518816	-0.108667
C	9.144332	3.779254	-0.316788
C	8.717273	2.398632	-0.478652
C	7.966074	5.994107	0.174149
H	7.087044	6.233148	0.77599
H	8.829089	6.253475	0.795942
C	7.996241	6.872656	-1.08873
H	7.177294	6.626746	-1.768928
H	8.928336	6.723515	-1.638011
H	7.920506	7.932729	-0.831118
C	10.561266	4.259214	-0.389619
H	10.645695	5.228713	0.109115
H	11.211125	3.573272	0.164369
C	11.077893	4.387305	-1.834273
H	11.03965	3.425893	-2.351653
H	12.111527	4.743302	-1.849916
H	10.464646	5.089003	-2.403687
C	9.481196	1.283665	-0.733912
H	10.548898	1.405259	-0.872885
C	8.953612	-0.031692	-0.819698
C	9.513934	-1.291381	-1.121146
C	8.476682	-2.253795	-1.004553
C	7.320565	-1.558062	-0.631847
C	10.955573	-1.569717	-1.420535
H	11.029491	-2.387223	-2.143964

H	11.409272	-0.697754	-1.900994
C	11.755704	-1.93205	-0.157113
H	11.335952	-2.816895	0.327762
H	11.722477	-1.113958	0.56648
H	12.8023	-2.138061	-0.397276
C	8.552421	-3.723499	-1.298521
H	9.554686	-4.095506	-1.065702
H	7.864008	-4.261701	-0.639418
C	8.206989	-4.056884	-2.759338
H	7.184621	-3.750025	-2.991705
H	8.291544	-5.130468	-2.947934
H	8.876404	-3.534489	-3.447639
B	3.086657	-2.081228	0.525883
B	-3.078671	1.475684	1.652821

Table S10: Cartesian coordinates of the S_0 optimized geometry of **4·3BF$_2$** optimized at B3LYP/6-311G(d,p) level of theory.

* Sum of imaginary frequencies = 0

*Total Energy (hartree) = -5304.220428 Hartree

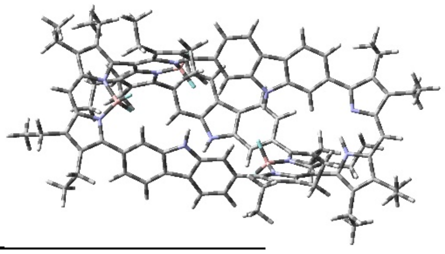

Optimized structure Coordinates:

Symbol	X	Y	Z
F	-2.685272	-4.143141	0.693805
F	-2.003595	-1.975032	1.085181
F	-5.711547	0.861979	-2.279206
F	-5.451812	0.833145	0.013684
F	2.507565	2.379902	2.525705
F	2.425278	0.733871	0.904794
N	-3.426703	-2.418105	-0.806539
N	-4.315239	-2.541403	1.52832
N	-7.377475	-0.258216	-0.962923
N	-7.193618	2.23436	-0.949299
N	-1.896845	3.017021	-1.531836
H	-1.88922	2.331248	-2.268964
N	3.730422	2.729376	0.492267
N	4.484644	1.016307	2.164784
N	7.746583	0.917153	-0.506113
N	7.517642	-1.469977	-1.530663
N	2.309005	-1.787357	-0.834244
H	2.340781	-0.983382	-0.217653
N	-0.260498	-0.378106	4.480563
H	-0.548608	0.57396	4.319787
C	-2.599343	-2.42434	-1.869916
C	-3.353764	-2.159036	-3.050114
C	-4.684737	-2.005156	-2.672984
C	-4.726441	-2.160822	-1.248972
C	-5.764879	-2.005747	-0.327576
C	-5.567354	-2.16055	1.051462
C	-6.400693	-1.871829	2.182835
C	-5.617467	-2.084869	3.320448
C	-4.336564	-2.48731	2.879139
C	-2.774209	-1.899841	-4.409843
H	-1.951213	-2.587826	-4.611867
H	-3.531121	-2.083384	-5.176391
C	-2.259751	-0.453724	-4.52687
H	-1.861469	-0.251414	-5.525023
H	-1.46112	-0.281569	-3.799881
H	-3.066574	0.25555	-4.322122

C	-5.818326	-1.789661	-3.631878
H	-5.460992	-1.169199	-4.457609
H	-6.621007	-1.225206	-3.168917
C	-6.367841	-3.11307	-4.189333
H	-5.589058	-3.675442	-4.710417
H	-7.184657	-2.925843	-4.892115
H	-6.750353	-3.744631	-3.385563
C	-7.793849	-1.310927	2.2186
H	-8.339966	-1.779731	3.043731
H	-8.340979	-1.561901	1.313592
C	-7.798835	0.21735	2.409471
H	-7.204642	0.703059	1.63738
H	-7.368405	0.491249	3.375687
H	-8.819711	0.607278	2.366564
C	-6.026169	-1.977639	4.759689
H	-6.874149	-1.295144	4.855751
H	-5.207446	-1.539091	5.337996
C	-6.390615	-3.342609	5.366342
H	-5.537905	-4.023749	5.314326
H	-7.218429	-3.801541	4.819208
H	-6.684568	-3.243312	6.415025
C	-3.073082	-2.633817	3.621427
C	-2.342172	-3.835703	3.583457
H	-2.827425	-4.731963	3.223796
C	-0.981638	-3.842757	3.842271
H	-0.410646	-4.755515	3.721902
C	-0.340724	-2.634526	4.124791
C	1.03997	-2.211243	4.077639
C	2.231386	-2.850912	3.727155
H	2.257991	-3.923139	3.573812
C	3.358941	-2.085063	3.484043
H	4.257993	-2.562687	3.118854
C	3.3293	-0.674051	3.594001
C	2.169777	-0.028622	4.025762
H	2.131631	1.046759	4.091766
C	1.039747	-0.802211	4.239696
C	-1.115372	-1.45818	4.292127
C	-2.482893	-1.448483	4.064504
H	-3.040661	-0.520256	4.036277
C	4.513234	0.053181	3.122149
C	5.863862	-0.257482	3.437485
C	6.675728	0.489185	2.58793
C	5.800754	1.284518	1.771462
C	6.062534	2.129349	0.682371

C	5.040958	2.829217	0.0263
C	5.031575	3.645729	-1.147778
C	3.701181	4.020081	-1.363438
C	2.927606	3.445854	-0.32805
C	6.312541	-1.14411	4.566978
H	7.403385	-1.167207	4.585898
H	5.992536	-2.176425	4.40028
C	5.796702	-0.673238	5.935834
H	6.168572	-1.322453	6.732895
H	4.706183	-0.685047	5.970472
H	6.129411	0.347407	6.142019
C	8.175775	0.403281	2.577865
H	8.534705	0.536539	3.604756
H	8.607768	1.214666	1.999396
C	8.688878	-0.94498	2.039052
H	9.779515	-0.938123	1.970703
H	8.289678	-1.14954	1.046692
H	8.393644	-1.770854	2.689288
C	6.171199	4.015428	-2.051746
H	7.08029	4.187662	-1.480495
H	5.928928	4.961309	-2.543822
C	6.445403	2.9456	-3.123917
H	7.264655	3.258484	-3.77645
H	5.558911	2.778419	-3.740656
H	6.720969	1.9944	-2.667617
C	3.175925	4.950274	-2.41765
H	3.809884	4.893539	-3.306353
H	2.180316	4.624987	-2.728805
C	3.095506	6.405091	-1.927058
H	2.713445	7.0648	-2.710862
H	4.080928	6.767989	-1.623082
H	2.430256	6.480189	-1.063746
C	7.41553	2.074781	0.102235
C	8.540284	2.909974	0.099103
C	9.58589	2.18482	-0.53182
C	9.059172	0.929769	-0.907655
C	9.614777	-0.240898	-1.489061
H	10.674208	-0.233476	-1.716139
C	8.897261	-1.388117	-1.733044
C	9.385711	-2.674574	-2.203711
C	8.295812	-3.504004	-2.283336
C	7.132582	-2.687102	-1.869083
C	8.630664	4.258326	0.752548
H	9.338676	4.885837	0.202665

H	7.660281	4.759606	0.685032
C	9.051398	4.183331	2.22956
H	10.02035	3.688748	2.335857
H	8.321643	3.613026	2.808878
H	9.127613	5.182175	2.667509
C	10.973123	2.67977	-0.806156
H	11.670836	1.836613	-0.817278
H	11.29875	3.33269	0.009193
C	11.072489	3.440452	-2.139838
H	10.785076	2.794064	-2.972551
H	12.090522	3.798567	-2.316018
H	10.401092	4.302756	-2.14502
C	10.812764	-2.976183	-2.547946
H	11.476518	-2.401987	-1.894202
H	11.03056	-4.028401	-2.345693
C	11.144178	-2.660226	-4.016714
H	12.193511	-2.873316	-4.238585
H	10.952808	-1.607771	-4.239934
H	10.522862	-3.257369	-4.688344
C	8.349747	-4.968291	-2.615728
H	9.375953	-5.219273	-2.896255
H	7.749545	-5.194655	-3.502349
C	7.919058	-5.872133	-1.449387
H	7.993257	-6.927309	-1.726324
H	6.889562	-5.671797	-1.148386
H	8.56015	-5.700236	-0.581148
C	5.701108	-3.022617	-1.874401
C	4.779922	-2.205487	-1.188991
H	5.135567	-1.381807	-0.58443
C	3.417609	-2.458191	-1.336182
C	1.149352	-2.371194	-1.333294
C	-0.191826	-2.009808	-1.170268
H	-0.482488	-1.18721	-0.532932
C	-1.159639	-2.757549	-1.851466
C	-0.784107	-3.864307	-2.648516
H	-1.56232	-4.440341	-3.132397
C	0.539897	-4.228976	-2.793426
H	0.812328	-5.084163	-3.401015
C	1.520982	-3.471383	-2.15206
C	2.955886	-3.527985	-2.154939
C	3.875063	-4.347658	-2.807413
H	3.534888	-5.15967	-3.439686
C	5.226723	-4.094428	-2.665508
H	5.927659	-4.703136	-3.212481

C	-7.095025	-1.567133	-0.799369
C	-8.237361	-2.349186	-1.082317
C	-9.261166	-1.455117	-1.423625
C	-8.711678	-0.146343	-1.337785
C	-9.28684	1.119421	-1.438612
H	-10.343463	1.199238	-1.661827
C	-8.564635	2.277031	-1.203408
C	-8.998527	3.631768	-1.083697
C	-7.882079	4.388394	-0.742541
C	-6.768716	3.491066	-0.682738
C	-8.315987	-3.843808	-0.96983
H	-7.32866	-4.267493	-1.172738
H	-8.982276	-4.229706	-1.747157
C	-8.794665	-4.334092	0.406908
H	-8.881846	-5.42346	0.41873
H	-9.770736	-3.911111	0.65887
H	-8.08987	-4.042603	1.18744
C	-10.651158	-1.800252	-1.859238
H	-11.344891	-1.010013	-1.555565
H	-10.97905	-2.708822	-1.347047
C	-10.746022	-2.007736	-3.381539
H	-10.075489	-2.807159	-3.705079
H	-10.456437	-1.098566	-3.913752
H	-11.764199	-2.272192	-3.678405
C	-10.408498	4.104784	-1.258973
H	-10.849207	3.629487	-2.141609
H	-10.403726	5.179199	-1.460518
C	-11.296869	3.819686	-0.034101
H	-11.35947	2.747202	0.163655
H	-12.309837	4.198166	-0.192692
H	-10.889722	4.294095	0.86098
C	-7.82582	5.874381	-0.537383
H	-8.398386	6.36727	-1.330377
H	-6.792971	6.20997	-0.652711
C	-8.366079	6.33621	0.825514
H	-7.830581	5.854378	1.646978
H	-9.42396	6.087851	0.934921
H	-8.260195	7.41817	0.939236
C	-5.360471	3.807752	-0.426827
C	-4.368929	3.272082	-1.254783
H	-4.633411	2.666977	-2.10796
C	-3.0443	3.484244	-0.911671
C	-0.788283	3.407435	-0.799037
C	0.559521	3.136345	-1.016401

H	0.888605	2.528544	-1.850238
C	1.484198	3.640992	-0.102464
C	1.058103	4.406824	1.003198
H	1.800899	4.762642	1.70298
C	-0.282631	4.688945	1.203416
H	-0.593886	5.280681	2.056438
C	-1.227525	4.189789	0.303089
C	-2.669574	4.242043	0.228321
C	-3.672211	4.814157	1.017551
H	-3.416983	5.397617	1.894424
C	-5.001738	4.6004	0.687458
H	-5.780088	4.994959	1.326498
B	-3.043742	-2.796498	0.65069
B	-6.35675	0.917802	-1.028145
B	3.235267	1.702274	1.542665
H	7.16427	0.095893	-0.719525

Table S11: Cartesian coordinates of the S_0 optimized geometry of **4·4BF$_2$** optimized at B3LYP/6-311G(d,p) level of theory.

* Sum of imaginary frequencies = 0

*Total Energy (hartree) = -5528.413502 Hartree

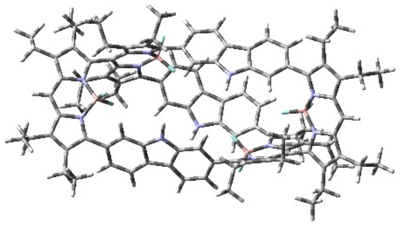

Optimized structure Coordinates:

Symbol	X	Y	Z
F	6.894404	-1.303407	0.733174
F	5.337207	-0.438646	-0.721509
F	2.232745	1.532514	1.379823
F	2.999931	3.553679	2.180386
F	-5.585768	0.823817	-0.040662
F	-5.932507	0.301781	-2.258262
F	-3.253565	-3.952594	1.974059
F	-2.184784	-1.974191	1.462905
N	7.145746	-1.845742	-1.597093
N	7.569729	0.472613	-0.763151
N	4.546243	1.709501	2.171222
N	3.767732	2.765132	0.021643
N	-1.906358	2.500661	-1.984113
H	-1.912684	1.653077	-2.525704
N	-7.277828	2.05707	-1.28209
N	-7.615404	-0.341501	-0.664405
N	-4.504247	-1.894545	2.179911
N	-3.860254	-2.796146	-0.054727
N	-0.27608	1.189613	4.373832
H	-0.49919	2.025903	3.857375
N	1.689466	-2.217435	-2.134103
H	1.657703	-1.275852	-2.486729
C	6.591424	-3.045792	-1.883642
C	7.553031	-3.869999	-2.548467
C	8.720554	-3.122553	-2.663917
C	8.466105	-1.855963	-2.058054
C	9.285308	-0.749698	-1.915941
H	10.290197	-0.790834	-2.317591
C	8.850633	0.422907	-1.299519
C	9.44917	1.703893	-1.145806
C	8.501187	2.522244	-0.516841
C	7.357036	1.721017	-0.300116
C	7.304054	-5.250241	-3.082753
H	7.703276	-5.320323	-4.100462
C	7.923093	-6.366678	-2.226895
H	7.546585	-6.330684	-1.202145

H	9.010356	-6.272359	-2.181033
H	7.686473	-7.350133	-2.641226
C	10.01525	-3.536143	-3.292478
H	9.833693	-4.372649	-3.972367
H	10.403824	-2.718743	-3.90902
C	11.081302	-3.941144	-2.258112
H	11.317308	-3.10822	-1.591938
H	12.003284	-4.255845	-2.753587
H	10.726503	-4.766073	-1.637074
C	10.852187	2.07192	-1.518082
H	10.889473	3.126491	-1.804571
H	11.166146	1.504588	-2.399613
C	11.841367	1.818972	-0.36615
H	11.84521	0.76308	-0.085273
H	11.559203	2.394057	0.518841
H	12.857458	2.10396	-0.651114
C	8.628531	3.972527	-0.151386
H	9.6609	4.180814	0.144693
C	8.212999	4.935854	-1.275515
H	8.3813	5.973271	-0.975309
H	7.153891	4.820259	-1.512169
H	8.784734	4.747974	-2.188152
C	6.059672	2.062506	0.318408
C	5.845441	1.730905	1.653658
C	6.725786	1.16487	2.639629
C	5.935173	0.789461	3.714722
C	4.578688	1.090034	3.375421
C	8.217162	1.017169	2.586037
H	8.553203	0.857175	1.565974
H	8.491756	0.111662	3.132161
C	8.956324	2.222702	3.18845
H	8.701005	3.141952	2.658263
H	10.039169	2.082921	3.125557
H	8.690624	2.36216	4.239144
C	6.413951	0.261304	5.03761
H	5.97318	-0.714309	5.254706
H	7.492251	0.097598	4.983449
C	6.101937	1.217292	6.200181
H	5.024959	1.363537	6.303924
H	6.558122	2.195814	6.029165
H	6.483791	0.820046	7.14454
C	3.37491	0.610681	4.061801
C	2.206273	1.37158	4.14395
H	2.203724	2.408036	3.841507

C	1.028231	0.713428	4.460548
C	0.989392	-0.669558	4.770523
C	2.18581	-1.389794	4.803416
H	2.18611	-2.451348	5.020729
C	3.359749	-0.754379	4.441185
H	4.262352	-1.338262	4.33391
C	-0.401542	-1.057891	4.772627
C	-1.05226	-2.292418	4.835977
H	-0.501443	-3.192154	5.083254
C	-2.387928	-2.371677	4.478551
H	-2.876415	-3.333548	4.41011
C	-3.090541	-1.221426	4.073604
C	-2.490119	0.039474	4.114134
H	-3.025834	0.909714	3.754678
C	-1.144582	0.106663	4.44823
C	-4.391015	-1.314032	3.391564
C	-5.624379	-0.714288	3.750439
C	-6.510173	-0.922431	2.694122
C	-5.790085	-1.660971	1.693201
C	-5.891636	-0.050291	5.069575
H	-6.824767	0.514329	5.015613
H	-5.102645	0.677332	5.283203
C	-5.966057	-1.058843	6.227485
H	-6.761849	-1.788095	6.054139
H	-6.163303	-0.553349	7.176921
H	-5.025281	-1.605519	6.322289
C	-7.907276	-0.371086	2.650115
H	-8.387095	-0.565908	3.615488
H	-8.50699	-0.890292	1.907129
C	-7.94258	1.141886	2.367099
H	-8.975154	1.491701	2.282472
H	-7.417452	1.374207	1.443359
H	-7.456425	1.702857	3.168302
C	-6.096751	-1.981401	0.365693
C	-5.155946	-2.546852	-0.504785
C	-5.183334	-2.785813	-1.918932
C	-3.885741	-3.145759	-2.287008
C	-3.093999	-3.13708	-1.108054
C	-6.349382	-2.713779	-2.859587
H	-5.986333	-2.366225	-3.830455
H	-7.063446	-1.964963	-2.530512
C	-7.060609	-4.064607	-3.039255
H	-7.906017	-3.9656	-3.726002
H	-7.440415	-4.435918	-2.086001

H	-6.380495	-4.818462	-3.442393
C	-3.394534	-3.553509	-3.645459
H	-2.457508	-3.036156	-3.871325
H	-4.114062	-3.232756	-4.402979
C	-3.160111	-5.068486	-3.765171
H	-4.080499	-5.621404	-3.561695
H	-2.406308	-5.398626	-3.04728
H	-2.814865	-5.333744	-4.768363
C	-1.651674	-3.431942	-1.013526
C	-1.190166	-4.55916	-0.303558
H	-1.908221	-5.164595	0.231593
C	0.159831	-4.872597	-0.263644
H	0.498217	-5.74373	0.285094
C	1.078846	-4.044567	-0.914936
C	2.520421	-4.039944	-1.046356
C	3.546879	-4.883291	-0.611456
H	3.320528	-5.777223	-0.042382
C	4.863794	-4.557554	-0.9014
H	5.664682	-5.189184	-0.540398
C	5.188178	-3.377262	-1.606122
C	4.172972	-2.531947	-2.063734
H	4.414912	-1.625866	-2.599145
C	2.858847	-2.874869	-1.784602
C	0.603854	-2.894504	-1.598239
C	-0.751342	-2.592302	-1.670845
H	-1.107201	-1.708665	-2.186302
C	-7.415815	-1.578965	-0.164872
C	-8.618199	-2.318755	-0.203648
C	-9.592932	-1.471415	-0.748131
C	-8.95282	-0.233835	-1.029515
C	-8.793233	-3.713858	0.320742
H	-7.858786	-4.265234	0.185243
H	-9.547871	-4.234115	-0.276407
C	-9.187957	-3.761632	1.806199
H	-10.111639	-3.205264	1.986233
H	-8.404949	-3.32325	2.427652
H	-9.341433	-4.793366	2.132517
C	-11.020666	-1.812701	-1.041814
H	-11.389014	-2.517354	-0.2912
H	-11.644522	-0.917834	-0.953651
C	-11.193544	-2.421897	-2.444736
H	-12.239292	-2.676232	-2.635921
H	-10.594791	-3.32972	-2.548149
H	-10.863034	-1.719484	-3.21361

C	-9.446678	0.996562	-1.460648
H	-10.50275	1.088388	-1.681257
C	-8.645627	2.120734	-1.553895
C	-8.987923	3.485266	-1.800991
C	-7.823758	4.230062	-1.654576
C	-6.772538	3.309746	-1.338578
C	-10.344606	3.977277	-2.201117
H	-11.113269	3.318518	-1.786678
H	-10.520667	4.962387	-1.760293
C	-10.514183	4.062373	-3.728019
H	-11.516655	4.410013	-3.990538
H	-10.356812	3.084706	-4.189723
H	-9.788623	4.754167	-4.161294
C	-7.659545	5.700752	-1.910653
H	-8.649388	6.158741	-1.984799
H	-7.164411	6.184803	-1.065488
C	-6.861384	5.995653	-3.191491
H	-6.780331	7.07264	-3.359697
H	-7.346518	5.547674	-4.062324
H	-5.852293	5.585447	-3.121781
C	-5.352135	3.608671	-1.127858
C	-4.975614	4.642026	-0.240241
H	-5.746012	5.198203	0.277037
C	-3.642024	4.90566	0.032921
H	-3.375254	5.68355	0.738764
C	-2.650484	4.140885	-0.589359
C	-1.206991	4.095104	-0.514291
C	-0.242285	4.796168	0.215935
H	-0.535284	5.591686	0.891467
C	1.095535	4.461324	0.078467
H	1.847726	4.975369	0.658393
C	1.503047	3.430986	-0.796161
C	0.554233	2.721992	-1.536275
H	0.863042	1.909313	-2.181635
C	-0.786239	3.054131	-1.384227
C	-3.044152	3.122351	-1.497605
C	-4.373618	2.851156	-1.778794
H	-4.653056	2.051295	-2.448777
C	2.928916	3.117657	-0.983082
C	3.644149	3.137023	-2.203844
C	4.96565	2.770853	-1.919076
C	5.030889	2.541764	-0.511519
C	3.104753	3.602741	-3.526676
H	3.759511	3.256167	-4.329735

H	2.125718	3.156586	-3.717525
C	2.973449	5.133044	-3.594822
H	2.589072	5.453534	-4.567274
H	3.94339	5.610807	-3.43374
H	2.290795	5.493569	-2.822153
C	6.022821	2.572997	-2.968048
H	5.948138	3.381467	-3.70271
H	7.018819	2.645606	-2.540109
C	5.883269	1.219917	-3.688395
H	4.914693	1.141384	-4.188845
H	5.958301	0.399972	-2.976139
H	6.667681	1.099882	-4.440726
B	3.325764	2.382151	1.460208
B	-6.524454	0.705802	-1.047534
B	-3.381411	-2.667473	1.41977
B	6.663669	-0.78907	-0.5403
H	6.226633	-5.409958	-3.162878
H	8.010815	4.171357	0.728529
